# Discovery of a First-in-Class Degrader for the Lipid Kinase PIKfyve

**DOI:** 10.1021/acs.jmedchem.3c00912

**Published:** 2023-08-21

**Authors:** Chungen Li, Yuanyuan Qiao, Xia Jiang, Lianchao Liu, Yang Zheng, Yudi Qiu, Caleb Cheng, Fengtao Zhou, Yang Zhou, Weixue Huang, Xiaomei Ren, Yuzhuo Wang, Zhen Wang, Arul M. Chinnaiyan, Ke Ding

**Affiliations:** † State Key Laboratory of Chemical Biology, Shanghai Institute of Organic Chemistry, Chinese Academy of Sciences, #345 Lingling Roadd, Shanghai 200032, People’s Republic of China; ‡ Michigan Center for Translational Pathology, University of Michigan, Ann Arbor, Michigan 48109, United States; § The Vancouver Prostate Centre, Vancouver General Hospital and Department of Urologic Sciences, The University of British Columbia, Vancouver, British Columbia V6H 3Z6, Canada; ∥ Department of Pathology, University of Michigan, Ann Arbor, Michigan 48109, United States; ⊥ Howard Hughes Medical Institute, University of Michigan, Ann Arbor, Michigan 48109, United States; # Department of Urology, University of Michigan, Ann Arbor, Michigan 48109, United States; ∇ Institute of Basic Medicine and Cancer (IBMC), Chinese Academy of Sciences, Hangzhou, Zhejiang 310022, People’s Republic of China; ○ International Cooperative Laboratory of Traditional Chinese Medicine Modernization and Innovative Drug Discovery of Chinese Ministry of Education (MOE), Guangzhou City Key Laboratory of Precision Chemical Drug Development, College of Pharmacy, Jinan University, 855 Xingye Avenue East, Guangzhou 511400, People’s Republic of China

## Abstract

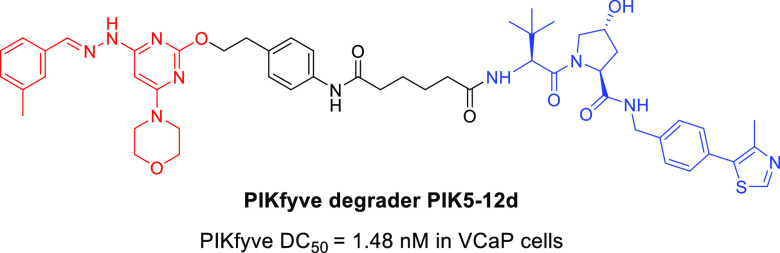

The phosphoinositide kinase PIKfyve has emerged as a new potential therapeutic target in various cancers. However, limited clinical progress has been achieved with PIKfyve inhibitors. Here, we report the discovery of a first-in-class PIKfyve degrader **12d (PIK5-12d)** by employing the proteolysis-targeting chimera approach. **PIK5-12d** potently degraded PIKfyve protein with a DC_50_ value of 1.48 nM and a *D*_max_ value of 97.7% in prostate cancer VCaP cells. Mechanistic studies revealed that it selectively induced PIKfyve degradation in a VHL- and proteasome-dependent manner. PIKfyve degradation by **PIK5-12d** caused massive cytoplasmic vacuolization and blocked autophagic flux in multiple prostate cancer cell lines. Importantly, **PIK5-12d** was more effective in suppressing the growth of prostate cancer cells than the parent inhibitor and exerted prolonged inhibition of downstream signaling. Further, intraperitoneal administration of **PIK5-12d** exhibited potent PIKfyve degradation and suppressed tumor proliferation in vivo. Overall, **PIK5-12d** is a valuable chemical tool for exploring PIKfyve-based targeted therapy.

## 1 Introduction

**Figure 1:**
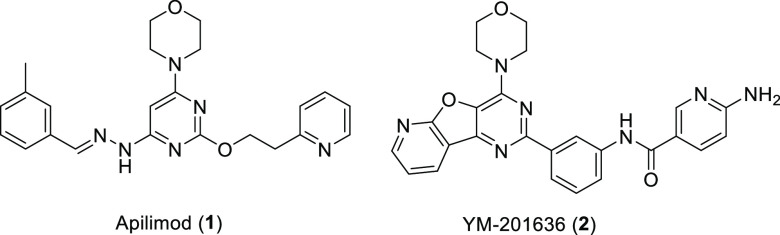
Chemical structures of selected PIKfyve inhibitors.

PIKfyve is a phosphoinositide kinase that is characterized by the presence of a FYVE finger-containing domain structure.^1^ As a lipid kinase, PIKfyve phosphorylates phosphatidylinositol-3-phosphate (PI(3)P) to produce phosphatidylinositol-3,5-bisphosphate (PI(3,5)P_2_), which is crucial to maintain endomembrane homeostasis.^2^ PIKfyve plays a critical role in regulating membrane homeostasis, endosomal trafficking, and autophagy in the endosomal and lysosomal system.^3−6^ Accumulating evidence suggests that PIKfyve is a potential therapeutic target for various human cancers.^7−9^ For example, shRNA knockdown of PIKfyve induced cytoplasmic vacuolization in dividing cells and suppressed cell proliferation.^10^ Apilimod (**1**, Figure 1), a potent and highly selective PIKfyve inhibitor, effectively inhibited the proliferation of B-cell non-Hodgkin lymphoma cells.^11^ Our previous work also demonstrated that the inhibition of PIKfyve could suppress autophagy and potentiate response to immune checkpoint blockade in prostate cancer.^12^

Several selective small-molecule inhibitors of PIKfyve have been disclosed and YM201636 (**2**) and **1** were the most well-characterized examples (Figure 1).^11,13^ Compound **2**, an initially discovered PIKfyve inhibitor, potently inhibited the kinase activity with an IC_50_ value of 33 nM and was selective over several other lipid kinase family members.^13−15^ Compound **1**, as mentioned above, was another small-molecule PIKfyve inhibitor with both good kinase inhibitory activity (IC_50_ = 14 nM) and high kinome selectivity.^11^ Compound **1** has been advanced into clinical trials for the treatment of lymphoma, autoimmune diseases, neurodegenerative diseases, and COVID-19 disease.^11,16−19^ However, due to compound stability issues in vivo, the effects in clinical trials have been limited. Therefore, the discovery of new PIKfyve modulators is highly desirable and thus we explored the potential of PIKfyve-based targeted therapy.

Proteolysis targeting chimera (PROTAC), a heterobifunctional molecule recruiting protein-of-interest (POI) to the E3 ligase and inducing POI degradation by the ubiquitin-proteasome system (UPS), has become a novel paradigm for drug discovery.^20^ The POI degradation mediated by PROTACs is a catalytic and event-driven process, which is usually more efficient than the inhibitor occupancy. Meanwhile, PROTACs can deplete both the catalytic and non-catalytic functions of the kinase, potentially outperforming kinase inhibitors.^21^ Herein, we report the discovery of the first series of PIKfyve PROTAC degraders. The optimal compound **12d** (**PIK5-12d**) showed potent degradative activity against PIKfyve with a DC_50_ value of 1.48 nM and a *D*_max_ value of 97.7%, respectively, in prostate cancer VCaP cells. It also exhibited promising PIKfyve degradative effects in vivo. Importantly, **PIK5-12d** exerted prolonged inhibition of PIKfyve downstream signaling and outperformed the parent PIKfyve inhibitor in the suppression of the growth of prostate cancer cells.

## 2 Results and Discussion

### 2.1 Design of PIKfyve PROTAC Degraders

**Figure 2:**
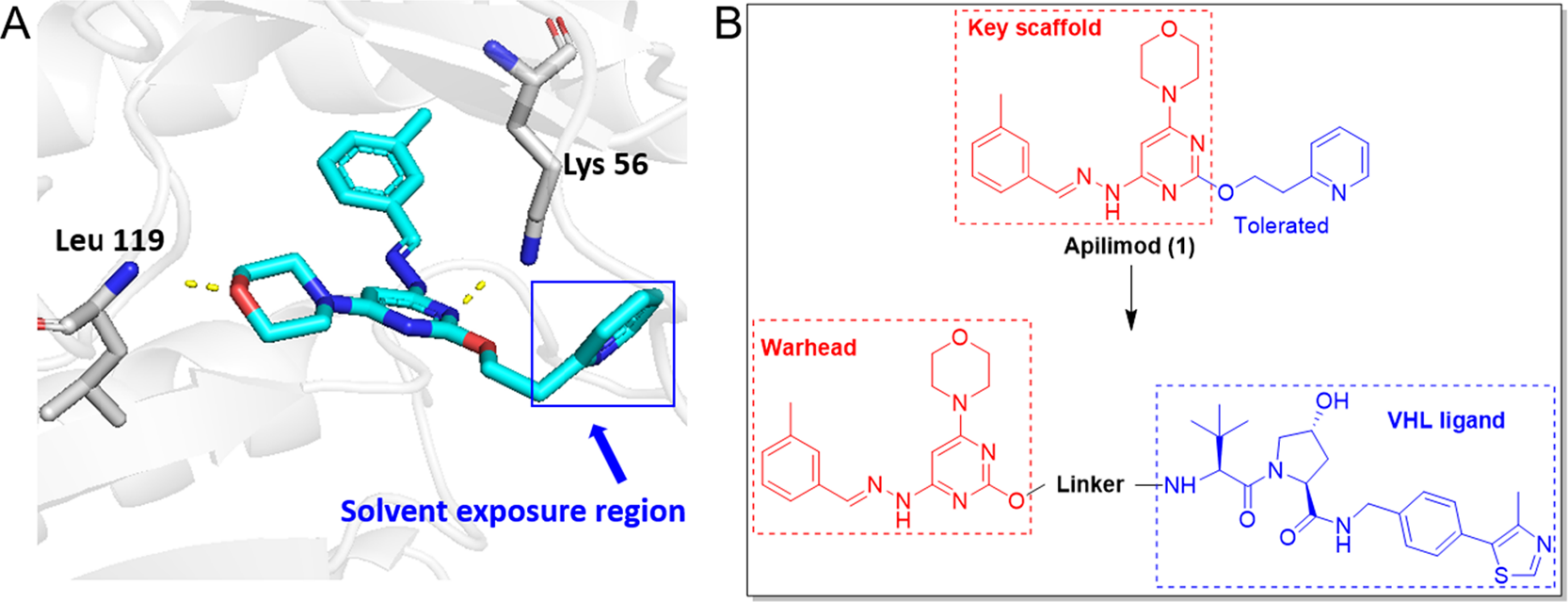
Design of PIKfyve PROTACs. (A) Docking model of compound **1** with PIKfyve protein (PDB: 7K2V); (B) chemical structures of compound **1** and the designed PIKfyve PROTACs.

We chose to utilize compound **1**, a PIKfyve inhibitor in Phase II clinical trials, as the “warhead” to develop PIKfyve PROTAC degraders. The computational modeling studies revealed that the pyridyl moiety of compound **1** extended to the solvent-exposed region (Figure 2A). The reported structure–activity relationship (SAR) studies on compound **1** also indicated that the pyridyl group was well tolerated to various substituents.^22^ Based on these observations, a series of PIKfyve degraders were designed by tethering compound **1** to a ligand for the E3 ligase von Hippel–Lindau (VHL) via a diverse set of linkers (Figure 2B).

### 2.2 Chemical Synthesis

**Scheme 1:**
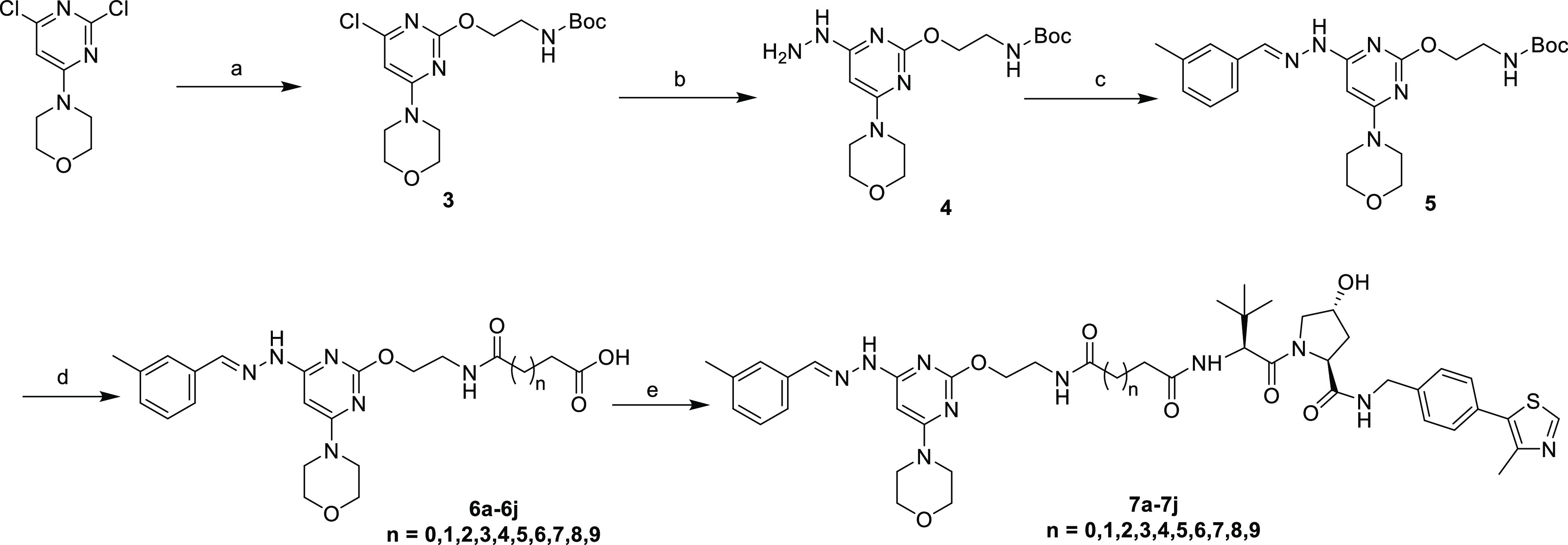
Synthesis of Compounds **7a**–**7j** Reagents and conditions: (a) *tert*-butyl-*N*-(2-hydroxyethyl)carbamate, NaH, *N*, *N*-dimethylformamide (DMF), 0 °C, and 8 h; (b) hydrazine hydrate, 1,4-dioxane, 90 °C, and 12 h; (c) *m*-tolualdehyde, acetic acid (AcOH), ethyl alcohol (EtOH), reflux, and 6 h; (d) (i) trifluoroacetic acid (TFA), dichloromethane (CH_2_Cl_2_), rt., and 3 h; (ii) 2-(7-azabenzotriazol-1-yl)-*N′*, *N′*, *N′*-tetramethyluronium hexafluorophosphate (HATU), triethylamine (Et_3_N), DMF, rt., and 5 h; (iii) TFA, CH_2_Cl_2_, rt., and 3 h; (e) HATU, Et_3_N, DMF, rt., and 5 h.

The synthesis of compounds **7a**–**7j** is depicted in Scheme 1. The starting material 4-(2,6-dichloropyrimidin-4-yl)morpholine went through a regioselective nucleophilic substitution with *tert*-butyl-*N*-(2-hydroxyethyl)carbamate to produce compound **3**, which reacted with hydrazine hydrate to give compound **4**. The subsequent condensation reaction of compound **4** with *m*-tolualdehyde afforded compound **5**, which was acylated by a series of linkers after the deprotection of *N*-Boc to yield key intermediates **6a**–**6j**. Subsequent deprotection of *tert*-butyl ester of compounds **6a**–**6j** and a further amide coupling reaction with the classical VHL ligand produced final compounds **7a**–**7j**.

**Scheme 2:**
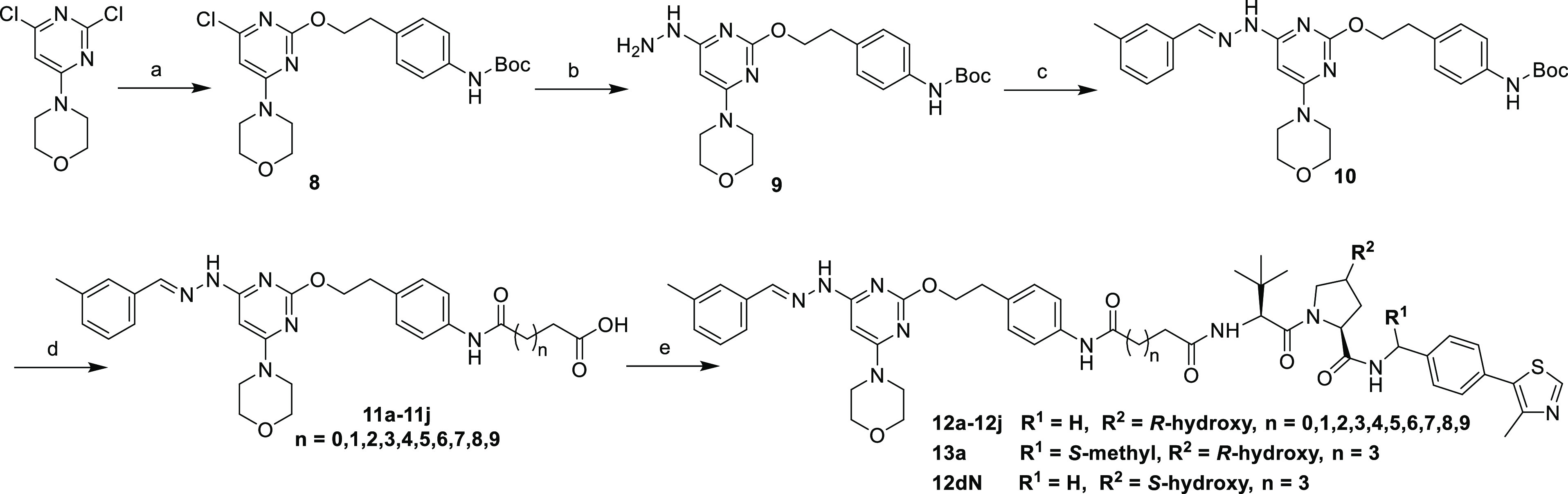
Synthesis of Compounds **12a**–**12j**, **13a**, and **12dN** Reagents and conditions: (a) *tert*-butyl-(4-(2-hydroxyethyl)phenyl)carbamate, NaH, DMF, 0 °C, and 6 h; (b) hydrazine hydrate, 1,4-dioxane, 90 °C, and 12 h; (c) *m*-tolualdehyde, AcOH, EtOH, reflux, and 6 h; (d) (i) TFA, CH_2_Cl_2_, rt., and 3 h; (ii) HATU, Et_3_N, DMF, rt., and 5 h; (iii) TFA, CH_2_Cl_2_, rt., and 3 h; (e) HATU, Et_3_N, DMF, rt., and 5 h.

Compounds **12a**–**12j**, **13a**, and **12dN** were synthesized by the same reactions and conditions used in the synthesis of compounds **7a**–**7j** (Scheme 2). The major difference was that intermediate **8** was constructed from *tert*-butyl-(4-(2-hydroxyethyl)phenyl) carbamate instead of *tert*-butyl-*N*-(2-hydroxyethyl)carbamate.

### 2.3 Structure–Degradation Relationship Study of PIKfyve Degraders

Based on the design strategy noted above, we first obtained a set of PIKfyve degraders by connecting the VHL ligand directly to the central pyrimidine ring via linkers of different lengths (Table 1). The degradation efficiency of these degraders was assessed by immunoblotting assays in VCaP cells with high expression levels of PIKfyve.^12^ The results showed that only compounds with longer linkers displayed good PIKfyve degradative activities. For example, compounds **7i** and **7j** achieved degradation rates of 72 and 67% at 0.1 μM, respectively, for PIKfyve, while other compounds with shorter linkers were much less active. Interestingly, compounds **7i** and **7j** displayed decreased degradative effect on PIKfyve at 1.0 μM, which may be due to the hook effects.^23^

**Table 1:**
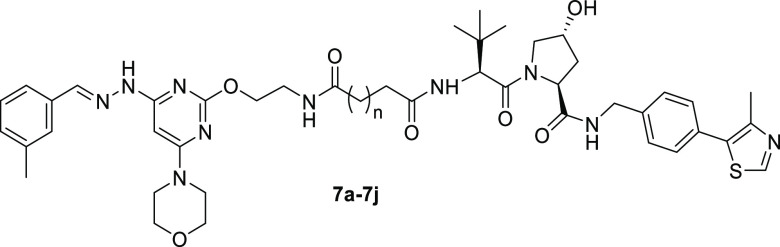
Degradation Efficiency of Compounds **7a**–**7j**^a^

compds	linker (*n*)	% degradation (VCaP)	
		0.1 μM	1.0 μM
**7a**	0	0	0
**7b**	1	0	0
**7c**	2	0	0
**7d**	3	0	10
**7e**	4	3	0
**7f**	5	0	51
**7g**	6	30	17
**7h**	7	32	31
**7i**	8	72	32
**7j**	9	67	21

a Degradation efficiency was determined by immunoblotting after treatment with compounds in VCaP cells for 24 h.

We reasoned that although the pyridyl group of compound **1** was exposed to the solvent area, its aromatic ring structure may still contribute to its binding with PIKfyve protein. Thus, the second series of degraders were designed by connecting the VHL ligand to the phenyl ring that retained the aromatic ring character of the pyridyl group (Table 2). Investigation of the linker length revealed that compound **12d** (**PIK5-12d**) with 4 −CH_2_– in the middle of the linker showed the best PIKfyve degradative activity with the degradation rates of 97 and 91% at 0.1 and 1 μM, respectively. Interestingly, the substitution of the VHL ligand in **PIK5-12d** with a more potent version resulted in compound **13a** with decreased activity.^24^ We also synthesized compound **12dN** (**PIK5-12dN)** as a negative control by using the inactive isomer of the VHL ligand,^25^ and it turned out to have no degradative activity against PIKfyve as expected (Table 2).

**Table 2:**
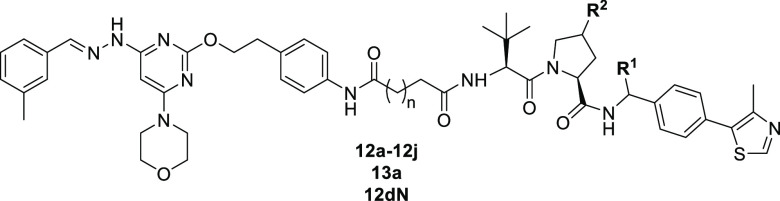
Degradation Efficiency of PIKfyve Degraders **12a**–**12j**, **13a**, and **12dN**^a^

compds	*R*^1^	*R*^2^	linker (*n*)	% degradation (VCaP)	
				0.1 μM	1.0 μM
**12a**	H	*R*-hydroxy	0	0	0
**12b**	H	*R*-hydroxy	1	0	8
**12c**	H	*R*-hydroxy	2	0	0
**12d (PIK5-12d)**	H	*R*-hydroxy	3	97	91
**12e**	H	*R*-hydroxy	4	90	75
**12f**	H	*R*-hydroxy	5	26	19
**12g**	H	*R*-hydroxy	6	84	92
**12h**	H	*R*-hydroxy	7	81	45
**12i**	H	*R*-hydroxy	8	64	41
**12j**	H	*R*-hydroxy	9	48	3
**13a**	*S*-methyl	*R*-hydroxy	3	47	44
**12dN(PIK5-12dN)**	H	*S*-hydroxy	3	0	0

a Degradation efficiency was determined by immunoblotting after treatment with compounds in VCaP cells for 24 h.

### 2.4 Compound **PIK5-12d** Selectively Induced the Degradation of PIKfyve Protein in a Concentration-, Time-, VHL-, and Proteasome-Dependent Manner

**Figure 3:**
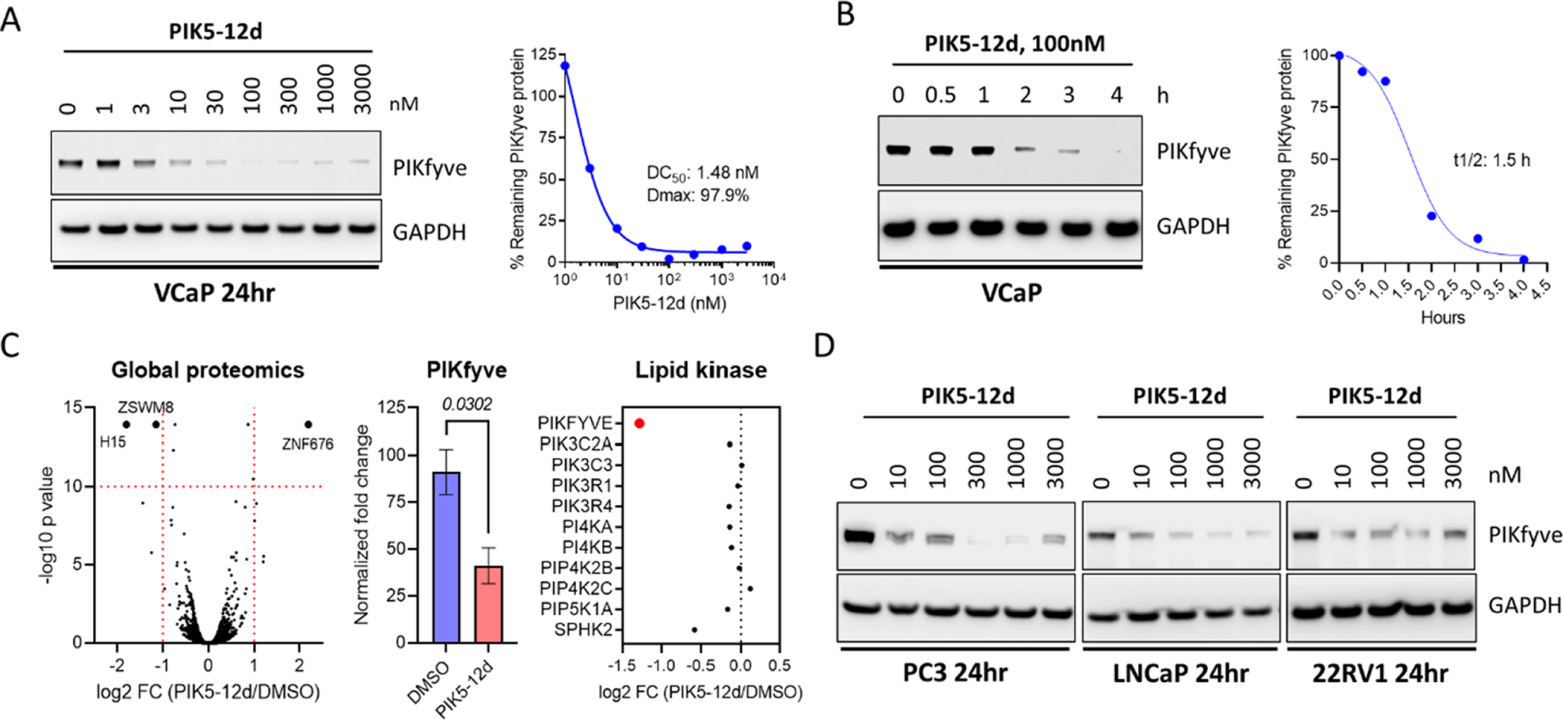
**PIK5-12d** in a concentration-dependent fashion reduced PIKfyve protein in multiple human prostate cancer cells in vitro. (A) Immunoblotting of PIKfyve and GAPDH in VCaP cells treated with increasing concentrations of **PIK5-12d** for 24 h (left), percent remaining PIKfyve protein was plotted for DC_50_ and *D*_max_ determination (right); (B) immunoblotting of PIKfyve and GAPDH in VCaP with 100 nM **PIK5-12d** for various timepoints (left), percent remaining PIKfyve protein was quantified (right); (C) global proteomic analysis of **PIK5-12d** in VCaP cells after 4 h treatment of DMSO or 300 nM **PIK5-12d** (left), mass-spec quantification of PIKfyve protein (middle), lipid kinase changes (right); and (D) immunoblotting of PIKfyve and GAPDH in multiple human prostate cancer cell lines with increasing concentrations of **PIK5-12d** for 24 h.

**PIK5-12d** displayed the most potent degradative effects on PIKfyve in the preliminary screening. To further characterize this compound, we treated VCaP cells with **PIK5-12d** with different concentrations for 24 h. The results showed that compound **PIK5-12d** dose-dependently induced PIKfyve degradation with a DC_50_ value of 1.48 nM and a *D*_max_ value of 97.9% (Figure 3A). The kinetics experiments revealed that **PIK5-12d** at a concentration of 100 nM caused fast degradation of PIKfyve protein with a *t*_1/2_ value of 1.5 h (Figure 3B). We further performed global proteomic analysis using tandem mass tags (TMT) labeled mass-spectrometry to unbiasedly quantify the protein change upon **PIK5-12d** treatment in VCaP cells. The results indicated that **PIK5-12d** is a very specific PIKfyve degrader with only 3 proteins significantly downregulated including PIKfyve, which accounts for the off-target rate at 2 out of 7573 detectable proteins. It is worth noting that **PIK5-12d** is selective for PIKfyve over other lipid kinases (Figure 3C). In addition, **PIK5-12d** also effectively reduced PIKfyve in other prostate cancer PC3, LNCaP, and 22RV1 cells (Figure 3D).

**Figure 4:**
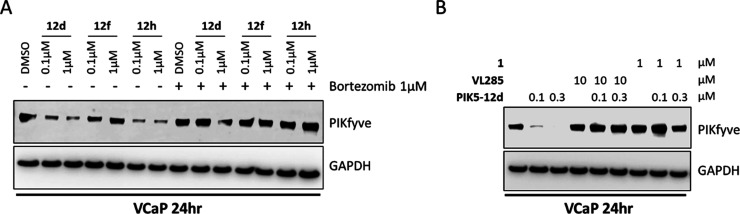
**PIK5-12d** mediated-PIKfyve degradation was VHL and proteasome-dependent. (A) Immunoblotting of PIKfyve and GAPDH in VCaP cells treated with increasing concentrations of **12d** (**PIK5-12d**), **12f**, or **12h** in the conditions of with or without 1 μM of proteasome inhibitor bortezomib for 24 h; (B) immunoblotting of PIKfyve and GAPDH in VCaP cells treated with increasing concentration of **PIK5-12d** with or without warhead **1** or VHL ligand VL285 for 24 h.

We further investigated the mechanism of PIKfyve degradation by **PIK5-12d** in VCaP cells. Western blot results showed that **PIK5-12d** and its analogues (**12f** and **12h**) at 0.1 and 1 μM can degrade PIKfyve protein to different extents, in VCaP cells after treatment for 24 h, while pretreatment with the proteasome inhibitor bortezomib completely rescued the level of PIKfyve protein (Figure 4A). In addition, both the warhead compound **1** and VHL ligand VL285 competitively blocked the PIKfyve degradation by **PIK5-12d** (Figure 4B).^26^ These results demonstrated that **PIK5-12d** induced PIKfyve degradation in a VHL- and proteasome-dependent manner.

### 2.5 **PIK5-12d** Induced Massive Cytoplasmic Vacuolization and Blocked Autophagy in Prostate Cancer Cells

**Figure 5:**
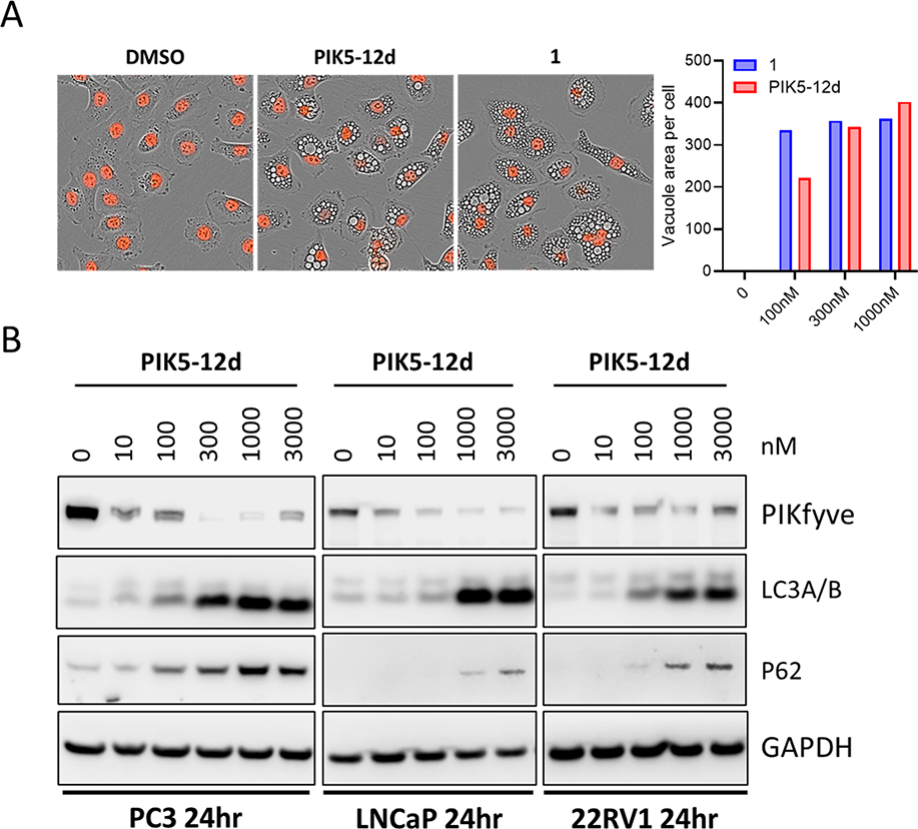
**PIK5-12d** induced massive cytoplasmic vacuolization and blocked autophagy. (A) Representative images of DU145-RFP cells with treatments of DMSO, 300 nM **PIK5-12d**, or **1** for 24 h. The vacuole area per cell is quantified in indicated conditions; (B) immunoblotting of PIKfyve and autophagy markers in indicated human prostate cancer cells with increasing concentrations of **PIK5-12d** for 24 h.

Previous work showed that the PIKfyve inhibition could induce cytoplasmic vacuolization and block autophagy.^12^ Thus, we investigated the effects of **PIK5-12d** on these phenotypes in prostate cancer DU145 cells. The results showed that **PIK5-12d** has comparable vacuolization induction ability to inhibitor **1** in a dose-dependent manner in RFP-labeled DU145 cells (Figure 5A). In addition, **PIK5-12d** also concentration-dependently increased autophagy markers LC3A/B and p62 in different prostate cancer cell lines (Figure 5B).

### 2.6 **PIK5-12d** Decreased Prostate Cancer Cell Proliferation and Exerted Prolonged Suppression of PIKfyve Downstream Signaling

**Figure 6:**
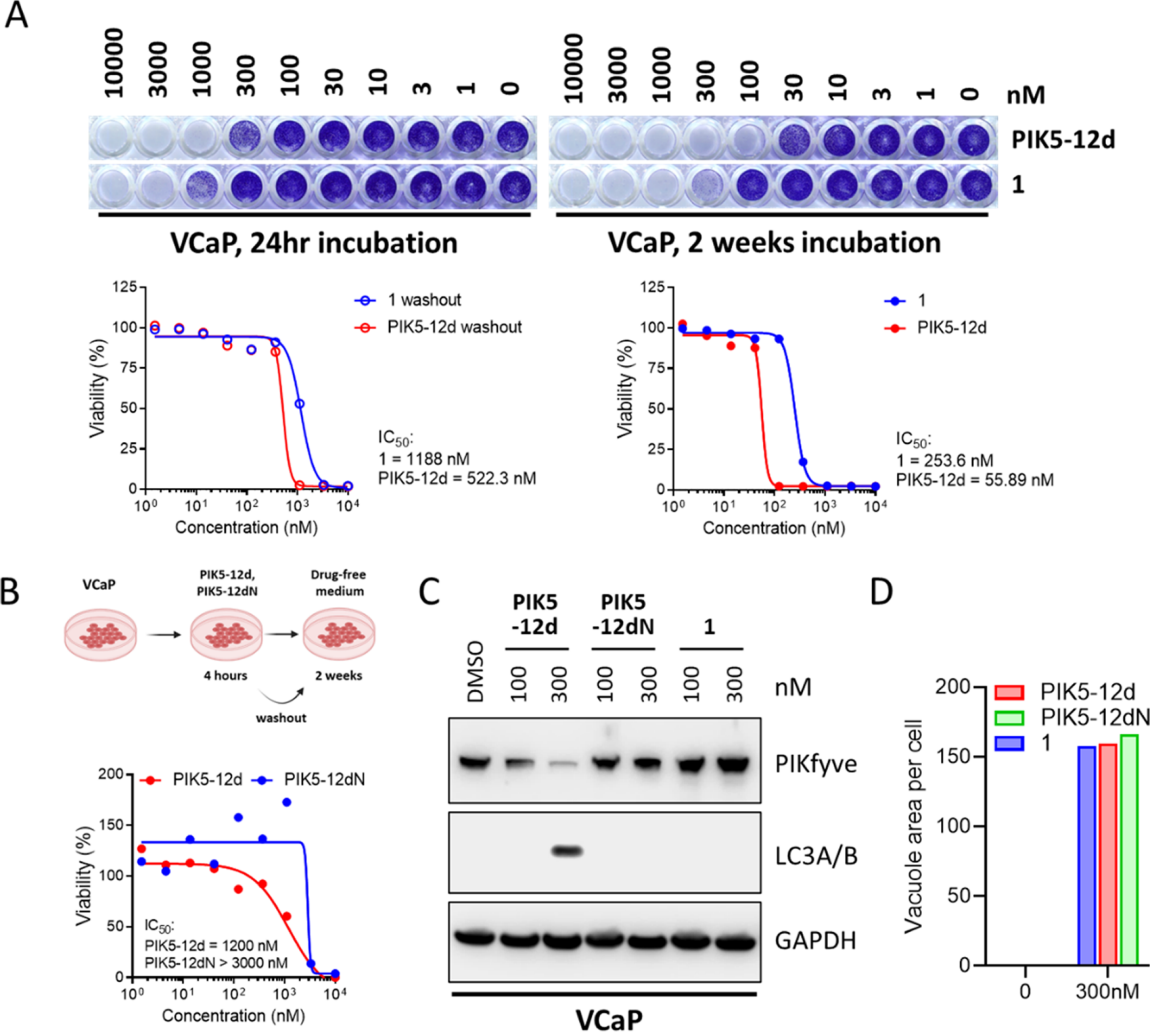
**PIK5-12d** decreased prostate cancer cell proliferation and exerted prolonged suppression of PIKfyve downstream signaling. (A) Long-term cell viability is visualized by crystal violet staining in VCaP either treated with **PIK5-12d** or **1** for 24 h and chased for 2 weeks, or continuous treatment for 2 weeks (top), IC_50_s were calculated for indicated conditions (bottom); (B) long-term cell viability is determined in VCaP cells with washout in drug-free medium after 4 h treatment of **PIK5-12d** or negative control **PIK5-12dN**; and (C) immunoblotting of PIKfyve, LC3A/B, and GAPDH in whole cell lysate of VCaP cells treated with **PIK5-12d**, negative control **PIK5-12dN**, or inhibitor **1** for 4 h and chased in drug-free medium for 72 h. (D) Quantification of vacuole per cell in DU145-RFP cells for 4 h of treatment of **PIK5-12d**, negative control **PIK5-12dN**, or inhibitor **1**.

PROTACs usually have prolonged therapeutic effects due to the irreversible depletion of the target protein.^27^ Thus, we conducted an in vitro washout experiment where VCaP cells were incubated with **PIK5-12d** or **1** for 24 h before compounds were removed. Cells were then cultured in compound-free media for 2 weeks. The results showed that **PIK5-12d** inhibited VCaP cell proliferation with an IC_50_ of 522.3 nM, which is over twofold lower than that of compound **1** (Figure 6A). The anti-proliferative effects were significantly enhanced when VCaP cells were incubated with the compounds for 2 weeks, and **PIK5-12d** was found to be ∼5 times more potent than compound **1** in this experiment. **PIK5-12d** also outperformed the negative control **PIK5-12dN** in long-term anti-proliferation assay after 4 h treatment and followed by culturing in a drug-free medium for 2 weeks (Figure 6B). We further investigated the effect of the compounds on PIKfyve and its downstream signaling using the washout approach. In the experiment, VCaP cells were incubated with **PIK5-12d**, **PIK5-12dN**, or **1** for 4 h, and compounds were then removed. Cells were further cultured in compound-free media for 72 h. It was shown that **PIK5-12d** significantly reduced PIKfyve and increased LC3A/B at 300 nM, while the negative control **PIK5-12dN** and inhibitor **1** did not show any effect on these proteins (Figure 6C). It is worth noting that the vacuolization ability triggered by **PIK5-12d**, negative control **PIK5-12dN**, and inhibitor **1** at 4 h has no significant difference (Figure 6D). These results collectively suggested that **PIK5-12d** exerted prolonged suppression of prostate cancer cell proliferation and PIKfyve downstream signaling.

### 2.7 **PIK5-12d** Depleted PIKfyve Protein and Suppressed Tumor Proliferation In Vivo

**Figure 7:**
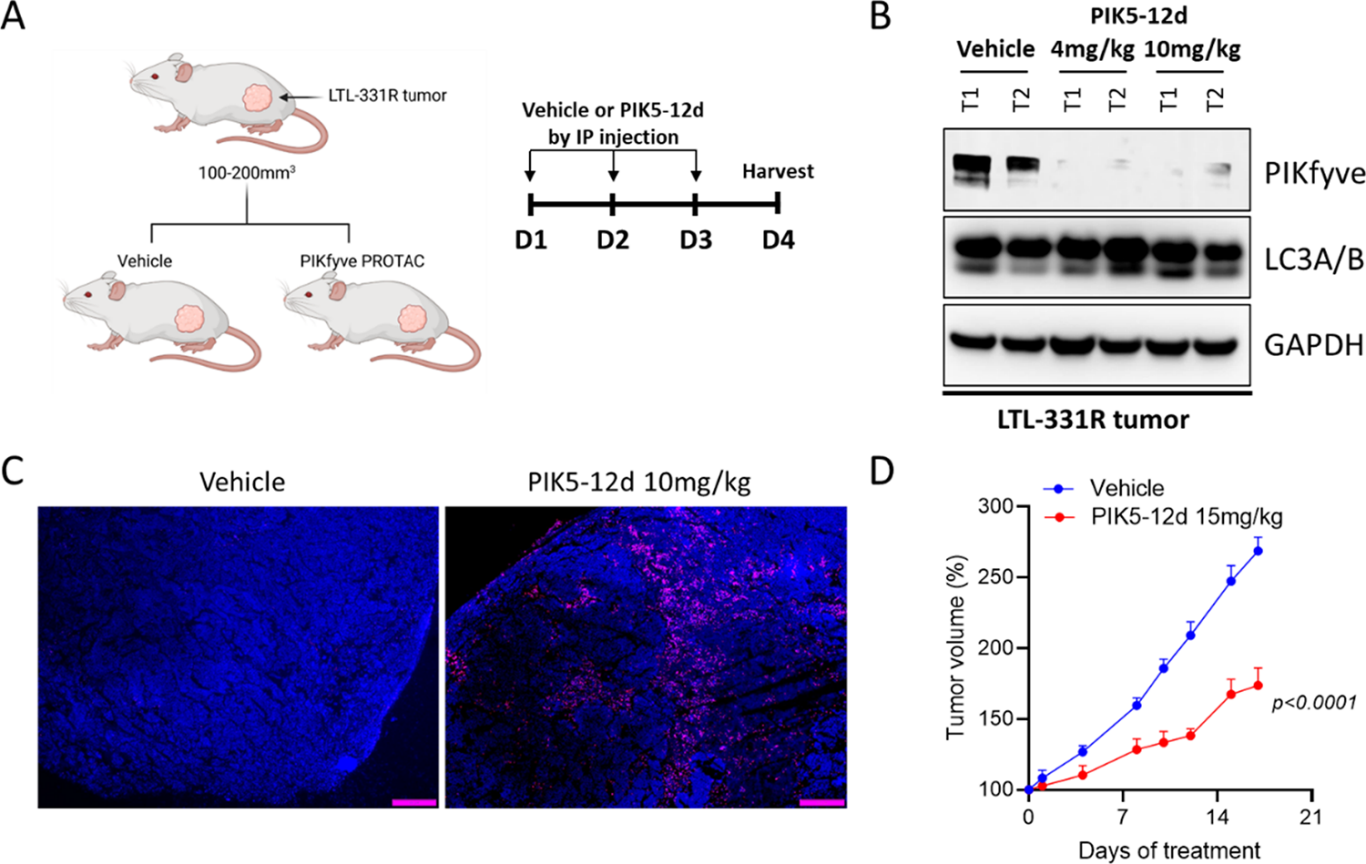
**PIK5-12d** depleted PIKfyve protein in vivo. (A) Study design of pharmacodynamic assessment on target engagement of PIKfyve PROTAC degrader in LTL-331R human prostate cancer patient-derived xenograft model; (B) immunoblotting of PIKfyve, LC3A/B, and GAPDH in the whole cell lysate of human LTL-331R tumors treated with vehicle or various concentrations of **PIK5-12d** for 4 days; (C) representative images of TUNEL signal of LTL-331R tumors treated with vehicle or 10 mg/kg **PIK5-12d** for 4 days; and (D) tumor proliferation of LTL-331R tumors treated with vehicle or 15 mg/kg **PIK5-12d** at 5 days on and 2 days off regimen for 17 days, the *p* value was determined by two-tailed unpaired *t* test between vehicle and **PIK5-12d** groups.

We further performed a pharmacodynamic assessment of **PIK5-12d** in an LTL-331R human prostate cancer patient-derived xenograft (PDX) model. **PIK5-12d** was administrated by intraperitoneal (IP) injection with two doses of 4 and 10 mg/kg, respectively, for 3 days. The tumor tissues were harvested on day 4 and subjected to western blot analysis (Figure 7A). As shown in Figure 7B, **PIK5-12d** almost completely depleted PIKfyve protein at both doses compared to the vehicle control group, indicating its strong PIKfyve degradation efficiency in vivo. In addition, the depletion of PIKfyve protein by **PIK5-12d** also triggered tumor cell death (Figure 7C). Then, long-term tumor efficacy of the LTL-331R model was determined by once-daily administration of **PIK5-12d** at 5 days on and 2 days off regimen for 17 days. It was shown that **PIK5-12d** significantly suppressed tumor proliferation in vivo (Figure 7D). These results collectively suggested the promising therapeutic potential of PIKfyve degradation for prostate cancer treatment.

## 3 Conclusions

The lipid kinase PIKfyve has been increasingly recognized as a therapeutic target for multiple types of cancer including multiple myeloma, prostate cancer, non-Hodgkin lymphoma, and other diseases such as neurodegenerative disorders and SARS-CoV-2 infection. Although a number of small-molecule inhibitors have been developed, only a few of them advanced in clinical development such as compound **1**. Published data suggested that compound **1** has low plasma stability which limited its in vivo efficacy.^1,11^ Thus, there is an urgent need for the development of other modulators to target PIKfyve.

PROTAC, as a new modulator type, has become a novel paradigm for kinase drug discovery. PROTAC often outperformed kinase inhibitors because it not only functions in a catalytic and event-driven manner but can disrupt both the enzymatic and scaffolding functions of the kinase. Here, we report the discovery of a first-in-class PIKfyve PROTAC degrader **PIK5-12d**. **PIK5-12d** effectively degraded PIKfyve with a DC_50_ value of 1.48 nM and a *D*_max_ value of 97.7% in prostate cancer VCaP cells. Mechanistic studies showed that **PIK5-12d** induced PIKfyve degradation through the VHL- and proteasome-dependent manner. **PIK5-12d** also induced massive cytoplasmic vacuolization and blocked autophagy in prostate cancer cells. Importantly, **PIK5-12d** exerted prolonged suppression of prostate cancer cell proliferation and PIKfyve downstream signaling compared to the kinase inhibitor **1**. In addition, **PIK5-12d** exhibited potent PIKfyve degradation effects, triggered tumor cell death, and suppressed tumor proliferation in vivo. Taken together, this study discovered a first-in-class PIKfyve degrader as the valuable tool compound for the research community and provided the proof-of-concept for the degradation of PIKfyve as a promising therapeutic approach for prostate cancer as well as other cancers.

## 4 Experimental Section

### 4.1 General Methods for Chemistry

The reagents and solvents used in chemical synthesis were obtained from commercial agents without further purification. All reactions were monitored by using thin-layer chromatography (TLC). All final compounds were purified by a column chromatography on silica gel (300–400 mesh). The NMR spectra were recorded on Agilent DD2 500 spectrometer (Agilent Technologies Inc., USA) or Bruker AVANCE 600 spectrometer (Bruker Company, Germany) in CDCl_3_ or DMSO-*d*_6_. The spectra of high-resolution mass (HRMS) were monitored by Bruker MaXis 4G TOF mass spectrometer. The purities of all final compounds were identified by HPLC analysis with the Agilent 1200 system and were proved to be >95%. HPLC condition: Triart C18 reversed-phase column, 5 μm, 4.6 mm × 250 mm, and flow rate 1.0 mL/min, starting with a 15 min-gradient from 0.1% TFA in water and acetonitrile 1:9 mixture to 0.1% TFA in acetonitrile, then ending with 0.1% TFA in acetonitrile for 5 min.

#### 4.1.1 *tert*-Butyl (2-((4-Chloro-6-morpholinopyrimidin-2-yl)oxy)ethyl)carbamate (**3**)

NaH (0.2 g, 7.8 mmol, 60% dispersion in mineral oil) was added into a solution of *tert*-butyl-*N*-(2-hydroxyethyl)carbamate (0.5 g, 3.1 mmol) in anhydrous DMF (10 mL) at 0 °C. The resulting mixture was stirred at 0 °C for 30 min. Then, 4-(2,6-dichloropyrimidin-4-yl)morpholine (0.7 g, 3.1 mmol) dissolved in anhydrous DMF (5 mL) was added dropwise. The reaction solution was stirred at the same temperature for a further 8 h, then was diluted with 50 mL of water to precipitate a white solid, which was filtered and washed with water three times (50 mL per wash). The resulting solid was dried to give a mixture of isomers *tert*-butyl (2-((4-chloro-6-morpholinopyrimidin-2-yl)oxy)ethyl)carbamate and tert-butyl (2-((2-chloro-6-morpholinopyrimidin-4-yl)oxy)ethyl)carbamate and was purified by column chromatography [petroleum ether (PE)/ethyl acetate (EA)] to give specific intermediate **3** (600 mg, 54%). ^1^H NMR (500 MHz, DMSO-*d*_6_) δ 6.97 (t, *J* = 5.8 Hz, 1H), 6.60 (s, 1H), 4.16 (t, *J* = 5.8 Hz, 2H), 3.66–3.55 (m, 8H), 3.22 (q, *J* = 5.8 Hz, 2H), 1.35 (s, 9H).

#### 4.1.2 *tert*-Butyl (2-((4-Hydrazineyl-6-morpholinopyrimidin-2-yl)oxy)ethyl)carbamate (**4**)

A solution of intermediate **3** (0.5 g, 1.4 mmol) and 2.5 mL of 50% hydrazine hydrate solution dissolved in dioxane (15 mL) was stirred at 90 °C for 12 h. Intermediate **3** was exhausted by a monitoring of TLC. The reaction solution was concentrated under reduced pressure to obtain a white solid, which was washed with 50 mL of water and dried to give the intermediate **4** (470 mg, 95%). ^1^H NMR (500 MHz, DMSO-*d*_6_) δ 8.96 (brs, 2H), 7.65 (s, 1H), 6.94 (t, *J* = 5.7 Hz, 1H), 4.05 (t, *J* = 6.0 Hz, 2H), 3.64–3.58 (m, 4H), 3.38 (t, *J* = 4.8 Hz, 4H), 3.18 (q, *J* = 6.0 Hz, 2H), 1.72 (s, 3H), 1.35 (s, 9H).

#### 4.1.3 *tert*-Butyl (E)-(2-((4-(2-(3-Methylbenzylidene)hydrazineyl)-6-morpholinopyrimidin-2-yl)oxy)ethyl)carbamate (**5**)

Intermediate **4** (400 mg, 1.1 mmol) and 3-methylbenzaldehyde (142 mg, 1.2 mmol) were dissolved in anhydrous ethanol (10 mL). Then, a catalytic amount of acetic acid was added. The resulting solution was refluxed for 6 h, cooled to room temperature, and concentrated under reduced pressure to obtain a white solid, which was resuspended into a mixture solution of PE (5 mL) and CH_2_Cl_2_ (5 mL), then was filtered to give intermediate **5** (450 mg, 90%). ^1^H NMR (500 MHz, DMSO-*d*_6_) δ 10.82 (s, 1H), 7.99 (s, 1H), 7.48 (d, *J* = 7.7 Hz, 1H), 7.46 (s, 1H), 7.27 (t, *J* = 7.6 Hz, 1H), 7.15 (d, *J* = 7.5 Hz, 1H), 6.96 (t, *J* = 5.6 Hz, 1H), 6.05 (s, 1H), 4.13 (t, *J* = 5.9 Hz, 2H), 3.65 (t, *J* = 4.9 Hz, 4H), 3.51 (t, *J* = 4.8 Hz, 4H), 3.23 (q, *J* = 5.9 Hz, 2H), 2.32 (s, 3H), 1.36 (s, 9H).

#### 4.1.4 General Procedures for the Synthesis of Intermediates **6a**–**6j**

A solution of intermediate **5** (100 mg, 0.2 mmol) dissolved in CH_2_Cl_2_ (5 mL) was added 1 mL of TFA. The reaction solution was stirred at room temperature for 3 h and was concentrated under reduced pressure to obtain the deprotected intermediate residue, which was redissolved in anhydrous DMF (3.5 mL). Then, 3-(*tert*-butoxy)-3-oxopropanoic acid (48 mg, 0.3 mmol), Et_3_N (202 mg, 2.0 mmol), and HATU (190 mg, 0.5 mmol) were added in order. The resulting solution was stirred at room temperature for 5 h, diluted with 30 mL of water, and extracted with EtOAc for three times. The organic phase was dried with anhydrous Na_2_SO_4_, filtered, and concentrated. The resulting residue was redissolved in 5 mL of CH_2_Cl_2_, which was added 1 mL of TFA. The resulting mixture was stirred at room temperature for another 3 h to deprotect the *tert*-butyl ester. The reaction solution was concentrated to obtain a crude residue, which was purified by chromatography on a silica gel column with CH_2_Cl_2_/MeOH to give white intermediate **6a** (65 mg, 63%). ^1^H NMR (500 MHz, DMSO-*d*_6_) δ 10.97 (brs, 1H), 8.30 (t, *J* = 5.6 Hz, 1H), 8.02 (s, 1H), 7.59–7.46 (m, 2H), 7.28 (t, *J* = 7.6 Hz, 1H), 7.17 (d, *J* = 7.6 Hz, 1H), 6.03 (s, 1H), 4.22 (t, *J* = 5.6 Hz, 2H), 3.66 (t, *J* = 4.7 Hz, 4H), 3.60–3.50 (m, 4H), 3.41 (q, *J* = 5.6 Hz, 2H), 3.14 (s, 2H), 2.33 (s, 3H). Intermediates **6b**–**6j** were obtained according to the procedure of **6a**.

#### 4.1.5 General Procedures for the Synthesis of Intermediates **7a**–**7j**

A solution of intermediate **6a** (50 mg, 0.1 mmol) dissolved in anhydrous DMF (3.5 mL) was added the classical VHL ligand (2*S*,4*R*)-1-((*S*)-2-amino-3,3-dimethylbutanoyl)-4-hydroxy-*N*-(4-(4-methylthiazol-5-yl)benzyl)pyrrolidine-2-carboxamide (42 mg, 0.1 mmol), Et_3_N (30 mg, 0.3 mmol), and HATU (76 mg, 0.2 mmol) in order. The resulting mixture was stirred at room temperature for 5 h, diluted with 15 mL of water, and extracted with EtOAc for two times. The organic phase was dried with anhydrous Na_2_SO_4_, filtered, and concentrated. The resulting residue was purified by chromatography on a silica gel column with CH_2_Cl_2_/MeOH to obtain the final compound **7a** (40 mg, 48%). Compounds **7b**–**7j** were obtained according to the procedure of **7a**.

#### 4.1.6 N^1^-((*S*)-1-((2*S*,4*R*)-4-Hydroxy-2-((4-(4-methylthiazol-5-yl)benzyl)carbamoyl)pyrrolidin-1-yl)-3,3-dimethyl-1-oxobutan-2-yl)-N^3^-(2-((4-(2-((E)-3-methylbenzylidene)hydrazineyl)-6-morpholinopyrimidin-2-yl)oxy)ethyl)malonamide (**7a**)

^1^H NMR (600 MHz, DMSO-*d*_6_) δ 10.85 (s, 1H), 8.98 (s, 1H), 8.59 (t, *J* = 6.1 Hz, 1H), 8.32 (t, *J* = 5.6 Hz, 1H), 8.21 (d, *J* = 9.4 Hz, 1H), 8.02 (s, 1H), 7.51 (d, *J* = 7.6 Hz, 1H), 7.48 (s, 1H), 7.43–7.38 (m, 4H), 7.29 (t, *J* = 7.6 Hz, 1H), 7.17 (d, *J* = 7.6 Hz, 1H), 6.08 (s, 1H), 5.15 (brs, 1H), 4.54 (d, *J* = 9.4 Hz, 1H), 4.46–4.40 (m, 2H), 4.37–4.34 (m, 1H), 4.23 (dd, *J* = 15.9, 5.6 Hz, 1H), 4.20 (t, *J* = 5.8 Hz, 2H), 3.69–3.61 (m, 6H), 3.56–3.51 (m, 4H), 3.44–3.39 (m, 2H), 3.25 (d, *J* = 15.0 Hz, 1H), 3.16 (d, *J* = 15.0 Hz, 1H), 2.45 (s, 3H), 2.34 (s, 3H), 2.08–2.01 (m, 1H), 1.94–1.87 (m, 1H), 0.94 (s, 9H). ^13^C NMR (150 MHz, DMSO-*d*_6_) δ 171.25, 168.64, 166.85, 165.70, 163.71, 163.27, 162.81, 150.82, 147.11, 140.74, 138.86, 137.31, 134.12, 130.54, 129.17, 129.04, 128.04, 127.99, 126.81, 126.42, 122.95, 75.14, 68.26, 65.30, 63.90, 58.09, 55.80, 55.75, 43.61, 42.03, 41.04, 37.70, 37.32, 34.95, 25.62, 20.32, 15.32. HRMS [electrospray ionization (ESI)] calcd for C_43_H_54_N_10_O_7_S [M + H]^+^ 855.3976, found 855.3977. HPLC purity 98.63%.

#### 4.1.7 N^1^-((*S*)-1-((2*S*,4*R*)-4-Hydroxy-2-((4-(4-methylthiazol-5-yl)benzyl)carbamoyl)pyrrolidin-1-yl)-3,3-dimethyl-1-oxobutan-2-yl)-N^4^-(2-((4-(2-((E)-3-methylbenzylidene)hydrazineyl)-6-morpholinopyrimidin-2-yl)oxy)ethyl)succinimide (**7b**)

^1^H NMR (600 MHz, DMSO-*d*_6_) δ 10.84 (s, 1H), 8.98 (s, 1H), 8.57 (t, *J* = 6.0 Hz, 1H), 8.07 (t, *J* = 5.5 Hz, 1H), 8.02 (s, 1H), 7.91 (d, *J* = 9.3 Hz, 1H), 7.50 (d, *J* = 7.8 Hz, 1H), 7.48 (s, 1H), 7.42 (d, *J* = 8.4 Hz, 2H), 7.38 (d, *J* = 8.3 Hz, 2H), 7.29 (t, *J* = 7.6 Hz, 1H), 7.17 (d, *J* = 7.5 Hz, 1H), 6.07 (s, 1H), 5.13 (brs, 1H), 4.52 (d, *J* = 9.4 Hz, 1H), 4.45–4.39 (m, 2H), 4.35 (s, 1H), 4.22 (dd, *J* = 15.9, 5.5 Hz, 1H), 4.17 (t, *J* = 5.8 Hz, 2H), 3.69–3.60 (m, 6H), 3.57–3.50 (m, 4H), 3.41–3.35 (m, 3H), 2.44 (s, 3H), 2.40–2.28 (m, 6H), 2.07–2.01 (m, 1H), 1.93–1.86 (m, 1H), 0.93 (s, 9H). ^13^C NMR (150 MHz, DMSO-*d*_6_) δ 171.31, 171.04, 170.64, 168.96, 163.71, 163.31, 162.82, 150.83, 147.09, 140.72, 138.88, 137.30, 134.13, 130.54, 129.16, 129.01, 128.02, 127.99, 126.80, 126.42, 122.94, 75.11, 68.26, 65.30, 63.93, 58.08, 55.80, 55.69, 43.60, 41.02, 37.60, 37.31, 34.71, 30.24, 29.87, 25.73, 20.31, 15.32. HRMS (ESI) calcd for C_44_H_56_N_10_O_7_S [M + Na]^+^ 891.3952, found 891.3949. HPLC purity 98.73%.

#### 4.1.8 N^1^-((*S*)-1-((2*S*,4*R*)-4-Hydroxy-2-((4-(4-methylthiazol-5-yl)benzyl)carbamoyl)pyrrolidin-1-yl)-3,3-dimethyl-1-oxobutan-2-yl)-N^5^-(2-((4-(2-((E)-3-methylbenzylidene)hydrazineyl)-6-morpholinopyrimidin-2-yl)oxy)ethyl)glutaramide (**7c**)

^1^H NMR (600 MHz, DMSO-*d*_6_) δ 10.85 (s, 1H), 8.98 (s, 1H), 8.57 (t, *J* = 6.1 Hz, 1H), 8.01 (s, 1H), 8.00 (t, *J* = 5.7 Hz, 1H), 7.93 (d, *J* = 9.3 Hz, 1H), 7.50 (d, *J* = 7.8 Hz, 1H), 7.48 (s, 1H), 7.41 (d, *J* = 8.3 Hz, 2H), 7.37 (d, *J* = 8.3 Hz, 2H), 7.29 (t, *J* = 7.6 Hz, 1H), 7.17 (d, *J* = 7.5 Hz, 1H), 6.07 (s, 1H), 5.14 (brs, 1H), 4.58–4.51 (m, 1H), 4.47–4.39 (m, 2H), 4.37–4.34 (m, 1H), 4.25–4.15 (m, 3H), 3.70–3.64 (m, 6H), 3.54 (t, *J* = 4.9 Hz, 4H), 3.42–3.36 (m, 2H), 2.44 (s, 3H), 2.34 (s, 3H), 2.28–2.21 (m, 1H), 2.18–2.11 (m, 1H), 2.08 (t, *J* = 7.6 Hz, 2H), 2.06–2.01 (m, 1H), 1.94–1.87 (m, 1H), 1.75–1.67 (m, 2H), 0.94 (s, 9H). ^13^C NMR (150 MHz, DMSO-*d*_6_) δ 171.45, 171.31, 171.14, 169.21, 163.67, 163.25, 162.81, 150.85, 150.82, 147.08, 140.76, 138.85, 137.28, 134.12, 130.53, 129.15, 129.01, 128.00, 127.97, 126.78, 126.43, 122.95, 75.10, 68.26, 65.30, 63.87, 58.11, 55.82, 55.77, 43.60, 41.03, 37.46, 37.33, 34.55, 34.22, 33.66, 25.78, 21.15, 20.31, 15.31. HRMS (ESI) calcd for C_45_H_58_N_10_O_7_S [M + H]^+^ 883.4289, found 883.4285. HPLC purity 98.43%.

#### 4.1.9 N^1^-((*S*)-1-((2*S*,4*R*)-4-Hydroxy-2-((4-(4-methylthiazol-5-yl)benzyl)carbamoyl)pyrrolidin-1-yl)-3,3-dimethyl-1-oxobutan-2-yl)-N^6^-(2-((4-(2-((E)-3-methylbenzylidene)hydrazineyl)-6-morpholinopyrimidin-2-yl)oxy)ethyl)adipamide (**7d**)

^1^H NMR (600 MHz, DMSO-*d*_6_) δ 10.85 (s, 1H), 8.98 (s, 1H), 8.57 (t, *J* = 6.1 Hz, 1H), 8.04–7.98 (m, 2H), 7.88 (d, *J* = 9.3 Hz, 1H), 7.51 (d, *J* = 7.8 Hz, 1H), 7.48 (s, 1H), 7.41 (d, *J* = 8.3 Hz, 2H), 7.38 (d, *J* = 8.3 Hz, 2H), 7.29 (t, *J* = 7.6 Hz, 1H), 7.17 (d, *J* = 7.5 Hz, 1H), 6.07 (s, 1H), 5.14 (brs, 1H), 4.54 (d, *J* = 9.4 Hz, 1H), 4.46–4.39 (m, 2H), 4.35 (s, 1H), 4.24–4.13 (m, 3H), 3.70–3.63 (m, 6H), 3.57–3.51 (m, 4H), 3.44–3.32 (m, 2H), 2.44 (s, 3H), 2.34 (s, 3H), 2.29–2.23 (m, 1H), 2.13–2.01 (m, 4H), 1.94–1.87 (m, 1H), 1.45 (dt, *J* = 11.7, 5.3 Hz, 4H), 0.93 (s, 9H). ^13^C NMR (150 MHz, DMSO-*d*_6_) δ 171.67, 171.33, 169.13, 163.66, 163.23, 162.76, 150.83, 147.09, 140.79, 138.87, 137.29, 134.11, 130.54, 129.17, 129.01, 128.01, 127.98, 126.79, 126.44, 122.96, 75.09, 68.25, 65.30, 64.03, 58.08, 55.75, 55.71, 43.61, 41.02, 37.46, 37.32, 34.57, 34.45, 34.02, 25.76, 24.53, 24.30, 20.31, 15.32. HRMS (ESI) calcd for C_46_H_60_N_10_O_7_S [M + Na]^+^ 919.4265, found 919.4255. HPLC purity 99.08%.

#### 4.1.10 N^1^-((*S*)-1-((2*S*,4*R*)-4-Hydroxy-2-((4-(4-methylthiazol-5-yl)benzyl)carbamoyl)pyrrolidin-1-yl)-3,3-dimethyl-1-oxobutan-2-yl)-N^7^-(2-((4-(2-((E)-3-methylbenzylidene)hydrazineyl)-6-morpholinopyrimidin-2-yl)oxy)ethyl)heptanediamide (**7e**)

^1^H NMR (600 MHz, DMSO-*d*_6_) δ 10.84 (s, 1H), 8.98 (s, 1H), 8.56 (t, *J* = 6.1 Hz, 1H), 8.02 (s, 1H), 8.00 (t, *J* = 5.6 Hz, 1H), 7.86 (d, *J* = 9.4 Hz, 1H), 7.50 (d, *J* = 7.9 Hz, 1H), 7.48 (s, 1H), 7.42 (d, *J* = 8.3 Hz, 2H), 7.38 (d, *J* = 8.3 Hz, 2H), 7.29 (t, *J* = 7.6 Hz, 1H), 7.17 (d, *J* = 7.5 Hz, 1H), 6.07 (s, 1H), 5.13 (brs, 1H), 4.54 (d, *J* = 9.4 Hz, 1H), 4.46–4.39 (m, 2H), 4.38–4.33 (m, 1H), 4.24–4.15 (m, 3H), 3.70–3.64 (m, 6H), 3.53 (t, *J* = 4.9 Hz, 4H), 3.44–3.38 (m, 2H), 2.44 (s, 3H), 2.34 (s, 3H), 2.27–2.21 (m, 1H), 2.14–2.02 (m, 4H), 1.95–1.86 (m, 1H), 1.52–1.42 (m, 4H), 1.24–1.19 (m, 2H), 0.93 (s, 9H). ^13^C NMR (150 MHz, DMSO-*d*_6_) δ 171.72, 171.42, 171.31, 169.10, 163.65, 163.25, 162.75, 150.81, 147.07, 140.77, 138.86, 137.28, 134.09, 130.53, 129.16, 128.99, 127.99, 127.96, 126.77, 126.41, 122.94, 75.08, 68.23, 65.28, 63.96, 58.06, 55.72, 55.66, 43.59, 41.00, 37.49, 37.31, 34.56, 34.18, 27.73, 25.75, 24.61, 24.40, 20.30, 15.30. HRMS (ESI) calcd for C_47_H_62_N_10_O_7_S [M + Na]^+^ 933.4422, found 933.4414. HPLC purity 98.54%.

#### 4.1.11 N^1^-((*S*)-1-((2*S*,4*R*)-4-Hydroxy-2-((4-(4-methylthiazol-5-yl)benzyl)carbamoyl)pyrrolidin-1-yl)-3,3-dimethyl-1-oxobutan-2-yl)-N^8^-(2-((4-(2-((E)-3-methylbenzylidene)hydrazineyl)-6-morpholinopyrimidin-2-yl)oxy)ethyl)octanediamide (**7f**)

^1^H NMR (600 MHz, DMSO-*d*_6_) δ 10.82 (s, 1H), 8.98 (s, 1H), 8.56 (t, *J* = 6.1 Hz, 1H), 8.01 (s, 1H), 7.99 (t, *J* = 5.5 Hz, 1H), 7.85 (d, *J* = 9.4 Hz, 1H), 7.53–7.45 (m, 2H), 7.42 (d, *J* = 8.3 Hz, 2H), 7.39–7.36 (m, 2H), 7.29 (t, *J* = 7.6 Hz, 1H), 7.17 (d, *J* = 7.5 Hz, 1H), 6.07 (s, 1H), 5.12 (brs, 1H), 4.54 (d, *J* = 9.4 Hz, 1H), 4.46–4.39 (m, 2H), 4.35 (s, 1H), 4.22 (dd, *J* = 15.9, 5.5 Hz, 1H), 4.18 (t, *J* = 5.7 Hz, 2H), 3.70–3.62 (m, 6H), 3.56–3.49 (m, 4H), 3.38–3.35 (m, 2H), 2.44 (s, 3H), 2.34 (s, 3H), 2.27–2.20 (m, 1H), 2.13–2.08 (m, 1H), 2.08–2.05 (m, 2H), 2.05–2.00 (m, 1H), 1.94–1.86 (m, 1H), 1.51–1.41 (m, 4H), 1.25–1.20 (m, 4H), 0.93 (s, 9H). ^13^C NMR (150 MHz, DMSO-*d*_6_) δ 171.77, 171.47, 171.33, 169.11, 163.71, 163.33, 162.82, 150.83, 147.09, 140.71, 138.88, 137.30, 134.13, 130.54, 129.15, 129.01, 128.01, 127.98, 126.79, 126.42, 122.93, 75.11, 68.24, 65.30, 64.29, 63.94, 58.07, 55.73, 55.66, 43.59, 41.02, 37.51, 37.32, 34.67, 34.57, 34.23, 27.86, 27.84, 25.76, 24.71, 24.57, 20.31, 15.32. HRMS (ESI) calcd for C_48_H_64_N_10_O_7_S [M + Na]^+^ 947.4578, found 947.4577. HPLC purity 95.55%.

#### 4.1.12 N^1^-((*S*)-1-((2*S*,4*R*)-4-Hydroxy-2-((4-(4-methylthiazol-5-yl)benzyl)carbamoyl)pyrrolidin-1-yl)-3,3-dimethyl-1-oxobutan-2-yl)-N^9^-(2-((4-(2-((E)-3-methylbenzylidene)hydrazineyl)-6-morpholinopyrimidin-2-yl)oxy)ethyl)nonanediamide (**7g**)

^1^H NMR (600 MHz, DMSO-*d*_6_) δ 10.82 (s, 1H), 8.98 (s, 1H), 8.56 (t, *J* = 6.1 Hz, 1H), 8.01 (s, 1H), 8.00 (t, *J* = 5.7 Hz, 1H), 7.83 (d, *J* = 9.4 Hz, 1H), 7.50 (d, *J* = 7.7 Hz, 1H), 7.48 (s, 1H), 7.42 (d, *J* = 8.3 Hz, 2H), 7.38 (d, *J* = 8.3 Hz, 2H), 7.29 (t, *J* = 7.6 Hz, 1H), 7.17 (d, *J* = 7.6 Hz, 1H), 6.07 (s, 1H), 5.12 (brs, 1H), 4.54 (d, *J* = 9.4 Hz, 1H), 4.46–4.40 (m, 2H), 4.37–4.33 (m, 1H), 4.22 (dd, *J* = 15.8, 5.5 Hz, 1H), 4.18 (t, *J* = 5.7 Hz, 2H), 3.70–3.63 (m, 6H), 3.53 (t, *J* = 4.9 Hz, 4H), 3.38–3.35 (m, 2H), 2.44 (s, 3H), 2.34 (s, 3H), 2.28–2.21 (m, 1H), 2.13–2.01 (m, 4H), 1.94–1.87 (m, 1H), 1.53–1.41 (m, 4H), 1.25–1.18 (m, 6H), 0.93 (s, 9H). ^13^C NMR (150 MHz, DMSO-*d*_6_) δ 170.57, 170.25, 170.10, 167.87, 162.49, 162.11, 161.59, 149.61, 145.87, 139.48, 137.66, 136.08, 132.91, 129.32, 127.93, 127.79, 126.79, 126.76, 125.57, 125.20, 121.71, 73.89, 67.01, 64.08, 63.07, 62.73, 56.84, 54.50, 54.42, 42.38, 39.80, 36.29, 36.11, 33.46, 33.35, 33.01, 26.76, 26.70, 24.53, 23.58, 23.40, 19.09, 14.10. HRMS (ESI) calcd for C_49_H_66_N_10_O_7_S [M + Na]^+^ 961.4735, found 961.4713. HPLC purity 97.86%.

#### 4.1.13 N^1^-((*S*)-1-((2*S*,4*R*)-4-Hydroxy-2-((4-(4-methylthiazol-5-yl)benzyl)carbamoyl)pyrrolidin-1-yl)-3,3-dimethyl-1-oxobutan-2-yl)-N^10^-(2-((4-(2-((E)-3-methylbenzylidene)hydrazineyl)-6-morpholinopyrimidin-2-yl)oxy)ethyl)decanediamide (**7h**)

^1^H NMR (600 MHz, DMSO-*d*_6_) δ 10.82 (s, 1H), 8.98 (s, 1H), 8.56 (t, *J* = 6.1 Hz, 1H), 8.01 (s, 1H), 8.00 (t, *J* = 5.6 Hz, 1H), 7.83 (d, *J* = 9.4 Hz, 1H), 7.50 (d, *J* = 7.7 Hz, 1H), 7.48 (s, 1H), 7.42 (d, *J* = 8.2 Hz, 2H), 7.38 (d, *J* = 8.3 Hz, 2H), 7.29 (t, *J* = 7.6 Hz, 1H), 7.17 (d, *J* = 7.5 Hz, 1H), 6.07 (s, 1H), 5.12 (brs, 1H), 4.54 (d, *J* = 9.4 Hz, 1H), 4.46–4.40 (m, 2H), 4.35 (s, 1H), 4.21 (dd, *J* = 15.9, 5.5 Hz, 1H), 4.18 (t, *J* = 5.7 Hz, 2H), 3.69–3.62 (m, 6H), 3.56–3.50 (m, 4H), 3.37–3.34 (m, 2H), 2.44 (s, 3H), 2.34 (s, 3H), 2.28–2.20 (m, 1H), 2.13–2.00 (m, 4H), 1.93–1.86 (m, 1H), 1.52–1.40 (m, 4H), 1.27–1.17 (m, 8H), 0.93 (s, 9H). ^13^C NMR (150 MHz, DMSO-*d*_6_) δ 171.77, 171.45, 171.32, 169.08, 163.70, 163.34, 162.80, 150.82, 147.08, 140.69, 138.88, 137.29, 134.12, 130.53, 129.15, 129.00, 128.00, 127.98, 126.79, 126.41, 122.92, 75.10, 68.23, 65.29, 64.29, 63.93, 58.05, 55.71, 55.63, 43.59, 41.01, 37.52, 37.32, 34.68, 34.57, 34.23, 28.13, 28.04, 25.74, 24.79, 24.64, 20.31, 15.31. HRMS (ESI) calcd for C_50_H_68_N_10_O_7_S [M + Na]^+^ 975.4891, found 975.4878. HPLC purity 98.82%.

#### 4.1.14 N^1^-((*S*)-1-((2*S*,4*R*)-4-Hydroxy-2-((4-(4-methylthiazol-5-yl)benzyl)carbamoyl)pyrrolidin-1-yl)-3,3-dimethyl-1-oxobutan-2-yl)-N^11^-(2-((4-(2-((E)-3-methylbenzylidene)hydrazineyl)-6-morpholinopyrimidin-2-yl)oxy)ethyl)undecanediamide (**7i**)

^1^H NMR (600 MHz, DMSO-*d*_6_) δ 10.82 (s, 1H), 8.98 (s, 1H), 8.56 (t, *J* = 6.1 Hz, 1H), 8.01 (s, 1H), 8.00 (t, *J* = 5.6 Hz, 1H), 7.83 (d, *J* = 9.3 Hz, 1H), 7.50 (d, *J* = 7.8 Hz, 1H), 7.48 (s, 1H), 7.42 (d, *J* = 8.3 Hz, 2H), 7.38 (d, *J* = 8.3 Hz, 2H), 7.29 (t, *J* = 7.6 Hz, 1H), 7.17 (d, *J* = 7.5 Hz, 1H), 6.07 (s, 1H), 5.12 (brs, 1H), 4.54 (d, *J* = 9.4 Hz, 1H), 4.46–4.40 (m, 2H), 4.37–4.33 (m, 1H), 4.22 (dd, *J* = 15.8, 5.5 Hz, 1H), 4.18 (t, *J* = 5.7 Hz, 2H), 3.71–3.63 (m, 6H), 3.53 (t, *J* = 4.9 Hz, 4H), 3.38–3.35 (m, 2H), 2.44 (s, 3H), 2.34 (s, 3H), 2.28–2.22 (m, 1H), 2.12–2.01 (m, 4H), 1.93–1.88 (m, 1H), 1.52–1.40 (m, 4H), 1.24–1.18 (m, 10H), 0.93 (s, 9H). ^13^C NMR (150 MHz, DMSO-*d*_6_) δ 171.79, 171.47, 171.32, 169.09, 163.71, 163.35, 162.81, 150.83, 147.09, 140.70, 138.89, 137.30, 134.13, 130.54, 129.15, 129.01, 128.01, 127.98, 126.79, 126.41, 122.93, 75.11, 68.24, 65.30, 63.95, 58.06, 55.72, 55.64, 43.60, 41.02, 37.53, 37.33, 34.69, 34.59, 34.25, 28.25, 28.18, 28.14, 28.06, 28.04, 25.75, 24.82, 24.66, 20.31, 15.32. HRMS (ESI) calcd for C_51_H_70_N_10_O_7_S [M + Na]^+^ 989.5048, found 989.5040. HPLC purity 98.36%.

#### 4.1.15 N^1^-((*S*)-1-((2*S*,4*R*)-4-Hydroxy-2-((4-(4-methylthiazol-5-yl)benzyl)carbamoyl)pyrrolidin-1-yl)-3,3-dimethyl-1-oxobutan-2-yl)-N^12^-(2-((4-(2-((E)-3-methylbenzylidene)hydrazineyl)-6-morpholinopyrimidin-2-yl)oxy)ethyl)dodecanediamide (**7j**)

^1^H NMR (600 MHz, DMSO-*d*_6_) δ 10.82 (s, 1H), 8.98 (s, 1H), 8.56 (t, *J* = 6.1 Hz, 1H), 8.01 (s, 1H), 7.99 (t, *J* = 5.7 Hz, 1H), 7.83 (d, *J* = 9.4 Hz, 1H), 7.50 (d, *J* = 7.7 Hz, 1H), 7.47 (s, 1H), 7.42 (d, *J* = 8.3 Hz, 2H), 7.38 (d, *J* = 8.3 Hz, 2H), 7.29 (t, *J* = 7.6 Hz, 1H), 7.17 (d, *J* = 7.4 Hz, 1H), 6.07 (s, 1H), 5.12 (brs, 1H), 4.54 (d, *J* = 9.4 Hz, 1H), 4.47–4.40 (m, 2H), 4.35 (s, 1H), 4.22 (dd, *J* = 15.8, 5.5 Hz, 1H), 4.18 (t, *J* = 5.7 Hz, 2H), 3.70–3.63 (m, 6H), 3.53 (t, *J* = 4.9 Hz, 4H), 3.38–3.35 (m, 2H), 2.44 (s, 3H), 2.34 (s, 3H), 2.27–2.21 (m, 1H), 2.12–2.00 (m, 4H), 1.94–1.87 (m, 1H), 1.52–1.40 (m, 4H), 1.25–1.18 (m, 12H), 0.93 (s, 9H). ^13^C NMR (150 MHz, DMSO-*d*_6_) δ 171.78, 171.46, 171.32, 169.09, 163.71, 163.35, 162.81, 150.83, 147.09, 140.69, 138.89, 137.29, 134.13, 130.54, 129.15, 129.01, 128.01, 127.98, 126.80, 126.41, 122.92, 75.11, 68.23, 65.30, 63.94, 58.06, 55.72, 55.63, 43.60, 41.02, 37.53, 37.33, 34.69, 34.59, 34.24, 28.35, 28.30, 28.20, 28.14, 28.06, 25.75, 24.82, 24.66, 20.31, 15.32. HRMS (ESI) calcd for C_52_H_72_N_10_O_7_S [M + H]^+^ 981.5384, found 981.5383. HPLC purity 99.64%.

#### 4.1.16 *tert*-Butyl (4-(2-((4-Chloro-6-morpholinopyrimidin-2-yl)oxy)ethyl)phenyl)carbamate (**8**)

Intermediate **8** was obtained according to the procedure of **3**. ^1^H NMR (500 MHz, CDCl_3_) δ 7.28 (d, *J* = 8.2 Hz, 2H), 7.19 (d, *J* = 8.2 Hz, 2H), 6.43 (s, 1H), 6.15 (s, 1H), 4.42 (t, *J* = 7.4 Hz, 2H), 3.86–3.68 (m, 4H), 3.59 (brs, 4H), 3.03 (t, *J* = 7.4 Hz, 2H), 1.51 (s, 9H).

#### 4.1.17 *tert*-Butyl (4-(2-((4-Hydrazineyl-6-morpholinopyrimidin-2-yl)oxy)ethyl)phenyl)carbamate (**9**)

Intermediate **9** was obtained according to the procedure of **4**.^1^H NMR (400 MHz, DMSO-*d*_6_) δ 9.27 (s, 1H), 7.67 (s, 1H), 7.37 (d, *J* = 8.2 Hz, 2H), 7.14 (d, *J* = 8.2 Hz, 2H), 5.61 (s, 1H), 4.63 (brs, 3H), 4.25 (t, *J* = 7.1 Hz, 2H), 3.68–3.59 (m, 4H), 3.43–3.36 (m, 4H), 2.86 (t, *J* = 7.1 Hz, 2H), 1.46 (s, 9H).

#### 4.1.18 *tert*-Butyl (E)-(4-(2-((4-(2-(3-Methylbenzylidene)hydrazineyl)-6-morpholinopyrimidin-2-yl)oxy)ethyl)phenyl)carbamate (**10**)

Intermediate **10** was obtained according to the procedure of **5**.^1^H NMR (400 MHz, DMSO-*d*_6_) δ 10.87 (s, 1H), 9.26 (s, 1H), 7.99 (s, 1H), 7.50 (d, *J* = 8.1 Hz, 2H), 7.37 (d, *J* = 8.1 Hz, 2H), 7.29 (t, *J* = 7.5 Hz, 1H), 7.20–7.10 (m, 3H), 6.06 (s, 1H), 4.32 (t, *J* = 7.0 Hz, 2H), 3.74–3.62 (m, 4H), 3.57–3.50 (m, 4H), 2.90 (t, *J* = 7.0 Hz, 2H), 2.34 (s, 3H), 1.47 (s, 9H).

#### 4.1.19 General Procedures for the Synthesis of Intermediates **11a**–**11j**

Intermediates **11a**–**11j** were obtained according to the procedure of **6a–6j**. (*E*)-3-((4-(2-((4-(2-(3-methylbenzylidene)hydrazineyl)-6-morpholinopyrimidin-2-yl)oxy)ethyl)phenyl)amino)-3-oxopropanoic acid (**11a**) ^1^H NMR (500 MHz, DMSO-*d*_6_) δ 10.99 (brs, 1H), 10.07 (s, 1H), 8.00 (s, 1H), 7.55–7.46 (m, 4H), 7.28 (t, *J* = 7.6 Hz, 1H), 7.22 (d, *J* = 8.4 Hz, 2H), 7.16 (s, 1H), 6.01 (s, 1H), 4.38 (s, 2H), 3.66 (t, *J* = 4.8 Hz, 4H), 3.54 (s, 4H), 2.95 (t, *J* = 7.0 Hz, 2H), 2.33 (s, 3H).

#### 4.1.20 General Procedures for the Synthesis of Intermediates **12a**–**12j**, **13a**, and **12dN**

Compounds **12a**–**12j**, **13a**, and **12dN** were obtained according to the procedure of **7a**–**7j**.

#### 4.1.21 N^1^-((*S*)-1-((2*S*,4*R*)-4-Hydroxy-2-((4-(4-methylthiazol-5-yl)benzyl)carbamoyl)pyrrolidin-1-yl)-3,3-dimethyl-1-oxobutan-2-yl)-N^3^-(4-(2-((4-(2-((E)-3-methylbenzylidene)hydrazineyl)-6-morpholinopyrimidin-2-yl)oxy)ethyl)phenyl)malonamide (**12a**)

^1^H NMR (600 MHz, DMSO-*d*_6_) δ 10.87 (s, 1H), 10.05 (s, 1H), 8.98 (s, 1H), 8.60 (t, *J* = 6.1 Hz, 1H), 8.23 (d, *J* = 9.3 Hz, 1H), 7.99 (s, 1H), 7.53–7.47 (m, 4H), 7.43 (d, *J* = 8.4 Hz, 2H), 7.39 (d, *J* = 8.3 Hz, 2H), 7.29 (t, *J* = 7.6 Hz, 1H), 7.23 (d, *J* = 8.5 Hz, 2H), 7.17 (d, *J* = 7.4 Hz, 1H), 6.07 (s, 1H), 5.15 (brs, 1H), 4.57 (d, *J* = 9.4 Hz, 1H), 4.49–4.41 (m, 2H), 4.38–4.32 (m, 3H), 4.23 (dd, *J* = 15.8, 5.5 Hz, 1H), 3.70–3.62 (m, 6H), 3.53 (t, *J* = 4.9 Hz, 4H), 3.43 (d, *J* = 14.9 Hz, 1H), 3.35–3.31 (m, 2H), 2.94 (t, *J* = 7.0 Hz, 2H), 2.45 (s, 3H), 2.34 (s, 3H), 2.09–2.02 (m, 1H), 1.98–1.87 (m, 1H), 0.96 (s, 9H). ^13^C NMR (150 MHz, DMSO-*d*_6_) δ 169.87, 167.29, 164.17, 163.75, 162.34, 161.91, 161.48, 149.44, 145.72, 139.22, 137.48, 135.90, 135.15, 132.74, 131.43, 129.15, 127.75, 127.65, 127.18, 126.64, 126.59, 125.42, 125.01, 121.54, 117.15, 73.64, 66.88, 64.49, 63.89, 56.73, 54.54, 54.47, 42.21, 42.12, 39.65, 35.93, 33.57, 32.25, 24.32, 24.27, 18.92, 13.93. HRMS (ESI) calcd for C_49_H_58_N_10_O_7_S [M + H]^+^ 931.4289, found 931.4286. HPLC purity 98.68%.

#### 4.1.22 N^1^-((*S*)-1-((2*S*,4*R*)-4-Hydroxy-2-((4-(4-methylthiazol-5-yl)benzyl)carbamoyl)pyrrolidin-1-yl)-3,3-dimethyl-1-oxobutan-2-yl)-N^4^-(4-(2-((4-(2-((E)-3-methylbenzylidene)hydrazineyl)-6-morpholinopyrimidin-2-yl)oxy)ethyl)phenyl)succinimide (**12b**)

^1^H NMR (600 MHz, DMSO-*d*_6_) δ 10.86 (s, 1H), 9.88 (s, 1H), 8.98 (s, 1H), 8.57 (t, *J* = 6.0 Hz, 1H), 7.99 (s, 1H), 7.96 (d, *J* = 9.3 Hz, 1H), 7.53–7.46 (m, 4H), 7.42 (d, *J* = 8.3 Hz, 2H), 7.39 (d, *J* = 8.3 Hz, 2H), 7.29 (t, *J* = 7.6 Hz, 1H), 7.20 (d, *J* = 8.5 Hz, 2H), 7.17 (d, *J* = 7.5 Hz, 1H), 6.06 (s, 1H), 5.12 (d, *J* = 3.5 Hz, 1H), 4.55 (d, *J* = 9.4 Hz, 1H), 4.47–4.40 (m, 2H), 4.33 (t, *J* = 7.1 Hz, 3H), 4.22 (dd, *J* = 15.8, 5.4 Hz, 1H), 3.72–3.60 (m, 6H), 3.55–3.49 (m, 4H), 2.92 (t, *J* = 7.0 Hz, 2H), 2.63–2.56 (m, 1H), 2.56–2.52 (m, 2H), 2.49–2.42 (m, 4H), 2.34 (s, 3H), 2.06–2.01 (m, 1H), 1.93–1.87 (m, 1H), 0.94 (s, 9H). ^13^C NMR (150 MHz, DMSO-*d*_6_) δ 172.41, 171.65, 170.78, 170.05, 164.85, 164.43, 163.99, 151.93, 148.19, 141.67, 139.98, 138.40, 138.07, 135.23, 133.37, 131.64, 130.23, 130.11, 129.56, 129.12, 129.08, 127.90, 127.49, 124.01, 119.48, 76.13, 69.36, 67.01, 66.38, 59.19, 56.93, 56.82, 44.69, 42.12, 38.40, 35.86, 34.73, 32.32, 30.59, 26.84, 21.41, 16.42. HRMS (ESI) calcd for C_50_H_60_N_10_O_7_S [M + H]^+^ 945.4445, found 945.4438. HPLC purity 99.67%.

#### 4.1.23 N^1^-((*S*)-1-((2*S*,4*R*)-4-Hydroxy-2-((4-(4-methylthiazol-5-yl)benzyl)carbamoyl)pyrrolidin-1-yl)-3,3-dimethyl-1-oxobutan-2-yl)-N^5^-(4-(2-((4-(2-((E)-3-methylbenzylidene)hydrazineyl)-6-morpholinopyrimidin-2-yl)oxy)ethyl)phenyl)glutaramide (**12c**)

^1^H NMR (600 MHz, DMSO-*d*_6_) δ 10.85 (s, 1H), 9.81 (s, 1H), 8.98 (s, 1H), 8.56 (t, *J* = 6.0 Hz, 1H), 7.99 (s, 1H), 7.92 (d, *J* = 9.2 Hz, 1H), 7.54–7.45 (m, 4H), 7.42 (d, *J* = 8.3 Hz, 2H), 7.40–7.36 (m, 2H), 7.29 (t, *J* = 7.6 Hz, 1H), 7.20 (d, *J* = 8.5 Hz, 2H), 7.17 (d, *J* = 7.5 Hz, 1H), 6.06 (s, 1H), 5.14 (d, *J* = 3.3 Hz, 1H), 4.55 (d, *J* = 9.3 Hz, 1H), 4.46–4.40 (m, 2H), 4.38–4.32 (m, 3H), 4.22 (dd, *J* = 15.8, 5.4 Hz, 1H), 3.70–3.62 (m, 6H), 3.55–3.50 (m, 4H), 2.92 (t, *J* = 6.9 Hz, 2H), 2.44 (s, 3H), 2.34 (s, 3H), 2.32–2.25 (m, 3H), 2.24–2.18 (m, 1H), 2.07–2.00 (m, 1H), 1.95–1.87 (m, 1H), 1.84–1.75 (m, 2H), 0.95 (s, 9H). ^13^C NMR (150 MHz, DMSO-*d*_6_) δ 172.41, 172.18, 171.20, 170.20, 164.84, 164.43, 163.99, 151.92, 148.19, 141.68, 139.98, 138.39, 138.05, 135.23, 133.45, 131.64, 130.23, 130.11, 129.53, 129.11, 129.08, 127.89, 127.49, 124.02, 119.63, 76.13, 69.37, 67.01, 66.38, 59.18, 56.91, 56.84, 44.68, 42.12, 38.42, 36.32, 35.66, 34.74, 34.68, 26.88, 21.99, 21.41, 16.42. HRMS (ESI) calcd for C_51_H_62_N_10_O_7_S [M + H]^+^ 959.4602, found 959.4584. HPLC purity 99.28%.

#### 4.1.24 N^1^-((*S*)-1-((2*S*,4*R*)-4-Hydroxy-2-((4-(4-methylthiazol-5-yl)benzyl)carbamoyl)pyrrolidin-1-yl)-3,3-dimethyl-1-oxobutan-2-yl)-N^6^-(4-(2-((4-(2-((E)-3-methylbenzylidene)hydrazineyl)-6-morpholinopyrimidin-2-yl)oxy)ethyl)phenyl)adipamide (**12d**)

^1^H NMR (600 MHz, DMSO-*d*_6_) δ 10.86 (s, 1H), 9.81 (s, 1H), 8.98 (s, 1H), 8.57 (t, *J* = 6.1 Hz, 1H), 7.99 (s, 1H), 7.88 (d, *J* = 9.3 Hz, 1H), 7.53–7.47 (m, 4H), 7.42 (d, *J* = 8.3 Hz, 2H), 7.40–7.36 (m, 2H), 7.29 (t, *J* = 7.6 Hz, 1H), 7.20 (d, *J* = 8.5 Hz, 2H), 7.17 (d, *J* = 7.5 Hz, 1H), 6.07 (s, 1H), 5.13 (d, *J* = 3.6 Hz, 1H), 4.55 (d, *J* = 9.4 Hz, 1H), 4.47–4.41 (m, 2H), 4.33 (t, *J* = 7.1 Hz, 3H), 4.22 (dd, *J* = 15.9, 5.5 Hz, 1H), 3.71–3.62 (m, 6H), 3.56–3.50 (m, 4H), 2.92 (t, *J* = 7.0 Hz, 2H), 2.45 (s, 3H), 2.34 (s, 3H), 2.32–2.24 (m, 3H), 2.20–2.13 (m, 1H), 2.06–2.00 (m, 1H), 1.94–1.87 (m, 1H), 1.61–1.48 (m, 4H), 0.94 (s, 9H). ^13^C NMR (150 MHz, DMSO-*d*_6_) δ 172.42, 172.41, 171.45, 170.19, 164.85, 164.44, 163.99, 151.92, 148.19, 141.68, 139.98, 138.39, 138.04, 135.24, 133.46, 131.64, 130.23, 130.11, 129.55, 129.11, 129.08, 127.89, 127.49, 124.02, 119.60, 76.14, 69.35, 67.01, 66.38, 59.17, 56.85, 56.81, 44.69, 42.12, 38.42, 36.69, 35.69, 35.21, 34.74, 26.93, 26.87, 25.67, 25.44, 21.41, 16.42. HRMS (ESI) calcd for C_52_H_64_N_10_O_7_S [M + H]^+^ 973.4758, found 973.4757. HPLC purity 99.00%.

#### 4.1.25 N^1^-((*S*)-1-((2*S*,4*R*)-4-Hydroxy-2-((4-(4-methylthiazol-5-yl)benzyl)carbamoyl)pyrrolidin-1-yl)-3,3-dimethyl-1-oxobutan-2-yl)-N^7^-(4-(2-((4-(2-((E)-3-methylbenzylidene)hydrazineyl)-6-morpholinopyrimidin-2-yl)oxy)ethyl)phenyl)heptanediamide (**12e**)

^1^H NMR (600 MHz, DMSO-*d*_6_) δ 10.85 (s, 1H), 9.79 (s, 1H), 8.98 (d, *J* = 5.4 Hz, 1H), 8.56 (t, *J* = 6.1 Hz, 1H), 7.99 (s, 1H), 7.86 (d, *J* = 9.3 Hz, 1H), 7.53–7.46 (m, 4H), 7.42 (d, *J* = 8.3 Hz, 2H), 7.38 (d, *J* = 8.3 Hz, 2H), 7.29 (t, *J* = 7.6 Hz, 1H), 7.20 (d, *J* = 8.5 Hz, 2H), 7.17 (d, *J* = 7.5 Hz, 1H), 6.06 (s, 1H), 5.13 (d, *J* = 3.6 Hz, 1H), 4.54 (d, *J* = 9.4 Hz, 1H), 4.45–4.40 (m, 2H), 4.37–4.30 (m, 3H), 4.22 (dd, *J* = 15.8, 5.5 Hz, 1H), 3.70–3.62 (m, 6H), 3.55–3.50 (m, 4H), 2.92 (t, *J* = 7.0 Hz, 2H), 2.44 (s, 3H), 2.34 (s, 3H), 2.30–2.22 (m, 3H), 2.16–2.10 (m, 1H), 2.07–2.00 (m, 1H), 1.95–1.86 (m, 1H), 1.62–1.43 (m, 4H), 1.32–1.23 (m, 2H), 0.93 (s, 9H). ^13^C NMR (150 MHz, DMSO-*d*_6_) δ 172.51, 172.42, 171.51, 170.19, 164.84, 164.43, 163.99, 151.93, 148.19, 141.68, 139.98, 138.39, 138.08, 135.23, 133.41, 131.64, 130.23, 130.11, 129.54, 129.11, 129.08, 127.89, 127.49, 124.01, 119.58, 76.13, 69.34, 67.01, 66.38, 59.16, 56.84, 56.76, 44.68, 42.12, 38.43, 36.75, 35.68, 35.26, 34.74, 28.84, 26.86, 25.73, 25.41, 21.41, 16.42. HRMS (ESI) calcd for C_53_H_66_N_10_O_7_S [M + H]^+^ 987.4915, found 987.4912. HPLC purity 99.85%.

#### 4.1.26 N^1^-((*S*)-1-((2*S*,4*R*)-4-Hydroxy-2-((4-(4-methylthiazol-5-yl)benzyl)carbamoyl)pyrrolidin-1-yl)-3,3-dimethyl-1-oxobutan-2-yl)-N^8^-(4-(2-((4-(2-((E)-3-methylbenzylidene)hydrazineyl)-6-morpholinopyrimidin-2-yl)oxy)ethyl)phenyl)octanediamide (**12f**)

^1^H NMR (600 MHz, DMSO-*d*_6_) δ 10.85 (s, 1H), 9.80 (s, 1H), 8.98 (s, 1H), 8.56 (t, *J* = 6.1 Hz, 1H), 7.99 (s, 1H), 7.85 (d, *J* = 9.4 Hz, 1H), 7.53–7.46 (m, 4H), 7.42 (d, *J* = 8.2 Hz, 2H), 7.40–7.36 (m, 2H), 7.29 (t, *J* = 7.6 Hz, 1H), 7.20 (d, *J* = 8.5 Hz, 2H), 7.17 (d, *J* = 7.5 Hz, 1H), 6.06 (s, 1H), 5.13 (d, *J* = 3.6 Hz, 1H), 4.55 (d, *J* = 9.4 Hz, 1H), 4.46–4.40 (m, 2H), 4.37–4.31 (m, 3H), 4.22 (dd, *J* = 15.9, 5.5 Hz, 1H), 3.70–3.63 (m, 6H), 3.55–3.50 (m, 4H), 2.92 (t, *J* = 7.0 Hz, 2H), 2.44 (s, 3H), 2.34 (s, 3H), 2.29–2.22 (m, 3H), 2.15–2.10 (m, 1H), 2.06–2.01 (m, 1H), 1.93–1.88 (m, 1H), 1.60–1.42 (m, 4H), 1.32–1.24 (m, 4H), 0.94 (s, 9H). ^13^C NMR (150 MHz, DMSO-*d*_6_) δ 172.56, 172.43, 171.55, 170.20, 164.84, 164.43, 163.99, 151.92, 148.19, 141.68, 139.98, 138.39, 138.07, 135.23, 133.43, 131.64, 130.23, 130.11, 129.54, 129.11, 129.08, 127.89, 127.49, 124.02, 119.59, 76.13, 69.34, 67.01, 66.38, 59.16, 56.84, 56.76, 44.68, 42.12, 38.42, 36.84, 35.68, 35.34, 34.74, 28.98, 28.94, 26.86, 25.83, 25.56, 21.41, 16.42. HRMS (ESI) calcd for C_54_H_68_N_10_O_7_S [M + H]^+^ 1001.5071, found 1001.5063. HPLC purity 99.74%.

#### 4.1.27 N^1^-((*S*)-1-((2*S*,4*R*)-4-Hydroxy-2-((4-(4-methylthiazol-5-yl)benzyl)carbamoyl)pyrrolidin-1-yl)-3,3-dimethyl-1-oxobutan-2-yl)-N^9^-(4-(2-((4-(2-((E)-3-methylbenzylidene)hydrazineyl)-6-morpholinopyrimidin-2-yl)oxy)ethyl)phenyl)nonanediamide (**12g**)

^1^H NMR (600 MHz, DMSO-*d*_6_) δ 10.85 (s, 1H), 9.79 (s, 1H), 8.98 (s, 1H), 8.56 (t, *J* = 6.1 Hz, 1H), 7.99 (s, 1H), 7.84 (d, *J* = 9.4 Hz, 1H), 7.53–7.46 (m, 4H), 7.42 (d, *J* = 8.3 Hz, 2H), 7.40–7.36 (m, 2H), 7.29 (t, *J* = 7.6 Hz, 1H), 7.20 (d, *J* = 8.5 Hz, 2H), 7.17 (d, *J* = 7.5 Hz, 1H), 6.06 (s, 1H), 5.12 (d, *J* = 3.6 Hz, 1H), 4.54 (d, *J* = 9.4 Hz, 1H), 4.46–4.39 (m, 2H), 4.33 (t, *J* = 7.1 Hz, 3H), 4.21 (dd, *J* = 15.8, 5.5 Hz, 1H), 3.72–3.61 (m, 6H), 3.56–3.48 (m, 4H), 2.92 (t, *J* = 7.0 Hz, 2H), 2.44 (s, 3H), 2.34 (s, 3H), 2.30–2.20 (m, 3H), 2.15–2.08 (m, 1H), 2.05–1.98 (m, 1H), 1.95–1.85 (m, 1H), 1.62–1.40 (m, 4H), 1.30–1.20 (m, 6H), 0.93 (s, 9H). ^13^C NMR (150 MHz, DMSO-*d*_6_) δ 170.70, 170.56, 169.71, 168.32, 162.98, 162.57, 162.13, 150.06, 146.33, 139.81, 138.12, 136.53, 136.21, 133.37, 131.56, 129.77, 128.37, 128.24, 127.68, 127.25, 127.22, 126.03, 125.63, 122.15, 117.72, 74.27, 67.47, 65.14, 64.52, 57.29, 54.96, 54.87, 42.82, 38.67, 36.56, 34.99, 33.81, 33.46, 32.87, 27.21, 27.16, 24.98, 24.03, 23.76, 19.55, 14.56. HRMS (ESI) calcd for C_55_H_70_N_10_O_7_S [M + H]^+^ 1015.5228, found 1015.5218. HPLC purity 98.87%.

#### 4.1.28 N^1^-((*S*)-1-((2*S*,4*R*)-4-Hydroxy-2-((4-(4-methylthiazol-5-yl)benzyl)carbamoyl)pyrrolidin-1-yl)-3,3-dimethyl-1-oxobutan-2-yl)-N^10^-(4-(2-((4-(2-((E)-3-methylbenzylidene)hydrazineyl)-6-morpholinopyrimidin-2-yl)oxy)ethyl)phenyl)decanediamide (**12h**)

^1^H NMR (600 MHz, DMSO-*d*_6_) δ 10.85 (s, 1H), 9.79 (s, 1H), 8.98 (s, 1H), 8.56 (t, *J* = 6.0 Hz, 1H), 7.99 (s, 1H), 7.84 (d, *J* = 9.4 Hz, 1H), 7.54–7.46 (m, 4H), 7.42 (d, *J* = 8.3 Hz, 2H), 7.38 (d, *J* = 8.3 Hz, 2H), 7.29 (t, *J* = 7.6 Hz, 1H), 7.20 (d, *J* = 8.5 Hz, 2H), 7.17 (d, *J* = 7.5 Hz, 1H), 6.06 (s, 1H), 5.12 (d, *J* = 3.6 Hz, 1H), 4.54 (d, *J* = 9.4 Hz, 1H), 4.46–4.40 (m, 2H), 4.37–4.31 (m, 3H), 4.22 (dd, *J* = 15.8, 5.4 Hz, 1H), 3.70–3.62 (m, 6H), 3.56–3.50 (m, 4H), 2.92 (t, *J* = 7.0 Hz, 2H), 2.44 (s, 3H), 2.34 (s, 3H), 2.29–2.23 (m, 3H), 2.14–2.08 (m, 1H), 2.05–1.99 (m, 1H), 1.94–1.87 (m, 1H), 1.60–1.42 (m, 4H), 1.32–1.17 (m, 8H), 0.93 (s, 9H). ^13^C NMR (150 MHz, DMSO-*d*_6_) δ 172.56, 172.42, 171.56, 170.19, 164.84, 164.43, 163.99, 151.92, 148.19, 141.67, 139.98, 138.39, 138.08, 135.24, 133.41, 131.64, 130.23, 130.11, 129.54, 129.11, 129.08, 127.89, 127.49, 124.01, 119.57, 76.13, 69.33, 67.01, 66.38, 59.16, 56.82, 56.74, 44.68, 42.11, 38.43, 36.86, 35.68, 35.32, 34.74, 29.24, 29.15, 29.13, 26.85, 25.89, 25.63, 21.41, 16.42. HRMS (ESI) calcd for C_56_H_72_N_10_O_7_S [M + H]^+^ 1029.5384, found 1029.5378. HPLC purity 99.35%.

#### 4.1.29 N^1^-((*S*)-1-((2*S*,4*R*)-4-Hydroxy-2-((4-(4-methylthiazol-5-yl)benzyl)carbamoyl)pyrrolidin-1-yl)-3,3-dimethyl-1-oxobutan-2-yl)-N^11^-(4-(2-((4-(2-((E)-3-methylbenzylidene)hydrazineyl)-6-morpholinopyrimidin-2-yl)oxy)ethyl)phenyl)undecanediamide (**12i**)

^1^H NMR (600 MHz, DMSO-*d*_6_) δ 10.85 (s, 1H), 9.79 (s, 1H), 8.98 (s, 1H), 8.55 (t, *J* = 6.1 Hz, 1H), 7.99 (s, 1H), 7.84 (d, *J* = 9.4 Hz, 1H), 7.52–7.47 (m, 4H), 7.42 (d, *J* = 8.3 Hz, 2H), 7.39–7.37 (m, 2H), 7.29 (t, *J* = 7.6 Hz, 1H), 7.20 (d, *J* = 8.5 Hz, 2H), 7.17 (d, *J* = 7.5 Hz, 1H), 6.06 (s, 1H), 5.11 (d, *J* = 3.2 Hz, 1H), 4.54 (d, *J* = 9.4 Hz, 1H), 4.45–4.40 (m, 2H), 4.33 (t, *J* = 7.1 Hz, 3H), 4.21 (dd, *J* = 15.9, 5.4 Hz, 1H), 3.68–3.62 (m, 6H), 3.54–3.50 (m, 4H), 2.92 (t, *J* = 7.0 Hz, 2H), 2.44 (s, 3H), 2.34 (s, 3H), 2.29–2.22 (m, 3H), 2.14–2.07 (m, 1H), 2.05–1.97 (m, 1H), 1.93–1.86 (m, 1H), 1.62–1.52 (m, 2H), 1.53–1.42 (m, 2H), 1.30–1.20 (m, 12H), 0.93 (s, 9H). ^13^C NMR (150 MHz, DMSO-*d*_6_) δ 172.56, 172.42, 171.56, 170.18, 164.84, 164.43, 163.99, 151.93, 148.19, 141.67, 139.98, 138.39, 138.08, 135.23, 133.42, 131.64, 130.23, 130.11, 129.54, 129.11, 129.08, 127.89, 127.49, 124.01, 119.58, 76.13, 69.33, 67.01, 66.38, 59.15, 56.82, 56.73, 44.68, 42.11, 38.43, 36.86, 35.68, 35.33, 34.74, 29.34, 29.27, 29.22, 29.16, 29.13, 26.85, 25.91, 25.64, 21.41, 16.42. HRMS (ESI) calcd for C_57_H_74_N_10_O_7_S [M + H]^+^ 1043.5541, found 1043.5527. HPLC purity 96.81%.

#### 4.1.30 N^1^-((*S*)-1-((2*S*,4*R*)-4-Hydroxy-2-((4-(4-methylthiazol-5-yl)benzyl)carbamoyl)pyrrolidin-1-yl)-3,3-dimethyl-1-oxobutan-2-yl)-N^12^-(4-(2-((4-(2-((E)-3-methylbenzylidene)hydrazineyl)-6-morpholinopyrimidin-2-yl)oxy)ethyl)phenyl)dodecanediamide (**12j**)

^1^H NMR (600 MHz, DMSO-*d*_6_) δ 10.86 (s, 1H), 9.79 (s, 1H), 8.98 (s, 1H), 8.56 (t, *J* = 6.1 Hz, 1H), 7.99 (d, *J* = 1.0 Hz, 1H), 7.84 (d, *J* = 9.4 Hz, 1H), 7.52–7.47 (m, 4H), 7.42 (d, *J* = 8.3 Hz, 2H), 7.38 (d, *J* = 8.3 Hz, 2H), 7.29 (t, *J* = 7.6 Hz, 1H), 7.20 (d, *J* = 8.5 Hz, 2H), 7.17 (d, *J* = 7.6 Hz, 1H), 6.06 (s, 1H), 5.12 (brs, 1H), 4.54 (d, *J* = 9.4 Hz, 1H), 4.46–4.40 (m, 2H), 4.38–4.30 (m, 3H), 4.22 (dd, *J* = 15.8, 5.5 Hz, 1H), 3.68–3.63 (m, 6H), 3.53 (t, *J* = 4.9 Hz, 4H), 2.92 (t, *J* = 7.1 Hz, 2H), 2.44 (s, 3H), 2.34 (s, 3H), 2.29–2.22 (m, 3H), 2.13–2.07 (m, 1H), 2.06–2.00 (m, 1H), 1.94–1.87 (m, 1H), 1.57 (t, *J* = 7.2 Hz, 2H), 1.53–1.40 (m, 2H), 1.30–1.22 (m, 12H), 0.93 (s, 9H). ^13^C NMR (150 MHz, DMSO-*d*_6_) δ 171.47, 171.32, 170.47, 169.09, 163.73, 163.31, 162.87, 150.82, 147.09, 140.60, 138.89, 137.29, 136.98, 134.13, 132.31, 130.54, 129.14, 129.01, 128.44, 128.01, 127.98, 126.79, 126.40, 122.92, 118.48, 75.03, 68.24, 65.93, 65.28, 58.06, 55.72, 55.64, 43.59, 41.02, 37.33, 35.75, 34.59, 34.24, 33.64, 28.33, 28.29, 28.18, 28.12, 28.08, 28.04, 25.75, 24.82, 24.53, 20.31, 15.32. HRMS (ESI) calcd for C_58_H_76_N_10_O_7_S [M + H]^+^ 1057.5697, found 1057.5696. HPLC purity 98.53%.

#### 4.1.31 N^1^-((*S*)-1-((2*S*,4*R*)-4-Hydroxy-2-(((*S*)-1-(4-(4-methylthiazol-5-yl)phenyl)ethyl)carbamoyl)pyrrolidin-1-yl)-3,3-dimethyl-1-oxobutan-2-yl)-N^6^-(4-(2-((4-(2-((E)-3-methylbenzylidene)hydrazineyl)-6-morpholinopyrimidin-2-yl)oxy)ethyl)phenyl)adipamide (**13a**)

^1^H NMR (600 MHz, DMSO-*d*_6_) δ 10.86 (s, 1H), 9.81 (s, 1H), 8.98 (s, 1H), 8.37 (d, *J* = 7.8 Hz, 1H), 7.99 (s, 1H), 7.81 (d, *J* = 9.2 Hz, 1H), 7.53–7.46 (m, 4H), 7.43 (d, *J* = 8.2 Hz, 2H), 7.37 (d, *J* = 8.2 Hz, 2H), 7.29 (t, *J* = 7.6 Hz, 1H), 7.20 (d, *J* = 8.5 Hz, 2H), 7.17 (d, *J* = 8.0 Hz, 1H), 6.06 (s, 1H), 5.10 (s, 1H), 4.96–4.88 (m, 1H), 4.51 (d, *J* = 9.3 Hz, 1H), 4.43 (t, *J* = 8.0 Hz, 1H), 4.33 (t, *J* = 7.0 Hz, 2H), 4.28 (s, 1H), 3.69–3.64 (m, 4H), 3.64–3.58 (m, 2H), 3.56–3.49 (m, 4H), 2.93 (t, *J* = 7.0 Hz, 2H), 2.45 (s, 3H), 2.34 (s, 3H), 2.31–2.25 (m, 3H), 2.19–2.12 (m, 1H), 2.04–1.97 (m, 1H), 1.83–1.75 (m, 1H), 1.60–1.48 (m, 4H), 1.37 (d, *J* = 7.0 Hz, 3H), 0.94 (s, 9H). ^13^C NMR (150 MHz, DMSO-*d*_6_) δ 171.26, 170.35, 169.99, 168.98, 163.73, 163.31, 162.87, 150.85, 147.12, 144.03, 140.59, 137.29, 136.94, 134.13, 132.35, 130.48, 129.13, 129.06, 128.46, 128.19, 127.98, 126.40, 125.74, 125.61, 122.92, 118.50, 75.02, 68.14, 65.92, 65.28, 57.92, 55.78, 55.64, 47.06, 43.59, 37.08, 35.59, 34.56, 34.13, 33.64, 25.83, 24.55, 24.32, 21.81, 20.31, 15.36. HRMS (ESI) calcd for C_53_H_66_N_10_O_7_S [M + Na]^+^ 1009.4735, found 1009.4711. HPLC purity 97.91%.

#### 4.1.32 N^1^-((*S*)-1-((2*S*,4*S*)-4-Hydroxy-2-((4-(4-methylthiazol-5-yl)benzyl)carbamoyl)pyrrolidin-1-yl)-3,3-dimethyl-1-oxobutan-2-yl)-N^6^-(4-(2-((4-(2-((E)-3-methylbenzylidene)hydrazineyl)-6-morpholinopyrimidin-2-yl)oxy)ethyl)phenyl)adipamide (**12dN**)

^1^H NMR (600 MHz, DMSO-*d*_6_) δ 10.85 (s, 1H), 9.80 (s, 1H), 8.98 (s, 1H), 8.63 (t, *J* = 6.1 Hz, 1H), 7.99 (s, 1H), 7.87 (d, *J* = 8.8 Hz, 1H), 7.50 (t, *J* = 8.2 Hz, 3H), 7.47 (s, 1H), 7.42–7.37 (m, 4H), 7.29 (t, *J* = 7.6 Hz, 1H), 7.20 (d, *J* = 8.5 Hz, 2H), 7.17 (d, *J* = 7.4 Hz, 1H), 6.06 (s, 1H), 5.43 (d, *J* = 7.3 Hz, 1H), 4.49–4.41 (m, 2H), 4.39–4.31 (m, 3H), 4.26 (dd, *J* = 15.8, 5.5 Hz, 1H), 4.23–4.18 (m, 1H), 3.94 (dd, *J* = 10.0, 5.7 Hz, 1H), 3.69–3.64 (m, 4H), 3.55–3.51 (m, 4H), 3.44 (dd, *J* = 10.0, 5.3 Hz, 1H), 2.92 (t, *J* = 7.0 Hz, 2H), 2.44 (s, 3H), 2.34 (s, 4H), 2.33–2.22 (m, 4H), 2.19–2.11 (m, 1H), 1.77–1.71 (m, 1H), 1.58–1.47 (m, 4H), 0.95 (s, 9H). ^13^C NMR (150 MHz, DMSO-*d*_6_) δ 172.96, 172.74, 171.44, 170.46, 164.85, 164.44, 164.00, 148.22, 141.73, 139.68, 138.40, 138.04, 135.24, 133.47, 131.61, 130.20, 129.64, 129.49, 129.24, 129.16, 129.04, 128.02, 127.92, 127.82, 119.64, 76.14, 69.57, 67.01, 66.39, 65.40, 58.99, 57.16, 56.09, 44.69, 42.26, 37.40, 36.70, 35.13, 35.10, 34.74, 26.86, 25.64, 25.40, 21.42, 16.42. HRMS (ESI) calcd for C_52_H_64_N_10_O_7_S [M + Na]^+^ 995.4578, found 995.4571. HPLC purity 95.44%.

### 4.2 Molecular Docking

The only available protein structure of PIKfyve was the crystal complex of PIKfyve, Figure 4, and Vac14 (PDB:7K2V) to date. Figure 4 and Vac14 were removed before protein preparation, which was operated by assigning bond orders and adding hydrogens in the Protein Preparation Wizard section of Maestro Version 11.9 (Schrodinger, LLC, New York, 2019). The chemical structure of apilimod was constructed and prepared by using the LigPrep section with the default settings and the OPLS3e force field. The grid box of the receptor was generated as the center of residue Leu119. Molecular docking was operated in extra precision by using the Glide section of Maestro. The final images were prepared by Pymol (http://pymol.org).

### 4.3 Cell Line

Human prostate cancer cell lines VCaP, PC3, 22RV1, and LNCaP were purchased from American Type Culture Collection (ATCC) and maintained under 5% CO_2_ at 37 °C in a medium according to ATCC’s instruction. All cell lines were tested negative for mycoplasma and authenticated by genotyping.

### 4.4 Western Blot Analysis

The whole cell lysate was harvested in Pierce radioimmunoprecipitation assay (RIPA) buffer (ThermoScientific) containing protease and phosphatase inhibitor cocktails. Protein concentration was measured using the detergent compatible (DC) protein assay (Bio-Rad). Denatured lysates were separated on NuPage 4–12% Bis-Tris Midi Protein gels (Novex) and transferred to 0.45 μm polyvinylidene difluoride membrane (Immobilon) using a TransBlot Turbo dry transfer machine (Bio-Rad). The membrane was incubated in blocking buffer (5% non-fat dry milk, Tris-buffered saline with 0.1% Tween-20) for 1 h at room temperature. The membrane was then incubated with primary antibody for 1 h at room temperature, followed by overnight incubation at 4 °C. Chemiluminescent detection using ECL Prime (Amersham) and signal were visualized by an Odyssey imaging system (Li-Cor). Primary antibodies were PIKfyve (R&D, AF7885), LC3A/B (CST, 12741S), GAPDH (CST, 3683S), and vinculin (CST, 18799S). All antibodies were used at dilutions suggested by the manufacturers.

### 4.5 TMT-Labeled Quantitative Proteomic Analysis

VCaP cells were plated at 3 × 10^6^ cells per well in a 6-well plate overnight prior to treatment with DMSO or 300 nM **PIK5-12d** for 4 h. Whole cell lysates were collected in RIPA buffer (Thermo Fisher Scientific) without protease inhibitor. Total protein (75 μg) per condition was labeled with TMT isobaric Label Reagent (Thermo Fisher Scientific) according to the manufacturer’s protocol and subjected to 12 fractions of liquid chromatography–mass spectrometry (LC–MS)/MS analysis.

### 4.6 In Vivo Experiment

All in vivo experiments were approved by the University of Michigan Institutional Animal Care and Use Committee. LTL-331R tumor was kindly provided by Dr. Yuzhuo Wang’s group in Vancouver Prostate Centre and maintained subcutaneously on both sides of dorsal flanks of male CB17 severe combined immunodeficiency mice. **PIK5-12d** was freshly dissolved in the vehicle (5% DMSO and 95% of 40% hydroxypropyl-β-cyclodextrin) for once-daily IP injection. Pharmacodynamic assessment was performed by once-daily administration of either vehicle, 4 or 10 mg/kg **PIK5-12d** for 3 days, and tumor samples were collected 24 h post last dose on day 4 for protein and TUNEL in situ cell death assay assessment. Long-term tumor efficacy of the LTL-331R model was determined by once-daily administration of **PIK5-12d** at 5 days on and 2 days off regimen for 17 days. Tumors were measured at least twice per week using digital calipers following the formula (*p*/6) (*L* × *W*^2^), where *L* and *W* are the length and width of the tumors, respectively.

### 4.7 TUNEL Assay

Tumor tissue was fixed in formalin and embedded into paraffin. Formalin-fixed, paraffin-embedded tissue was sectioned into 5 μm thickness and then deparaffined and rehydrated by xylene and ethanol gradients. TUNEL signal was stained using In Situ Cell Death Detection Kit TMR (Roche) according to manufacturer’s instruction. TUNEL signal was visualized by a Zeiss fluorescence microscope.

## Supporting Information Available

^1^H NMR and ^13^C NMR spectra, and HPLC traces for all degraders (PDF)
Molecular formula strings (CSV)
Docking pose of compound **1** in PIKfyve (PDB)
The data set of TMT proteomics (XLSX)

## Supplementary Material

jm3c00912_si_002.csv

jm3c00912_si_003.pdb

## Abbreviations

AcOH: acetic acid
ATCC: American type culture collection
CH_2_Cl_2_: dichloromethane
DC_50_: the half-maximal degradation concentration
*D*_max_: the maximal degradation rate
DMF: *N*,*N*-dimethylformamide
DMSO: dimethyl sulfoxide
EtOH: ethyl alcohol
Et_3_N: triethylamine
HATU: 2-(7-azabenzotriazol-1-yl)-*N*,*N*,*N′*,*N′*-tetramethyluronium hexafluorophosphate
HRMS: high-resolution mass
IP: intraperitoneal
NaH: sodium hydride
PDX: patient-derived xenograft
PI(3)P: phosphatidylinositol-3-phosphate
PI(3,5)P_2_: phosphatidylinositol-3,5-bisphosphate
POI: protein-of-interest
PROTAC: proteolysis-targeting chimera
SAR: structure–activity relationship
SDR: structure–degradation relationship
TFA: trifluoroacetic acid
TLC: thin-layer chromatography
TMT: tandem mass tags
UPS: ubiquitin-proteasome system
VHL: von Hippel–Lindau
