# Supplementary material for: Discovery of a First-in-Class Degrader for the Lipid Kinase PIKfyve
Source: J Med Chem. 2023 Aug 21;66(17):12432–45. doi: 10.1021/acs.jmedchem.3c00912 (PMC10510382; doi:10.1021/acs.jmedchem.3c00912)
Supplement: Supplementary file 1 — jm3c00912_si_001.pdf [file jm3c00912_si_001.pdf]

Supporting Information

Discovery of a First-in-Class Degrader for the Lipid Kinase PIKfyve

Chungen Li[1,#], Yuanyuan Qiao[2,4,#], Xia Jiang[2], Lianchao Liu[1], Yang Zheng[2], Yudi Qiu[1], Caleb Cheng[2], Fengtao Zhou[8], Yang Zhou[8], Weixue Huang[1], Xiaomei Ren[1], Yuzhuo Wang[3], Zhen Wang[1,*], Arul M. Chinnaiyan[2,4,5,6,*], Ke Ding[1,7,8,*]

[1] State Key Laboratory of Chemical Biology, Shanghai Institute of Organic Chemistry, Chinese Academy of Sciences, #345 Lingling Rd., Shanghai 200032, People's Republic of China

[2] Michigan Center for Translational Pathology, University of Michigan, Ann Arbor, MI 48109, USA.

[3] The Vancouver Prostate Centre, Vancouver General Hospital and Department of Urologic Sciences, The University of British Columbia, Vancouver, V6H 3Z6 BC, Canada.

[4] Department of Pathology, University of Michigan, Ann Arbor, MI 48109, USA.

[5] Howard Hughes Medical Institute, University of Michigan, Ann Arbor, MI 48109, USA.

[6] Department of Urology, University of Michigan, Ann Arbor, MI 48109, USA.

[7] Institute of Basic Medicine and Cancer (IBMC), Chinese Academy of Sciences, Hangzhou, Zhejiang 310022, People's Republic of China

[8] International Cooperative Laboratory of Traditional Chinese Medicine Modernization and Innovative Drug Discovery of Chinese Ministry of Education

(MOE), Guangzhou City Key Laboratory of Precision Chemical Drug Development, College of Pharmacy, Jinan University, 855 Xingye Avenue East, Guangzhou 511400, People's Republic of China

#C. L. and Y. Q. contributed equally to this work.

*Email: wangz@sioc.ac.cn (Z.W.); arul@med.umich.edu (A.M.C); dingk@sioc.ac.cn; Tel: +86-21-5492 5100 (K.D.)

Table of Contents

1. NMR spectra and HPLC traces of final compounds…..………………...S3-S46

NMR spectra and HPLC traces of final compounds

Compound **7a**

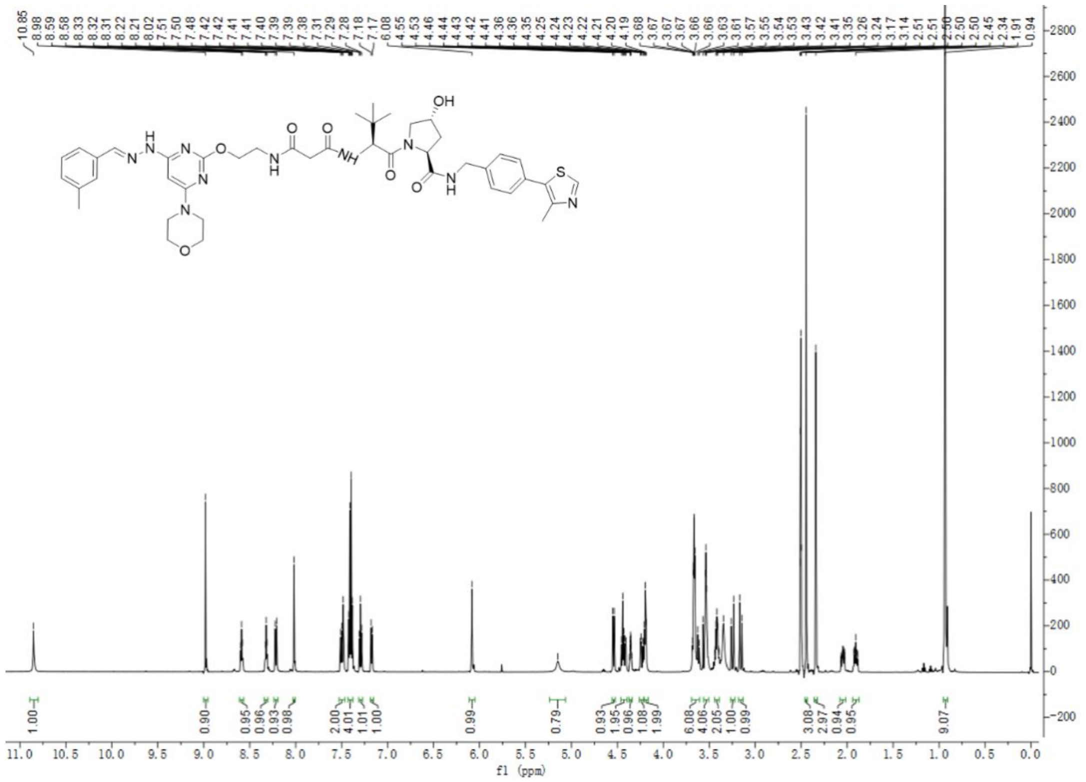

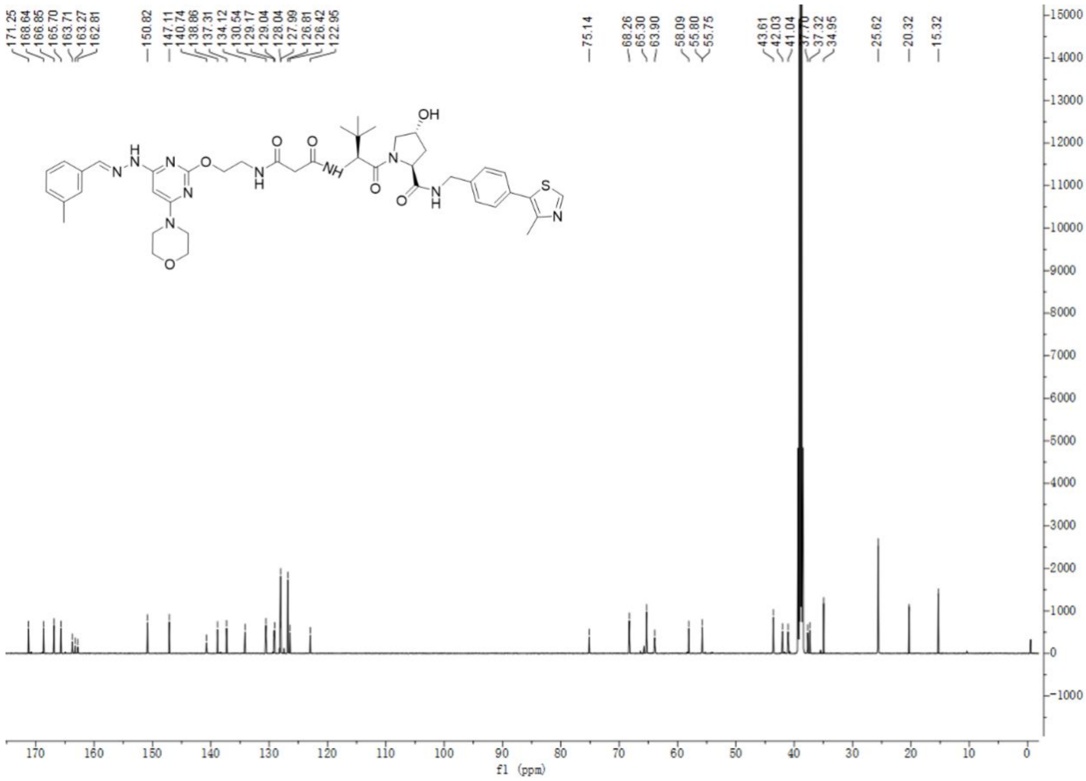

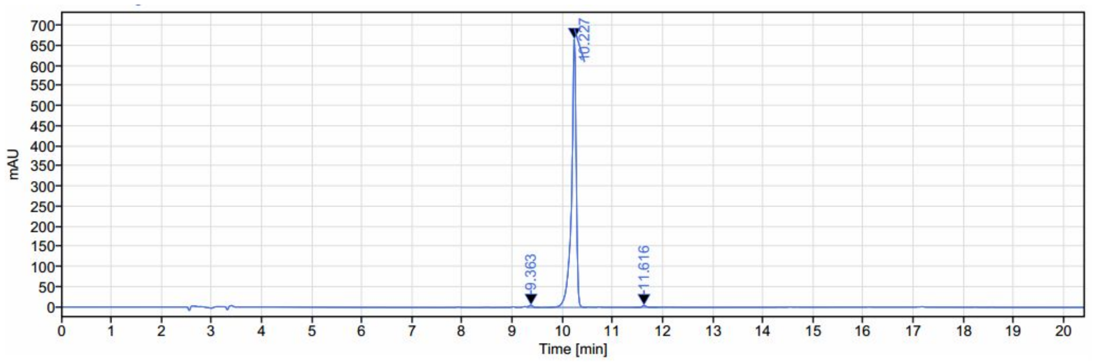

Signal: DAD1A,Sig=254,4 Ref=off

RT [min]	Type	Width [min]	Area	Height	Area%	Name
9.363	VB	0.26	31.90	5.51	0.73	
10.227	VV	0.77	4330.01	667.71	98.63	
11.616	BV	0.47	28.35	4.77	0.65	
		**Sum**	4390.27		100.00	

Compound **7b**

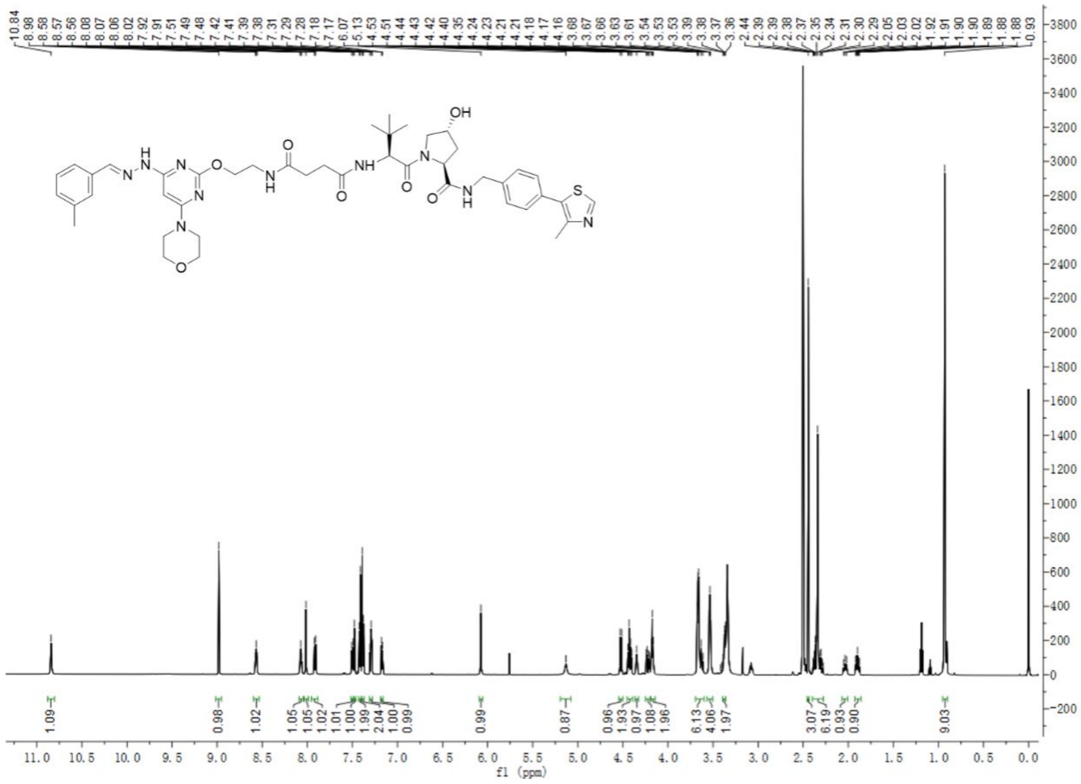

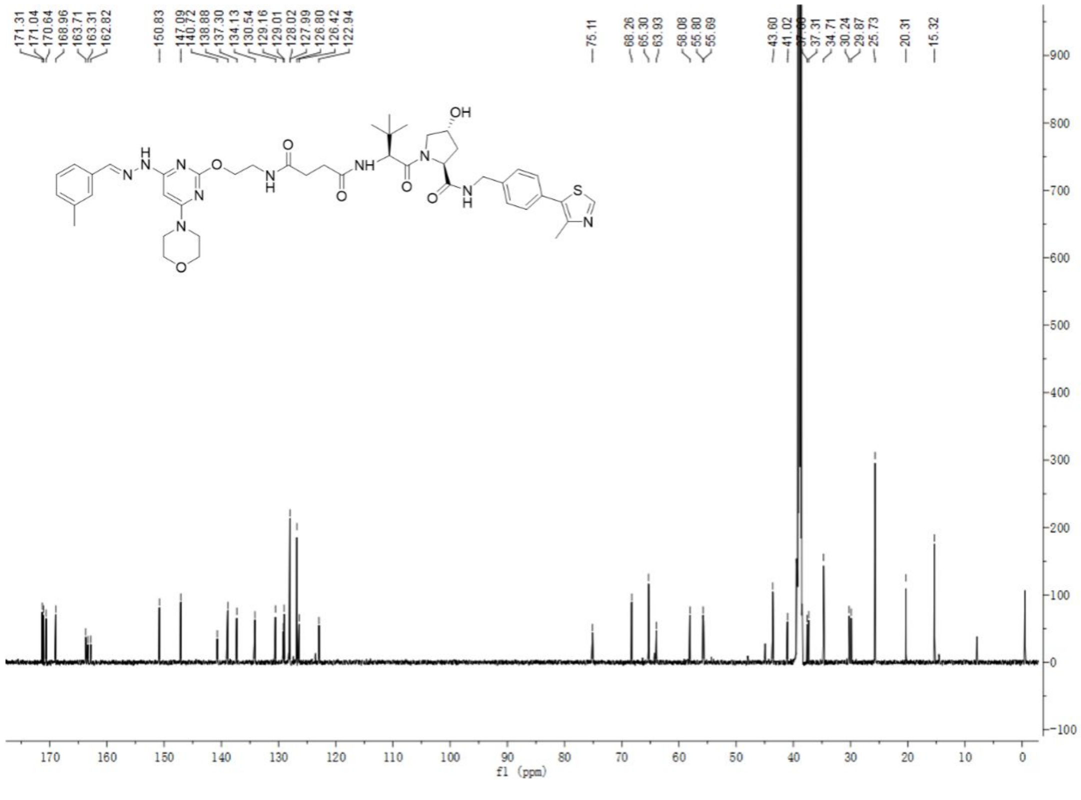

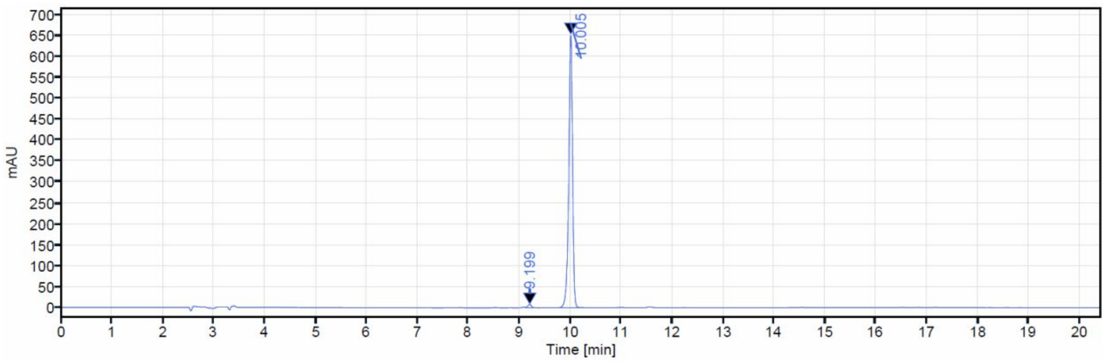

Signal: DAD1A,Sig=254,4 Ref=off

RT [min]	Type	Width [min]	Area	Height	Area%	Name
9.199	VB	0.26	43.71	8.38	1.27	
10.005	BV	0.70	3400.60	655.48	98.73	
		**Sum**	3444.32		100.00	

Compound **7c**

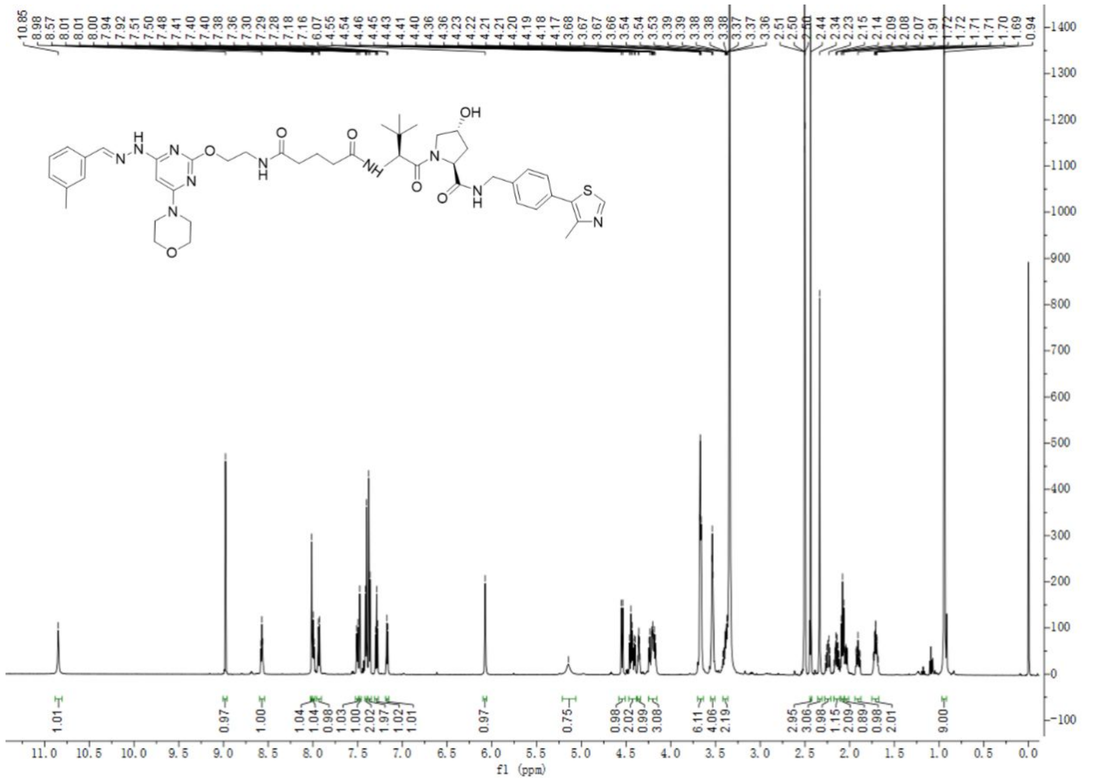

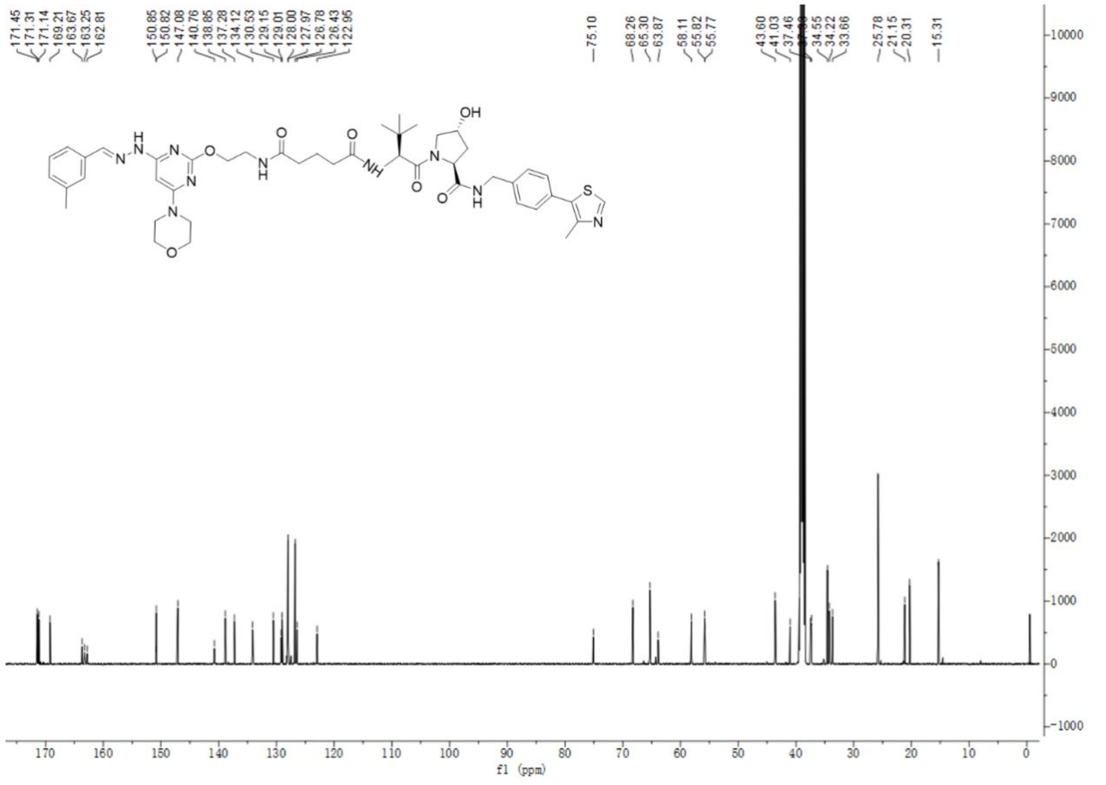

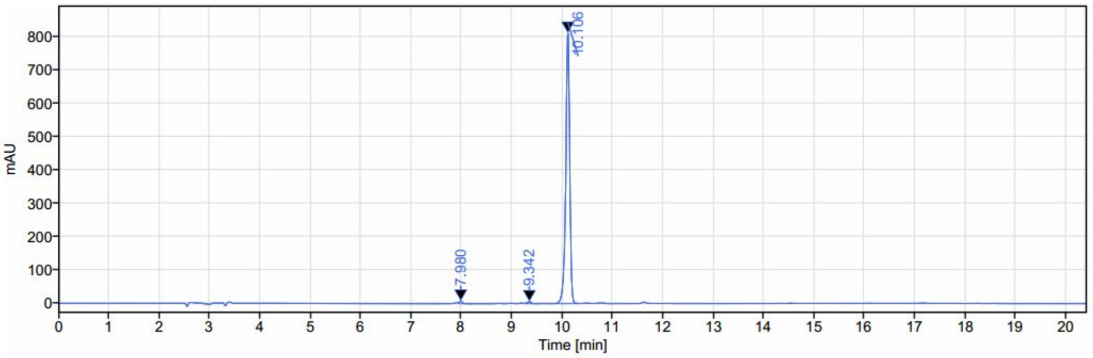

Signal: DAD1A,Sig=254,4 Ref=off

RT [min]	Type	Width [min]	Area	Height	Area%	Name
7.980	VB	0.28	35.28	8.50	0.79	
9.342	VB	0.25	35.26	6.42	0.78	
10.106	VV	0.66	4421.70	810.54	98.43	
		**Sum**	4492.24		100.00	

Compound **7d**

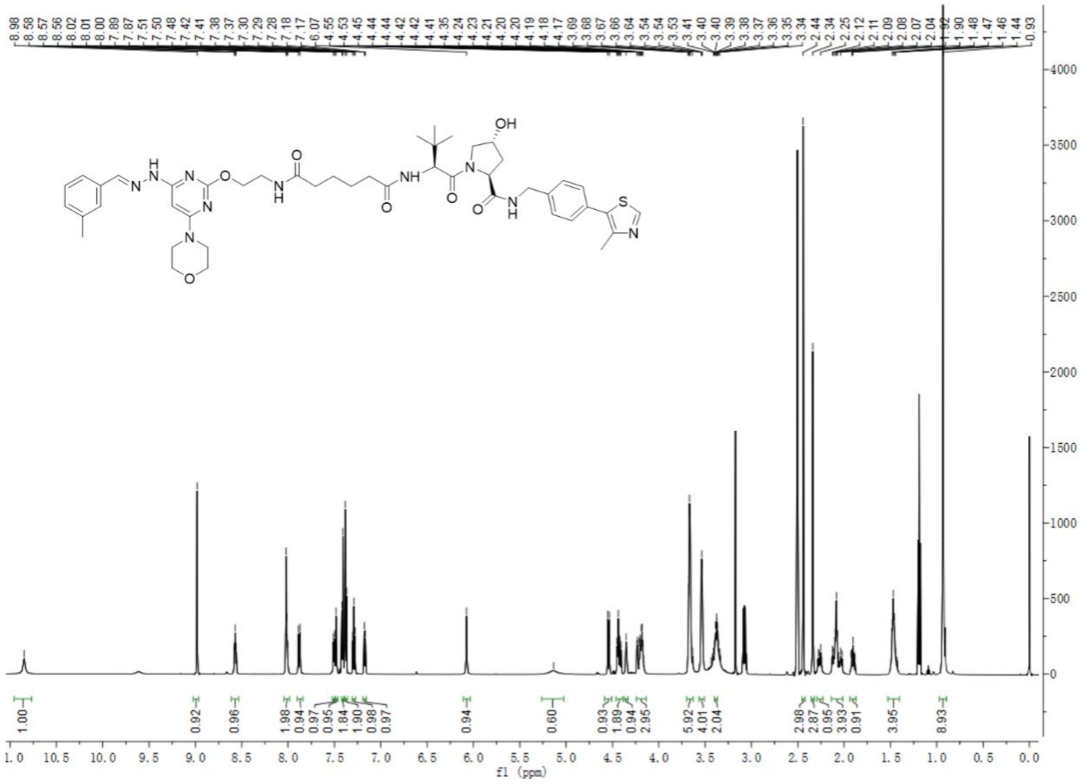

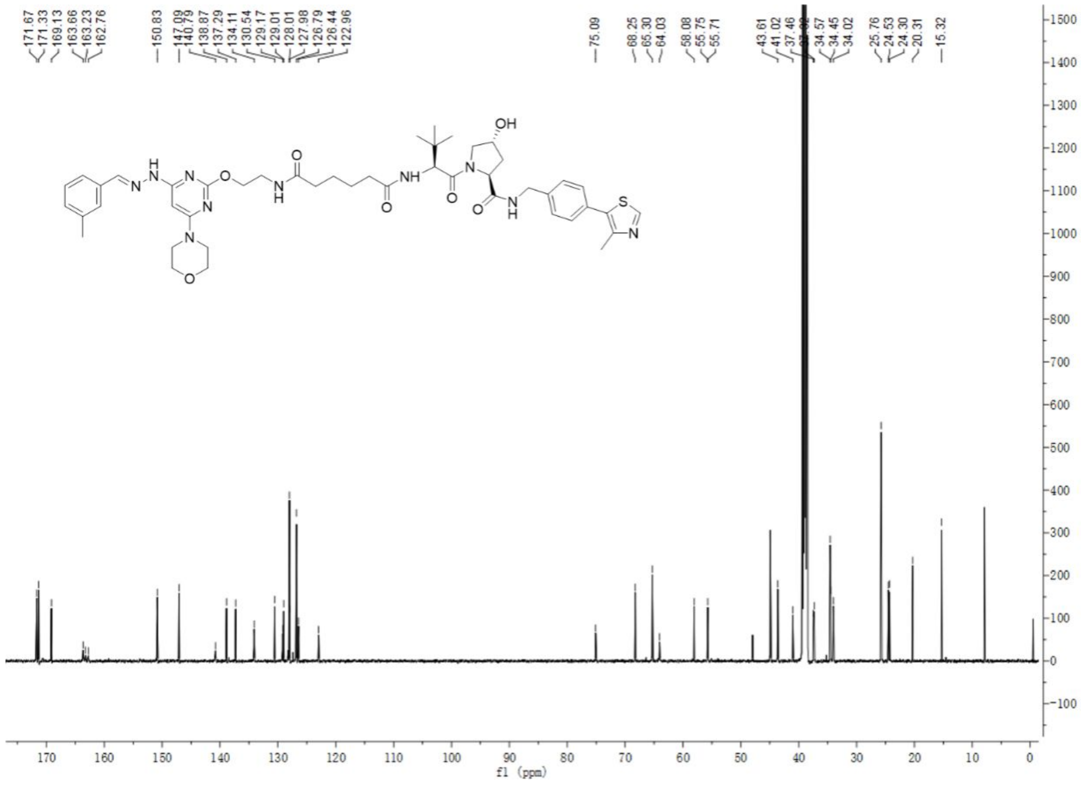

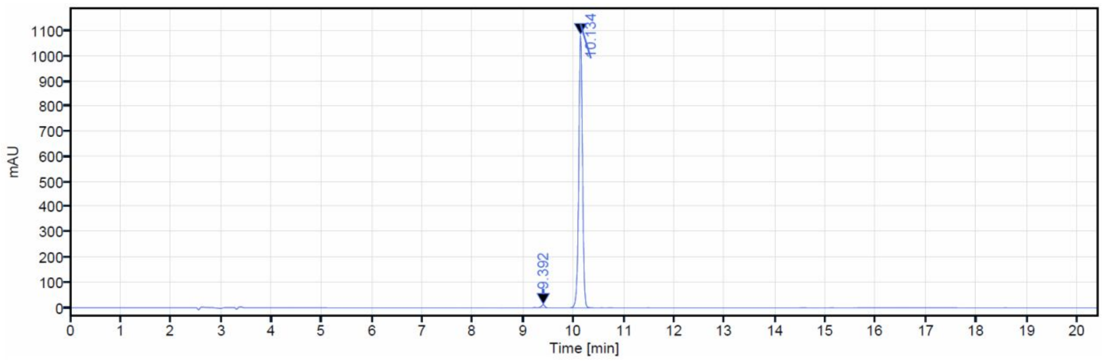

Signal: DAD1A,Sig=254,4 Ref=off

RT [min]	Type	Width [min]	Area	Height	Area%	Name
9.392	MM m	0.15	51.90	11.76	0.92	
10.134	BV	0.68	5595.25	1086.09	99.08	
		**Sum**	5647.15		100.00	

Compound **7e**

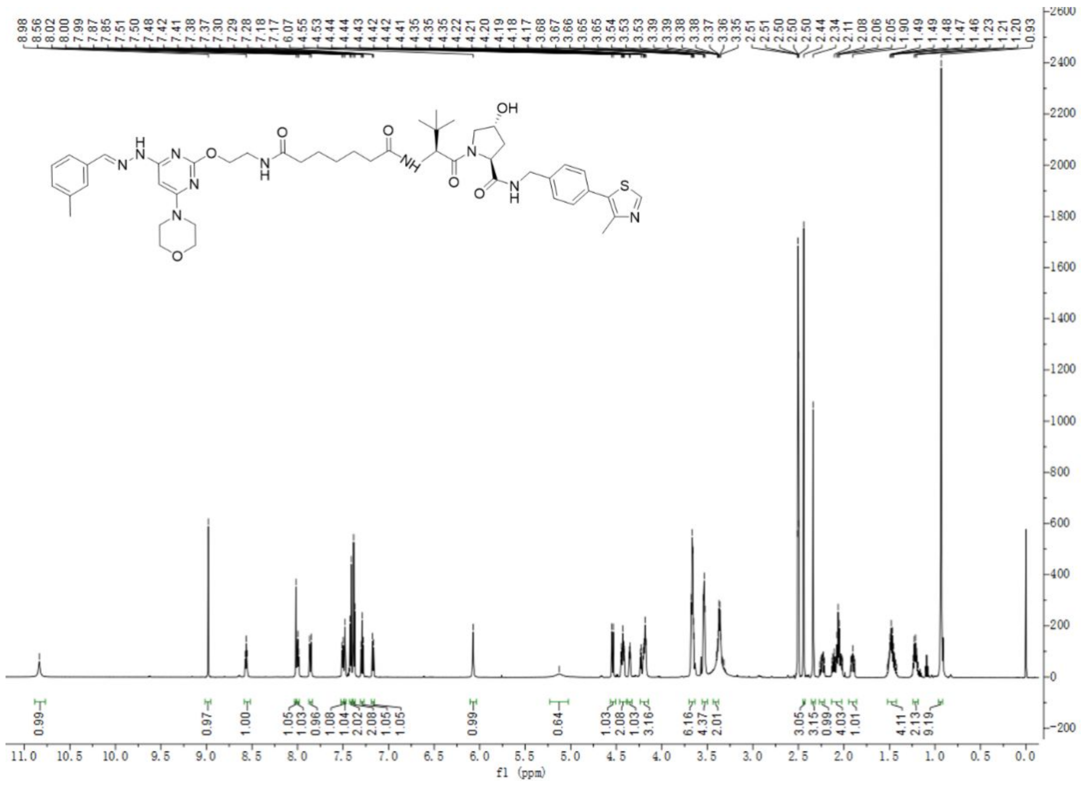

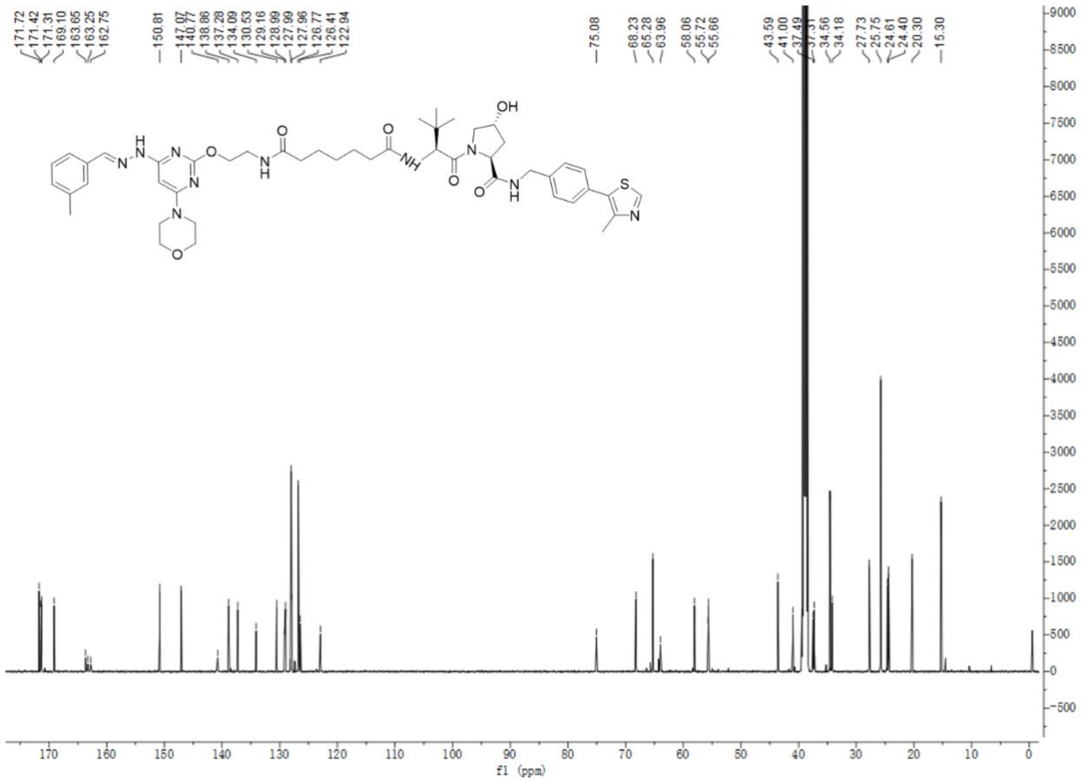

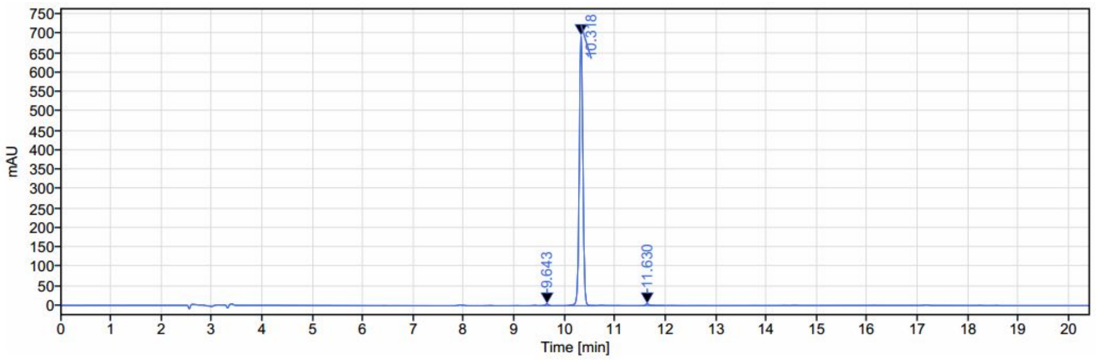

Signal: DAD1A,Sig=254,4 Ref=off

RT [min]	Type	Width [min]	Area	Height	Area%	Name
9.643	VB	0.29	24.25	4.75	0.70	
10.318	VV	0.67	3428.93	695.96	98.54	
11.630	BB	0.49	26.56	4.56	0.76	
		**Sum**	3479.74		100.00	

Compound **7f**

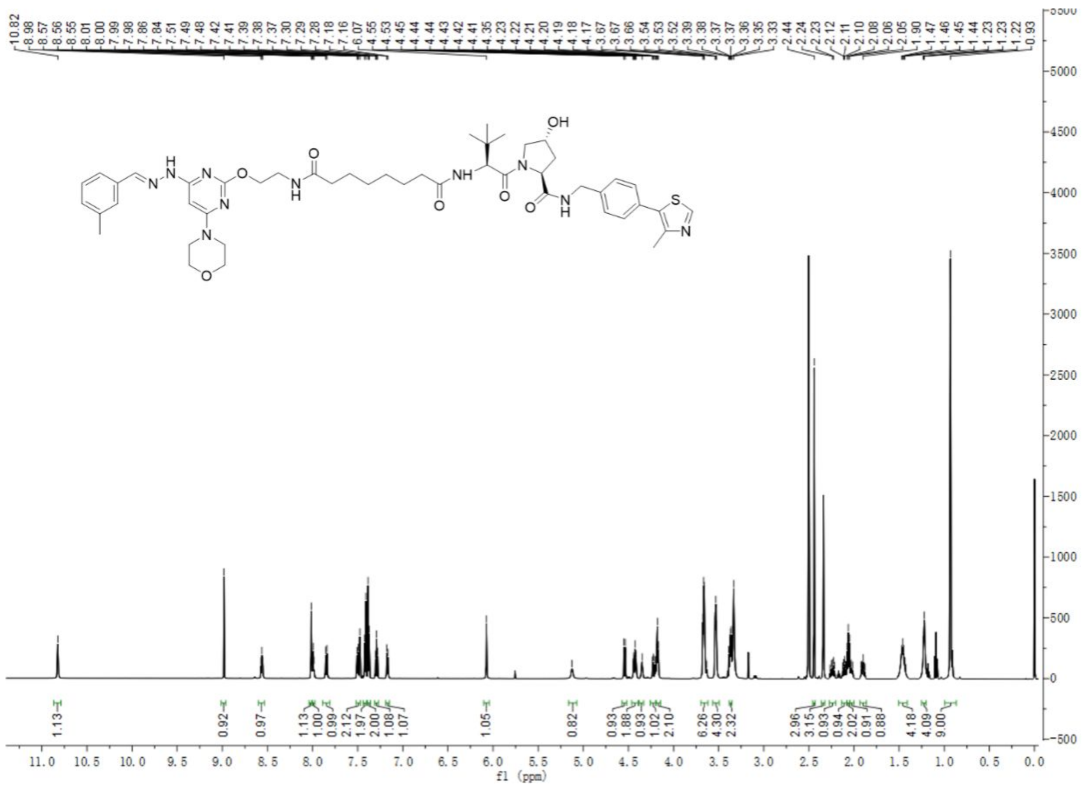

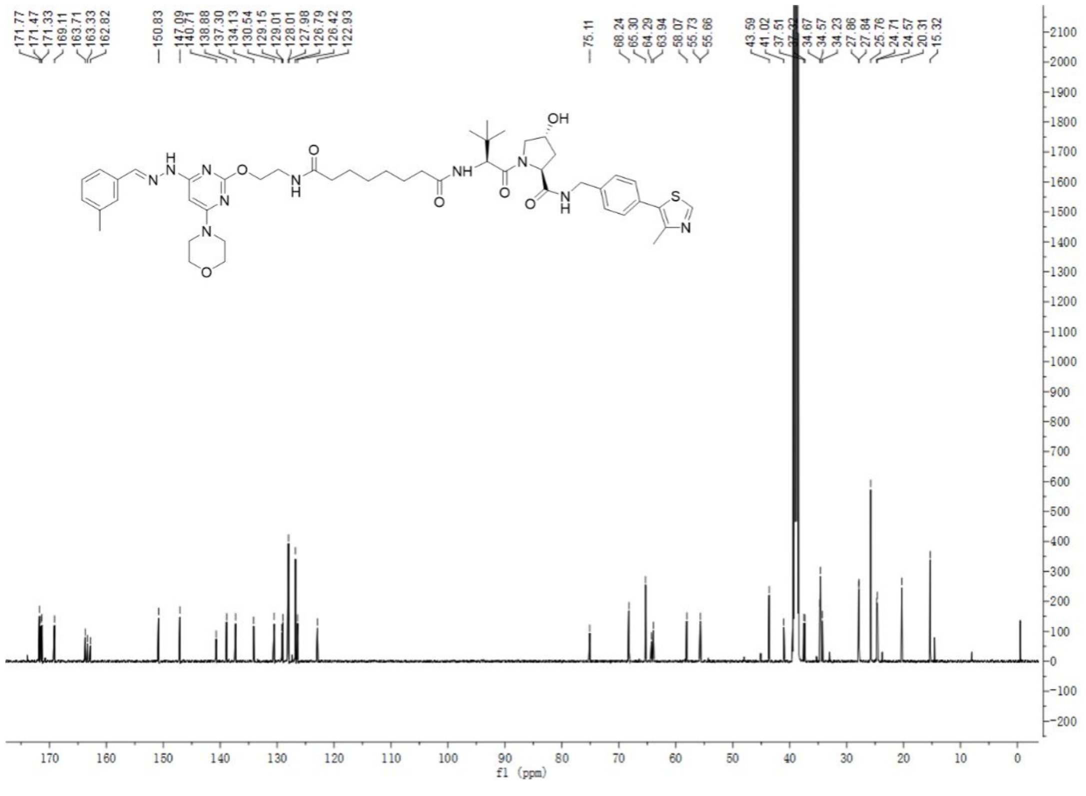

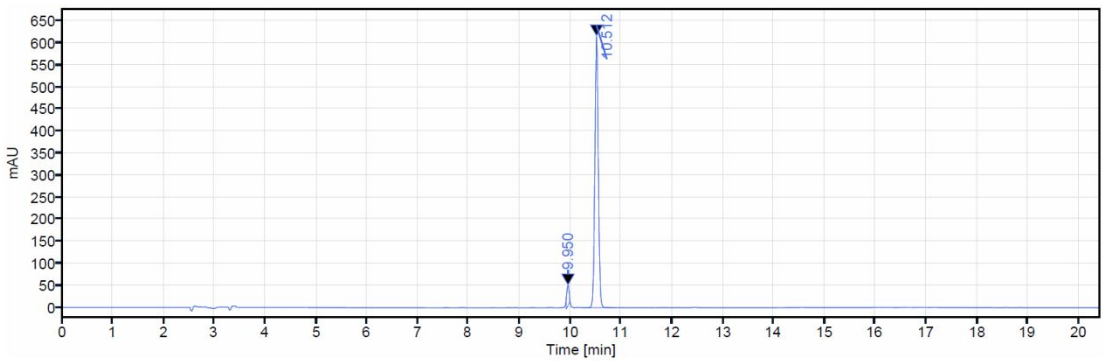

Signal: DAD1A,Sig=254,4 Ref=off

RT [min]	Type	Width [min]	Area	Height	Area%	Name
9.950	MM m	0.07	136.50	46.04	4.45	
10.512	VV	0.63	2930.35	616.79	95.55	
		**Sum**	3066.84		100.00	

Compound **7g**

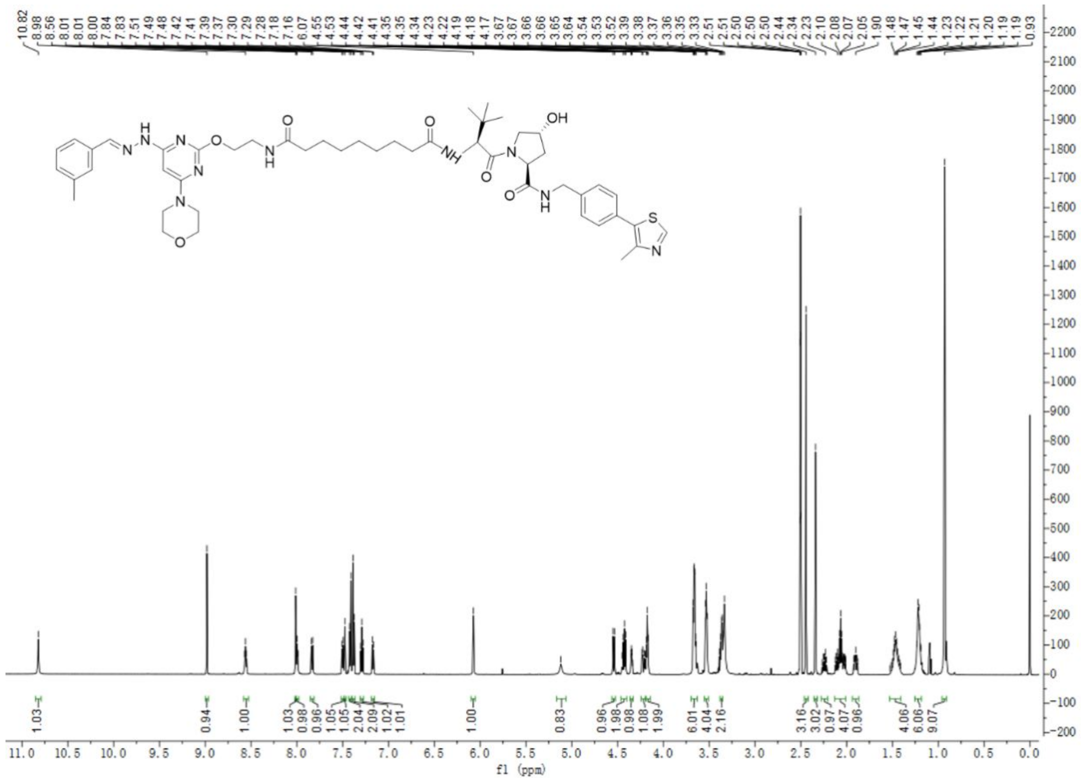

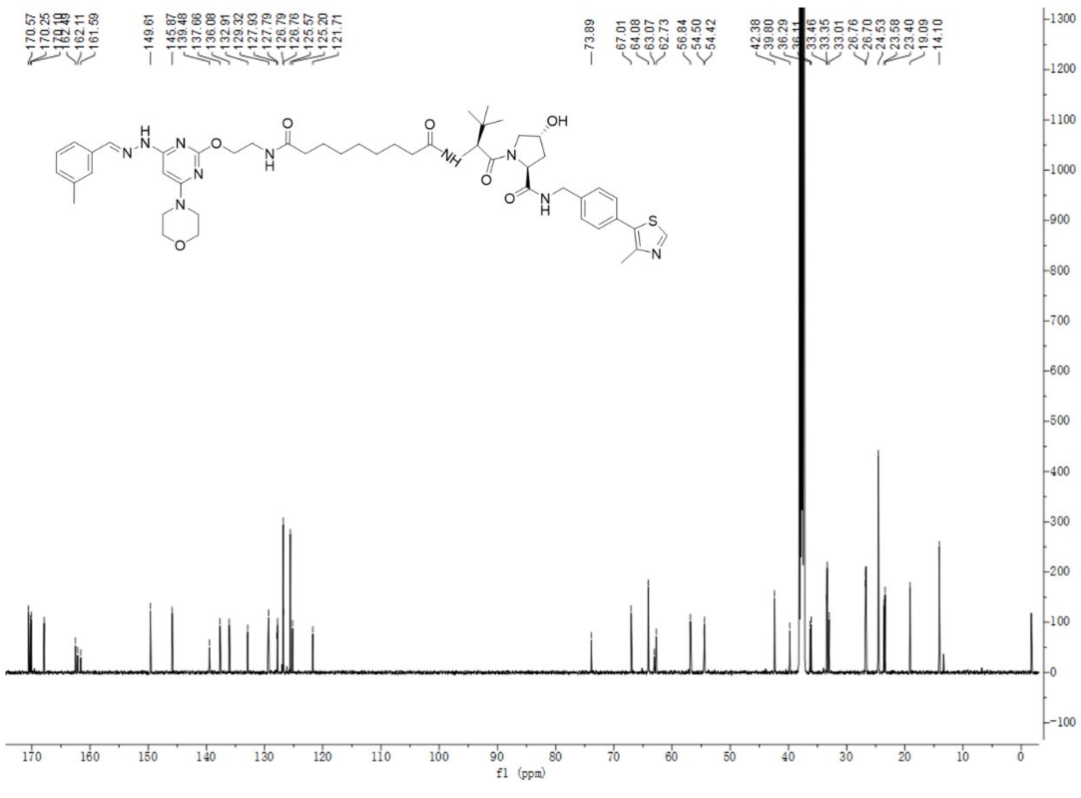

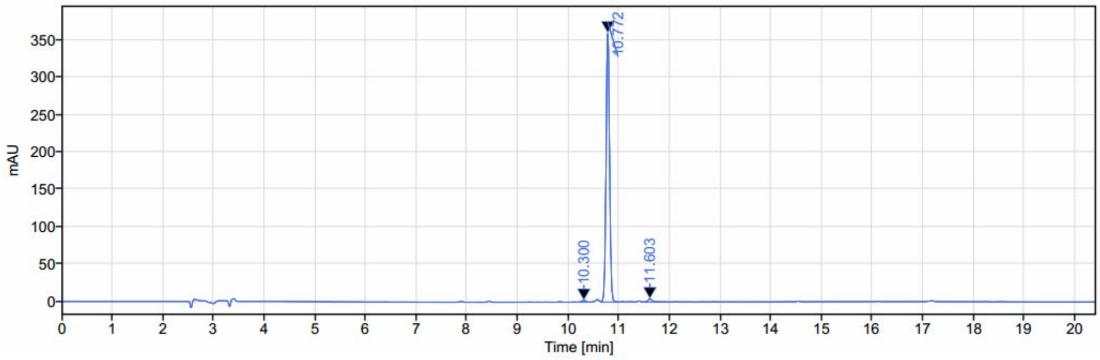

Signal: DAD1A,Sig=254,4 Ref=off

RT [min]	Type	Width [min]	Area	Height	Area%	Name
10.300	VB	0.23	12.70	2.55	0.73	
10.772	VV	0.36	1708.46	360.30	97.86	
11.603	VB	0.47	24.57	4.46	1.41	
		**Sum**	1745.73		100.00	

Compound **7h**

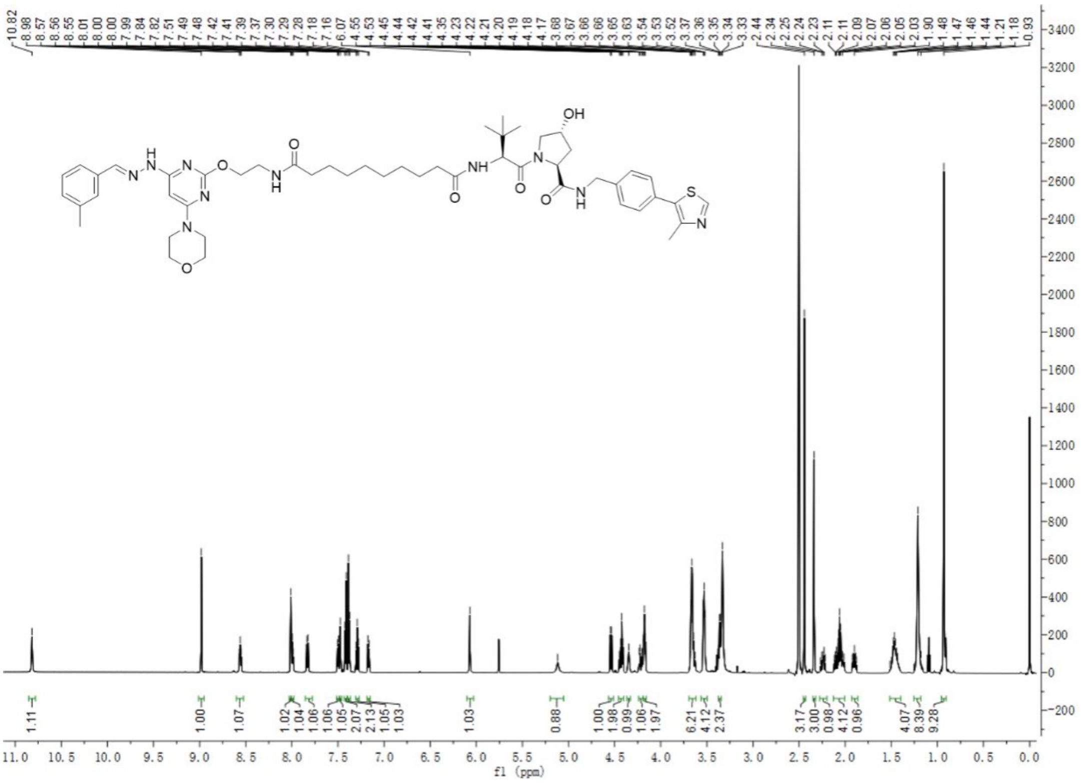

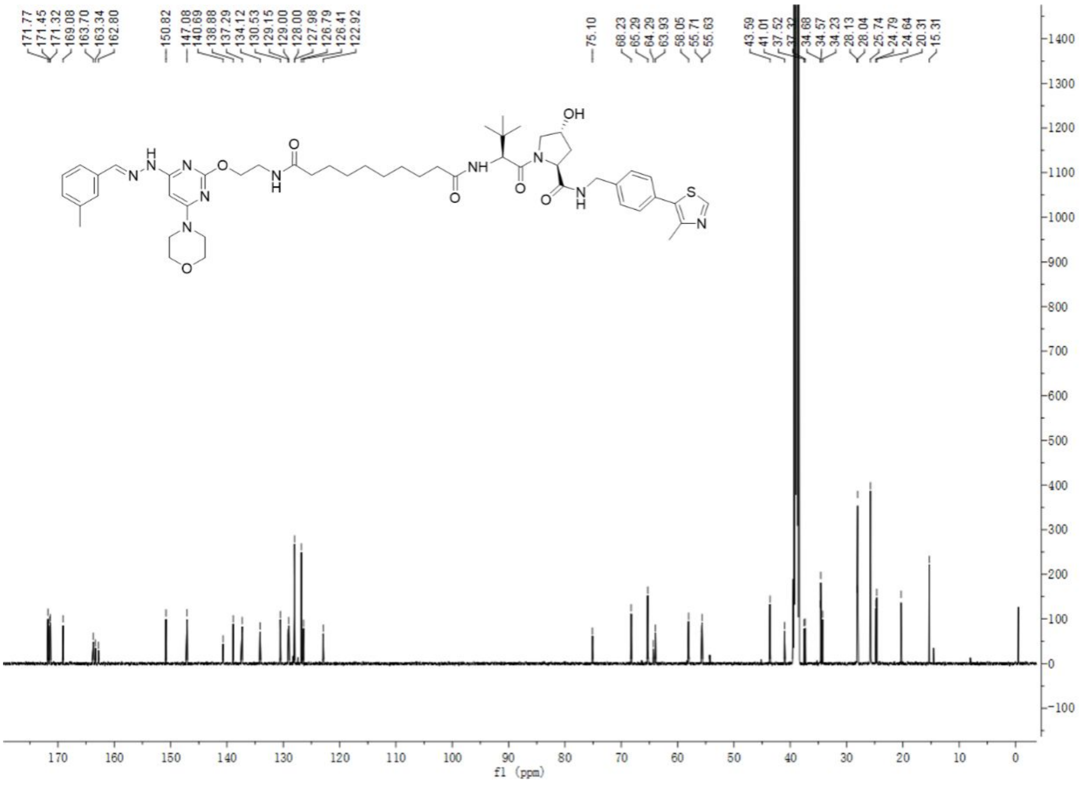

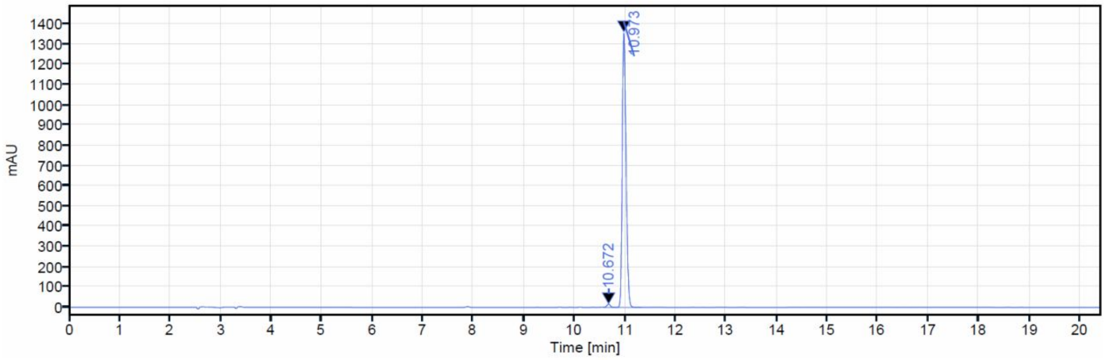

Signal: DAD1A,Sig=254,4 Ref=off

RT [min]	Type	Width [min]	Area	Height	Area%	Name
10.672	VV	0.24	85.25	16.42	1.18	
10.973	VV	0.51	7138.02	1360.86	98.82	
		**Sum**	7223.27		100.00	

Compound **7i**

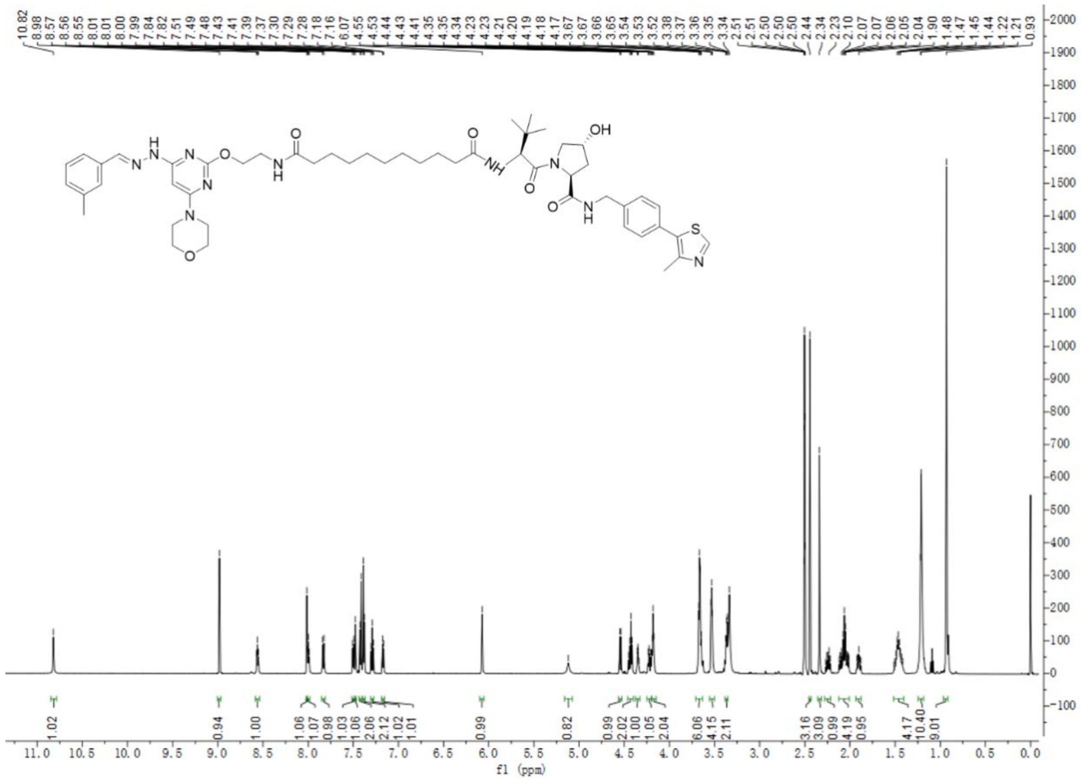

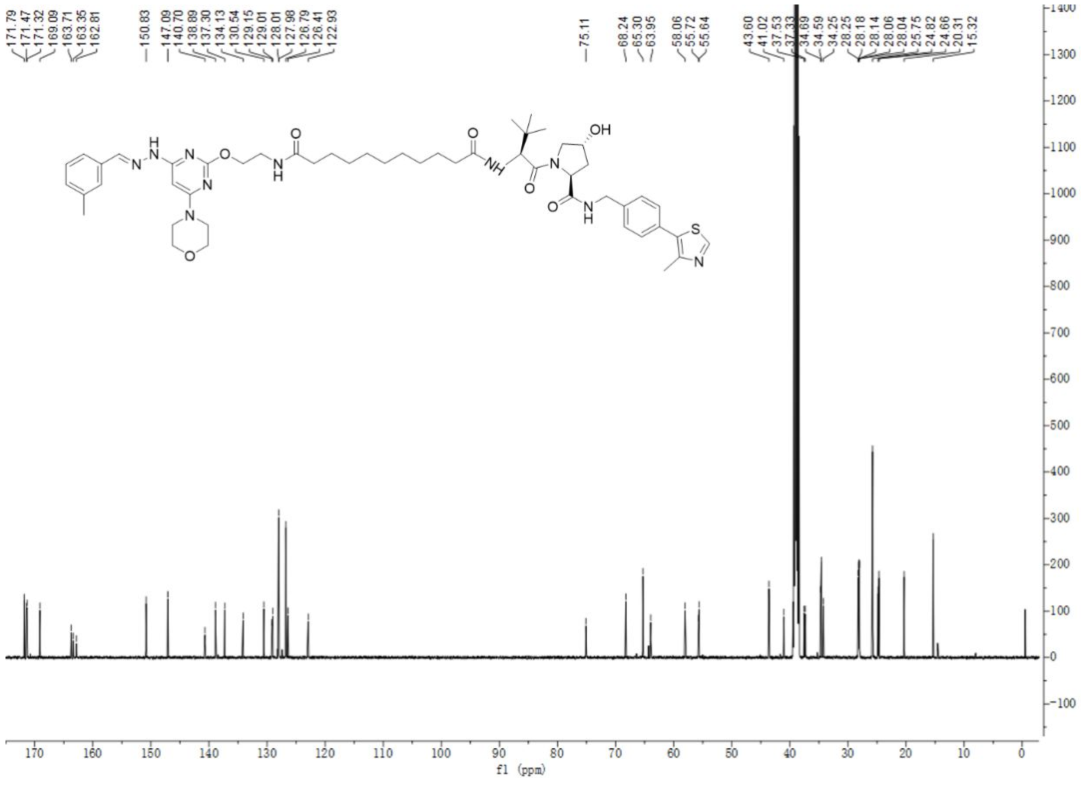

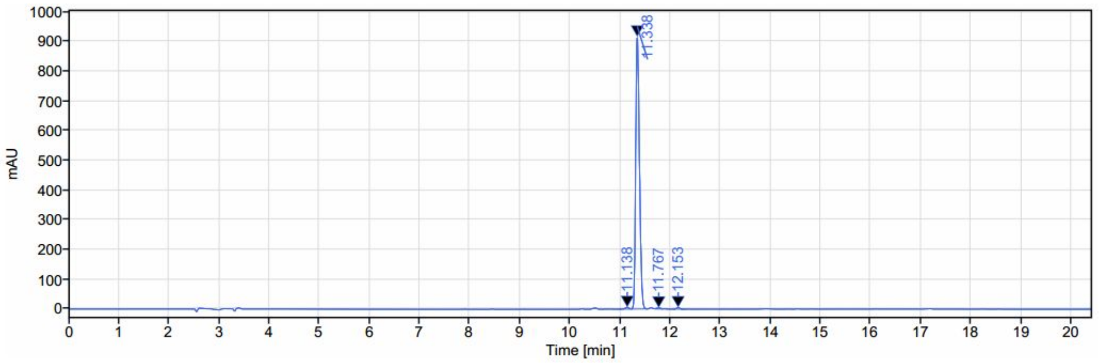

Signal: DAD1A,Sig=254,4 Ref=off

RT [min]	Type	Width [min]	Area	Height	Area%	Name
11.138	BV	0.25	32.25	6.08	0.67	
11.338	VB	0.33	4764.93	916.26	98.36	
11.767	VV	0.17	23.28	3.86	0.48	
12.153	BB	0.25	24.11	5.15	0.50	
		**Sum**	4844.57		100.00	

Compound **7j**

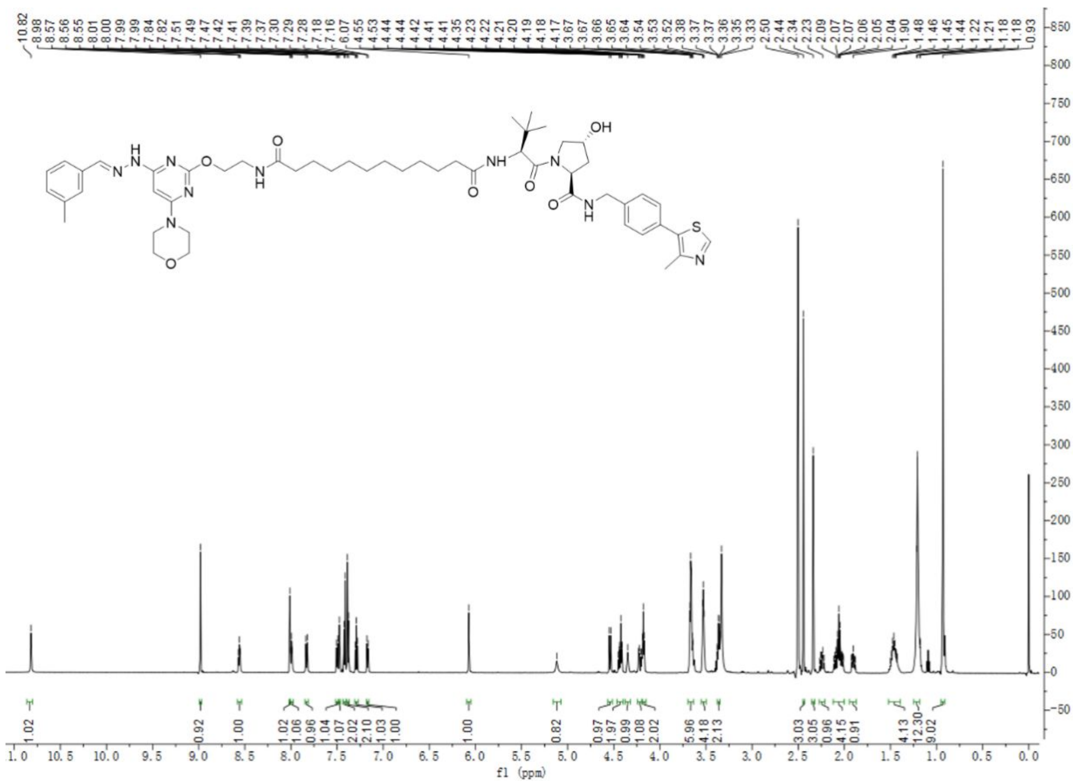

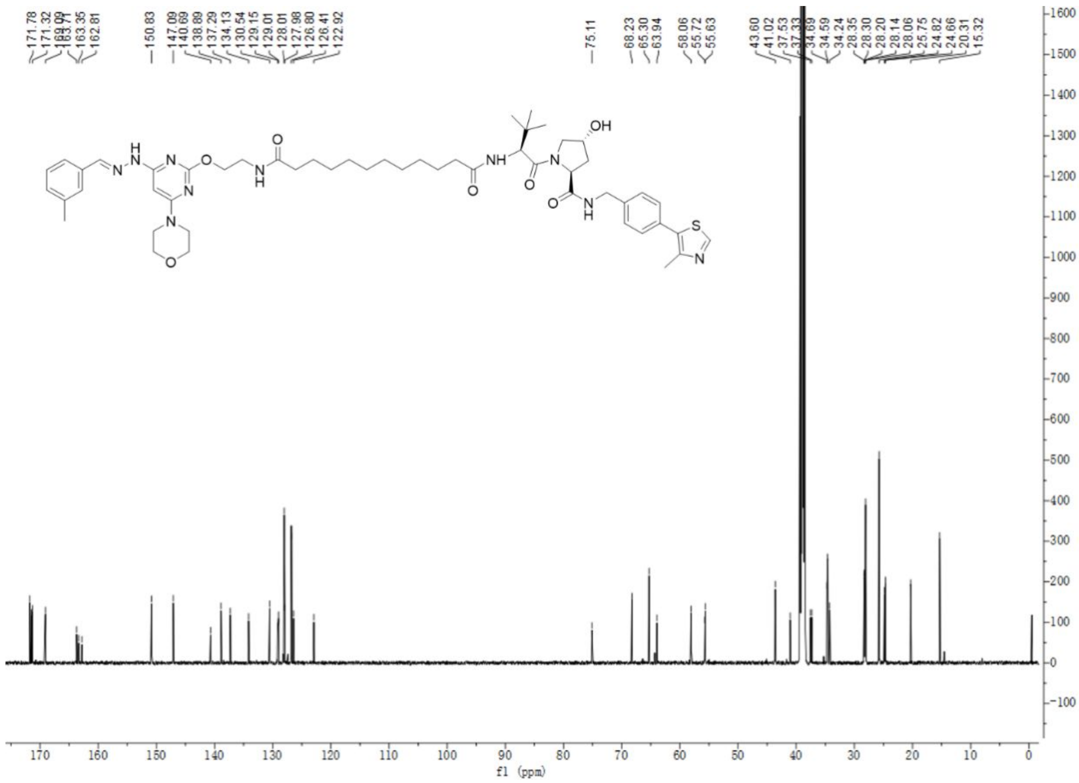

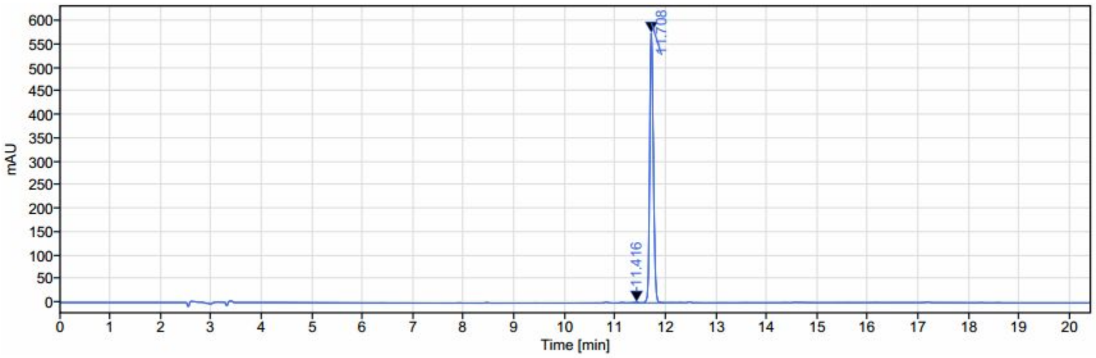

Signal: DAD1A,Sig=254,4 Ref=off

RT [min]	Type	Width [min]	Area	Height	Area%	Name
11.416	VV	0.29	10.50	1.72	0.36	
11.708	VV	0.48	2901.01	574.44	99.64	
		**Sum**	2911.51		100.00	

Compound **12a**

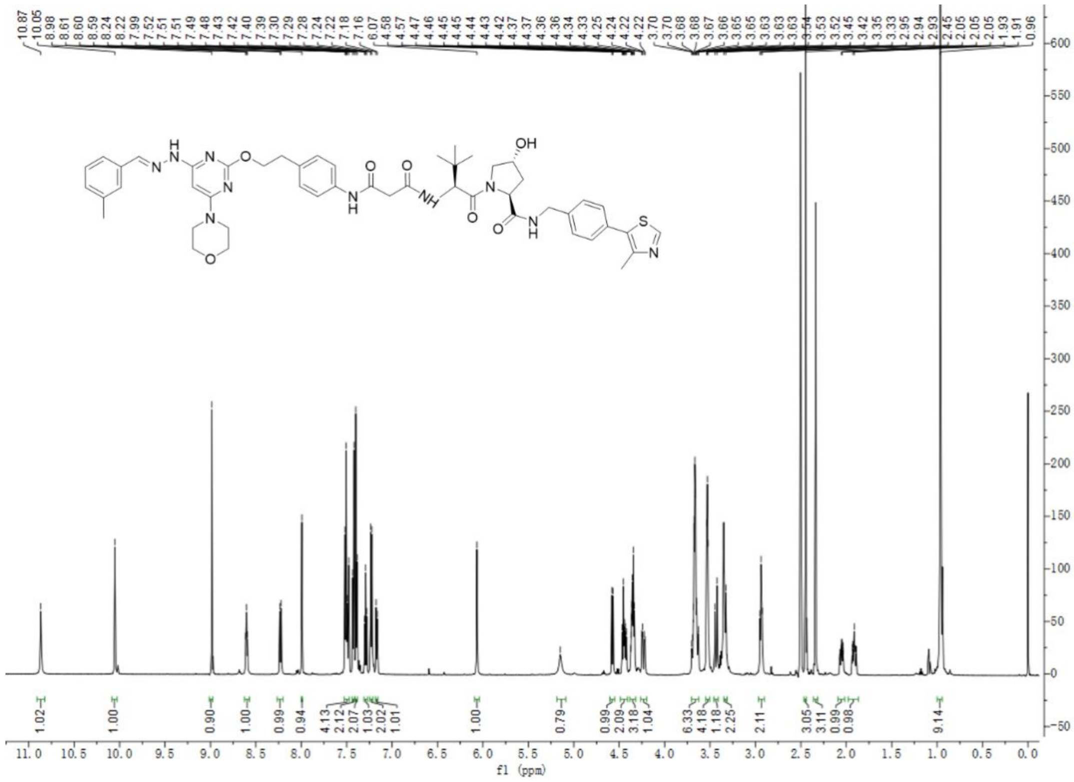

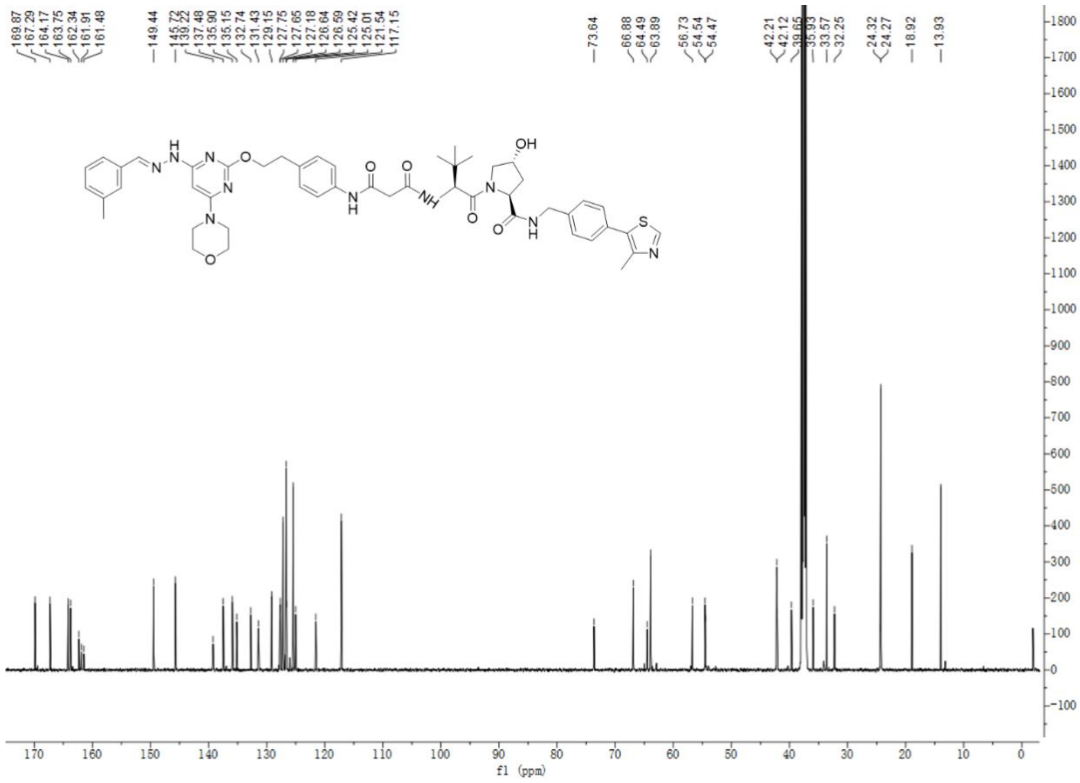

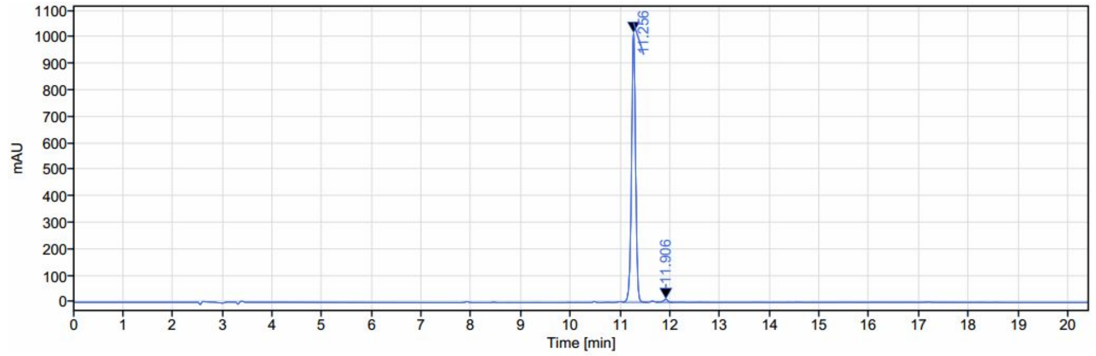

Signal: DAD1A,Sig=254,4 Ref=off

RT [min]	Type	Width [min]	Area	Height	Area%	Name
11.256	VB	0.49	5800.71	1016.46	98.68	
11.906	VB	0.34	77.57	11.73	1.32	
		**Sum**	5878.28		100.00	

Compound **12b**

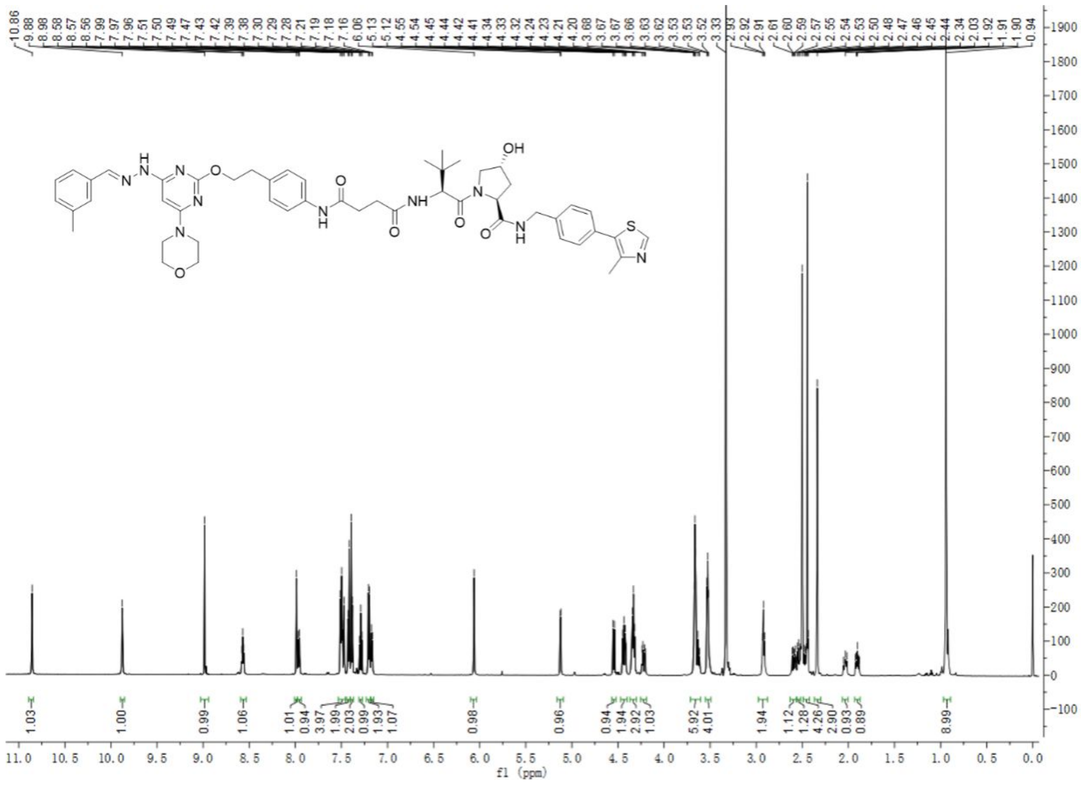

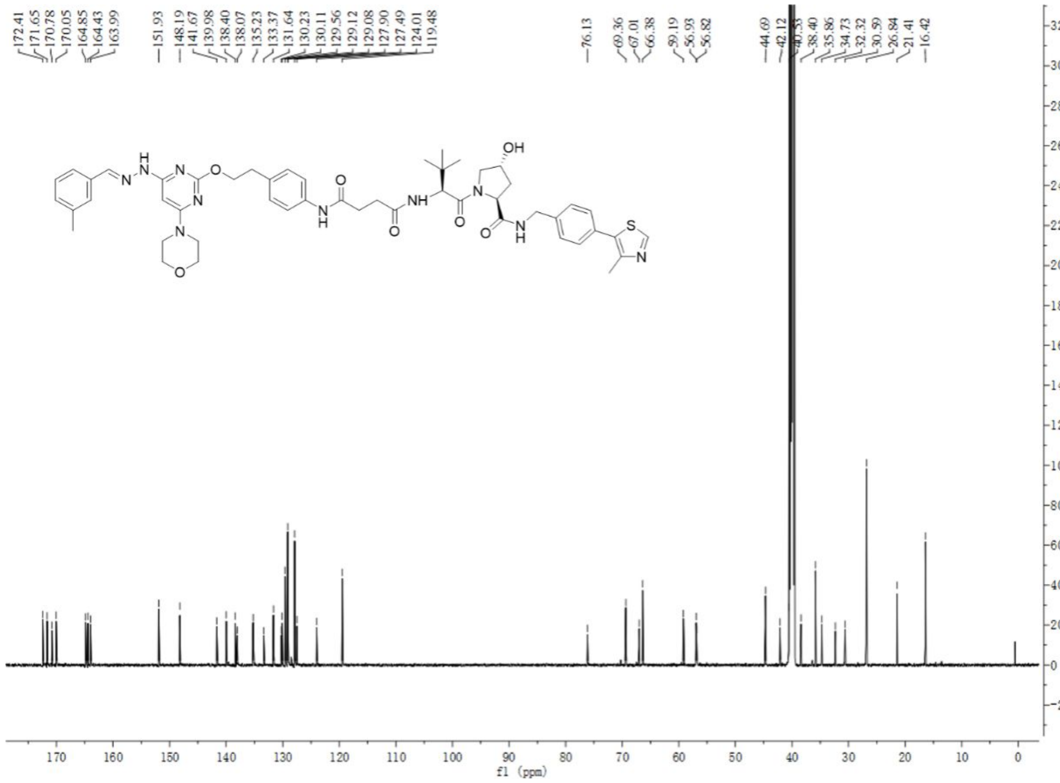

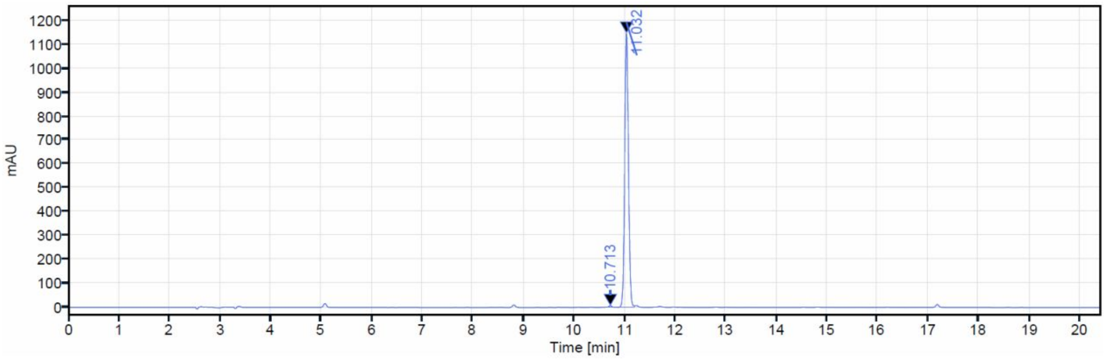

Signal: DAD1A,Sig=254,4 Ref=off

RT [min]	Type	Width [min]	Area	Height	Area%	Name
10.713	MM m	0.11	19.40	5.93	0.33	
11.032	BV	0.32	5896.41	1147.04	99.67	
		**Sum**	5915.81		100.00	

Compound **12c**

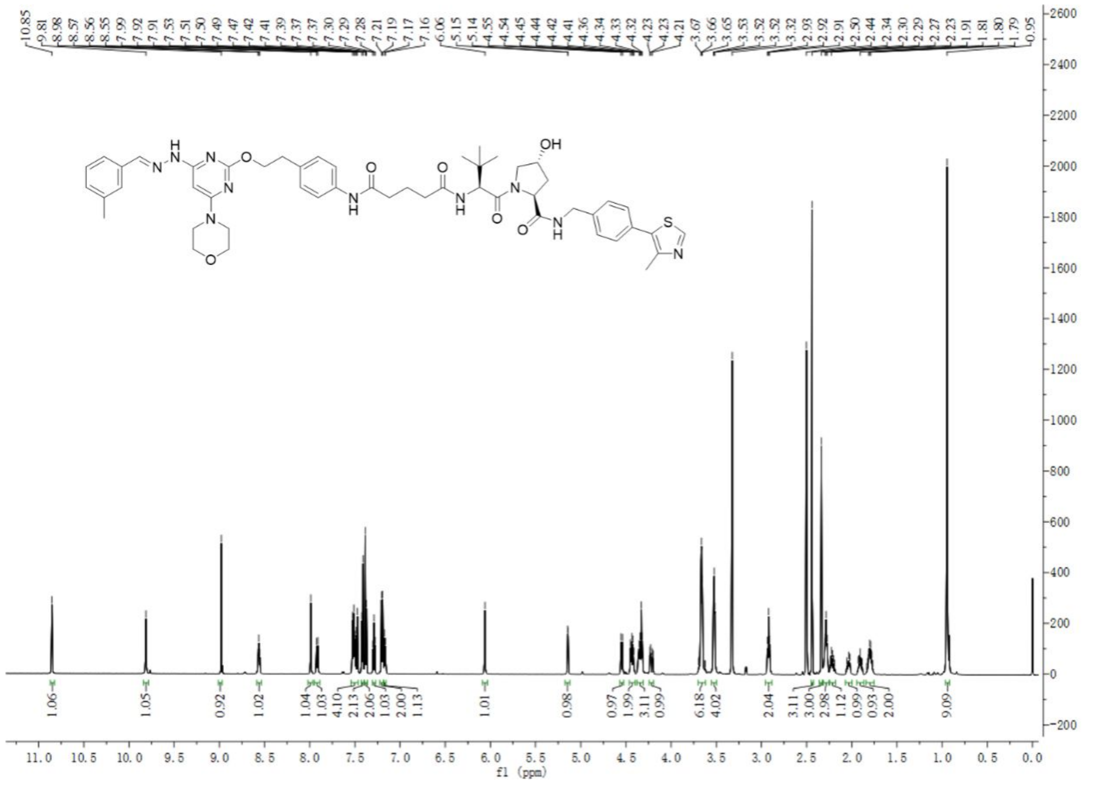

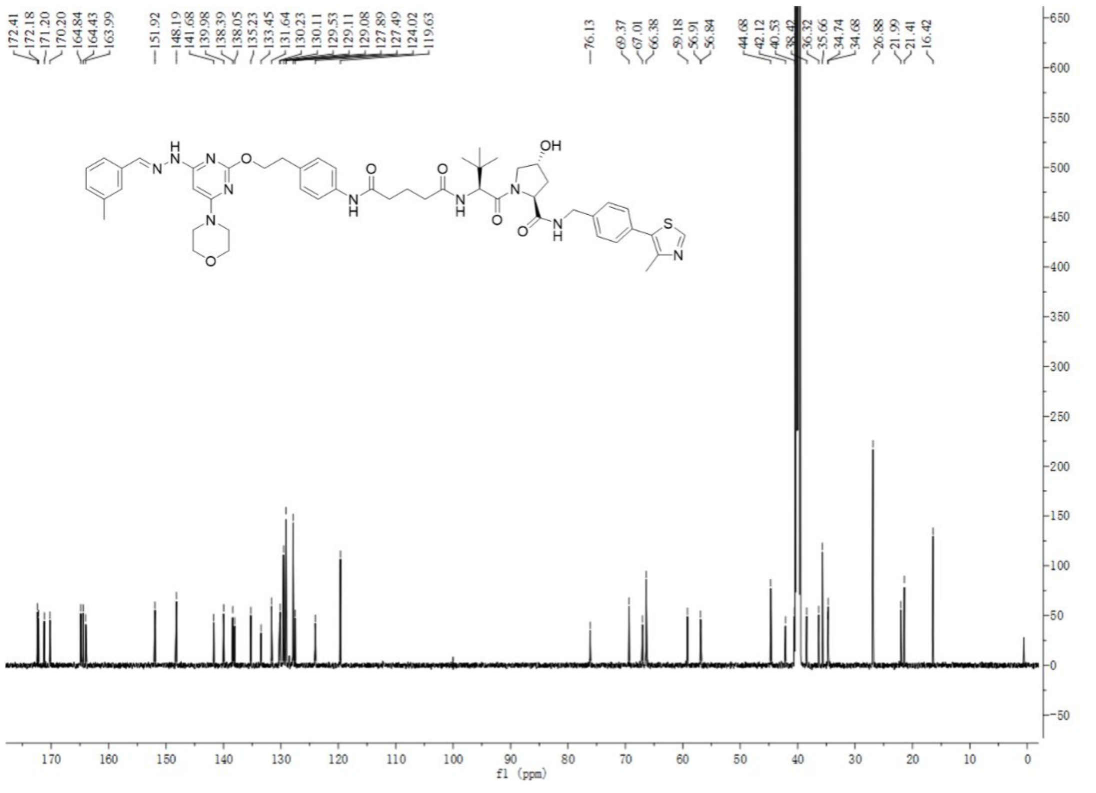

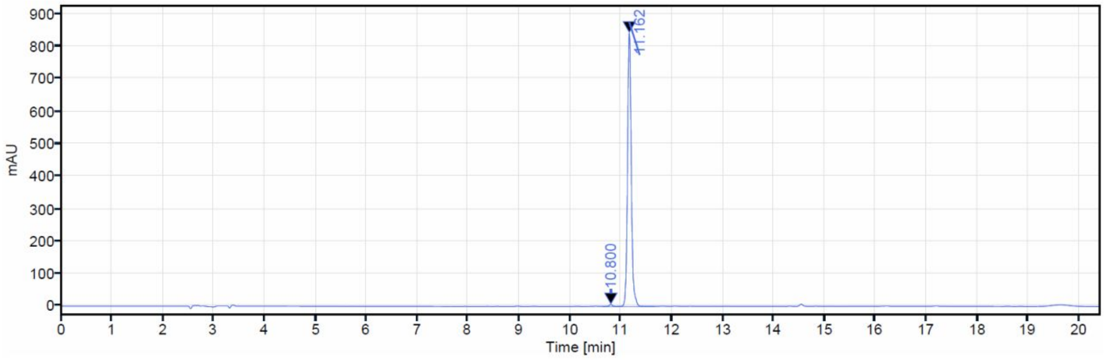

Signal: DAD1A,Sig=254,4 Ref=off

RT [min]	Type	Width [min]	Area	Height	Area%	Name
10.800	VV	0.30	31.43	6.64	0.72	
11.162	VV	0.73	4311.28	842.84	99.28	
		**Sum**	4342.71		100.00	

Compound **12d**

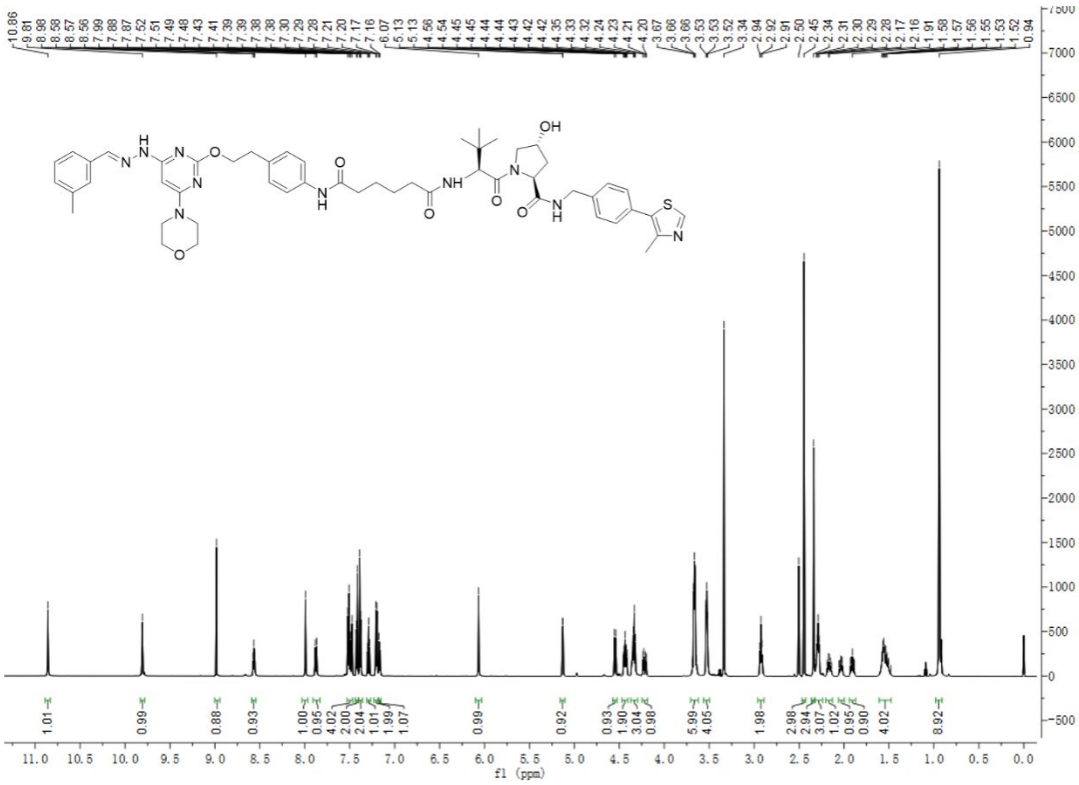

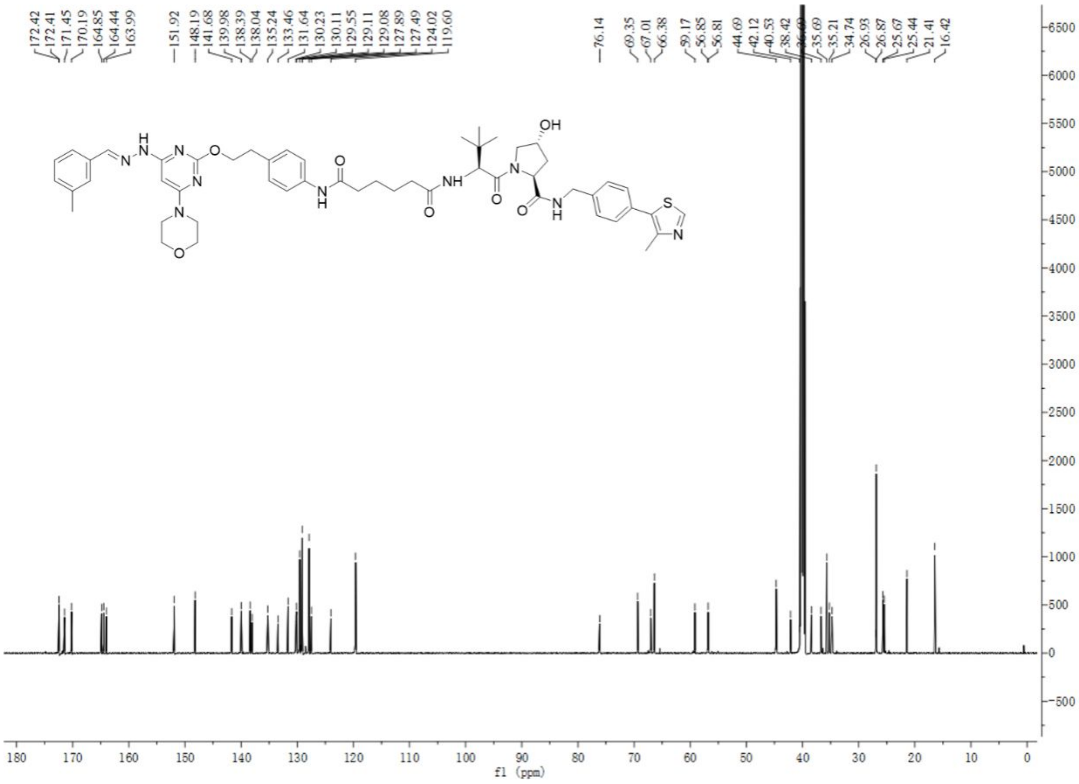

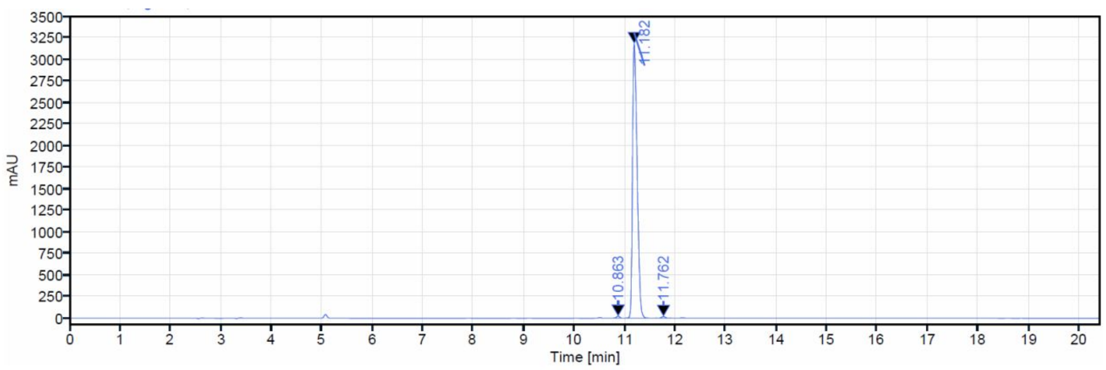

Signal: DAD1A,Sig=254,4 Ref=off

RT [min]	Type	Width [min]	Area	Height	Area%	Name
10.863	VB	0.22	106.41	22.51	0.52	
11.182	BB	0.59	20180.56	3185.79	99.00	
11.762	VV	0.20	98.33	19.57	0.48	
		**Sum**	20385.29		100.00	

Compound 12e

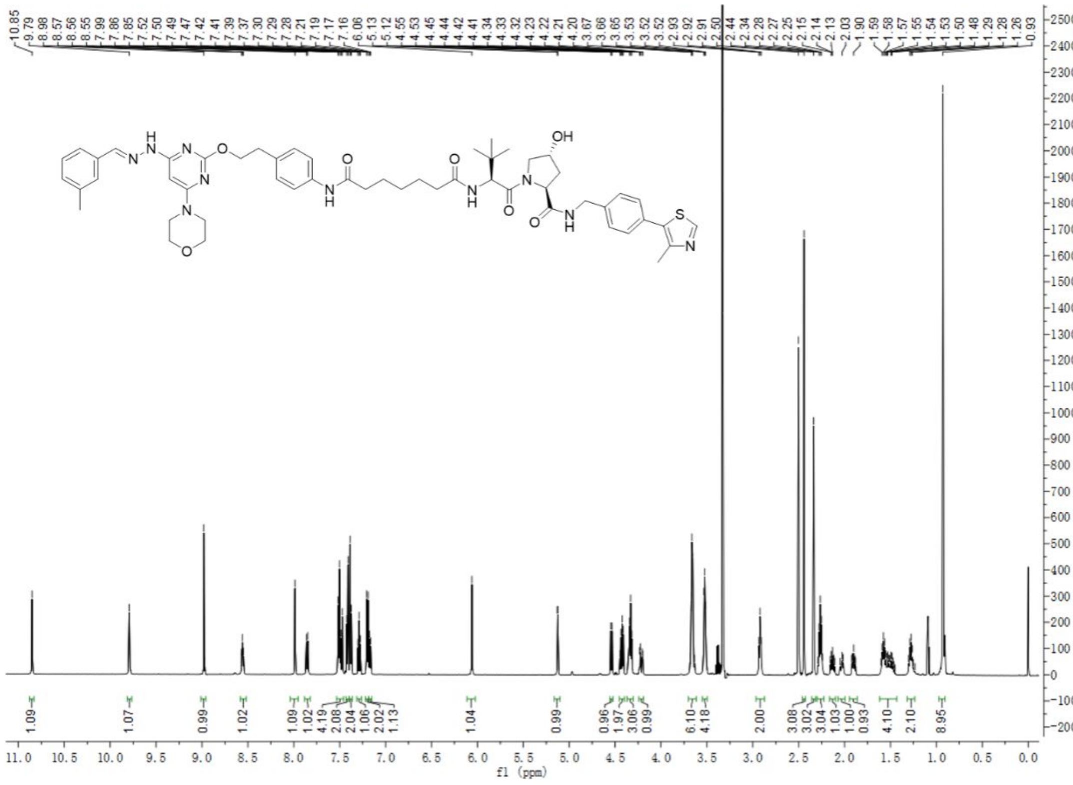

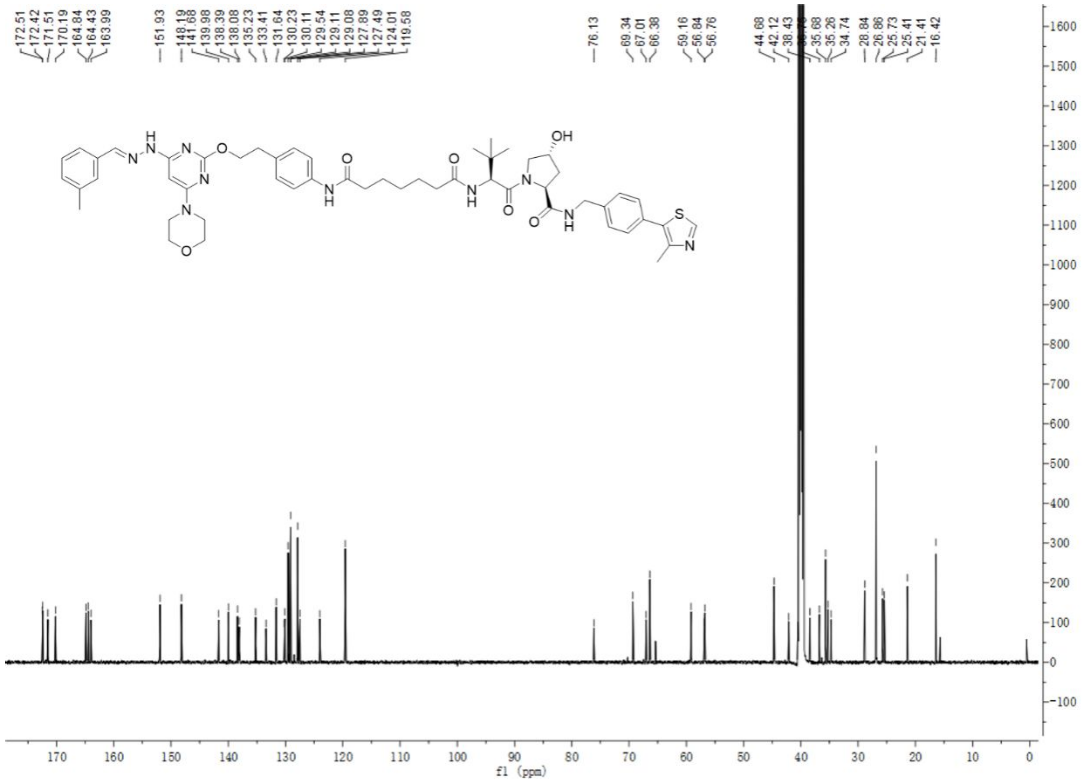

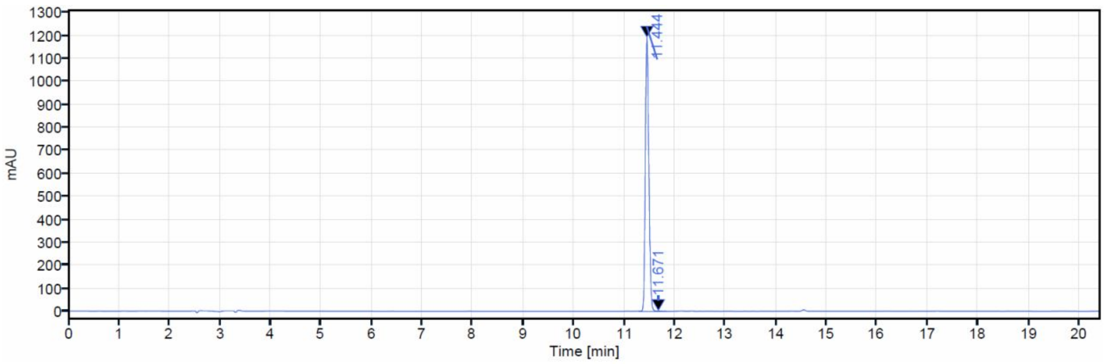

Signal: DAD1A,Sig=254,4 Ref=off

RT [min]	Type	Width [min]	Area	Height	Area%	Name
11.444	VV	0.35	5748.64	1192.91	99.85	
11.671	VB	0.24	8.86	1.85	0.15	
		**Sum**	5757.50		100.00	

Compound **12f**

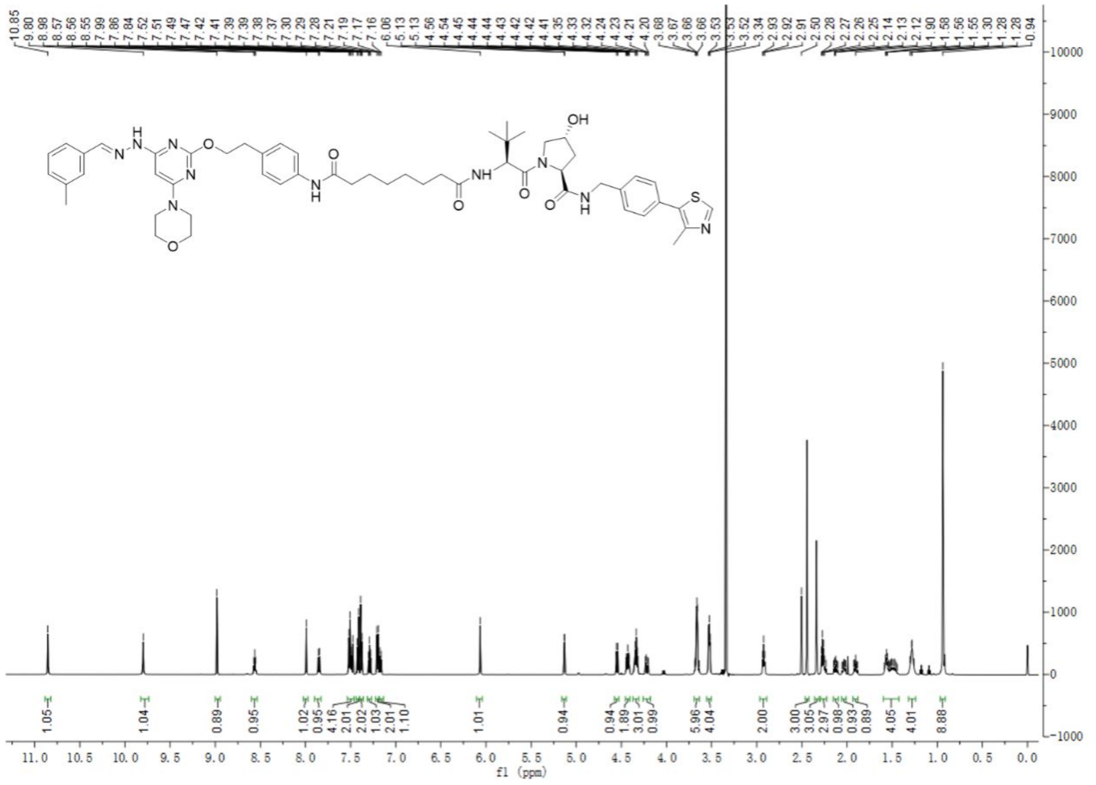

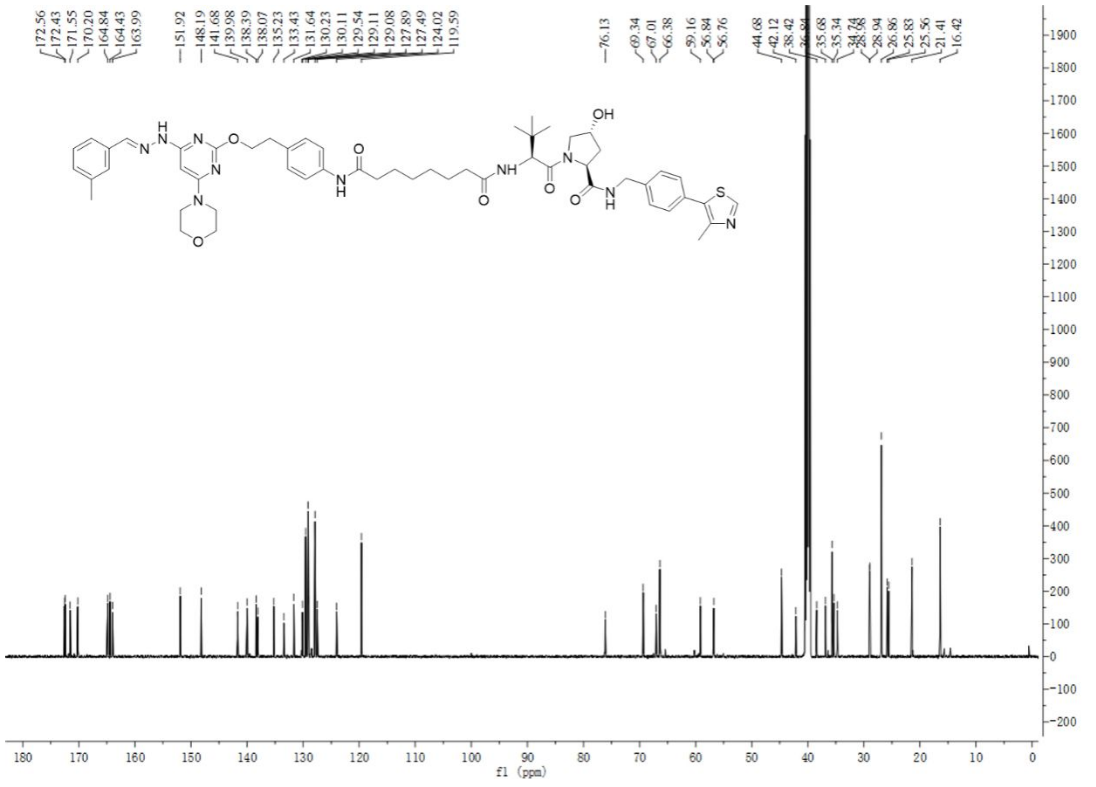

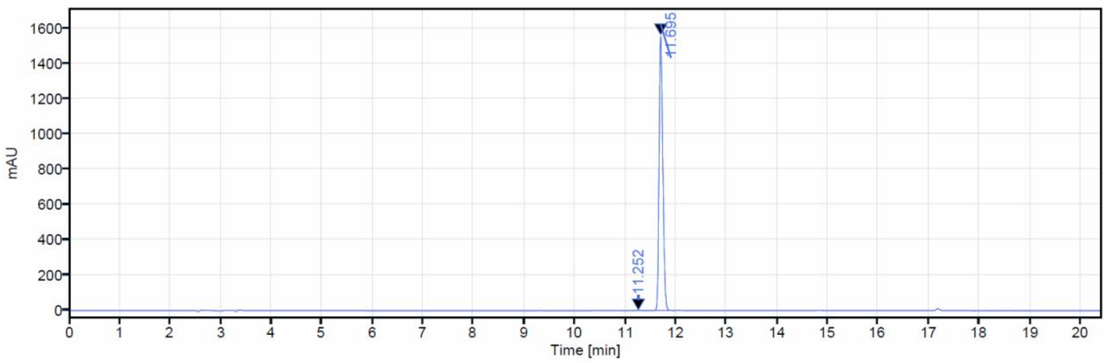

Signal: DAD1A,Sig=254,4 Ref=off

RT [min]	Type	Width [min]	Area	Height	Area%	Name
11.252	VB	0.26	21.25	2.48	0.26	
11.695	BV	0.56	8063.57	1564.25	99.74	
		**Sum**	8084.82		100.00	

Compound **12g**

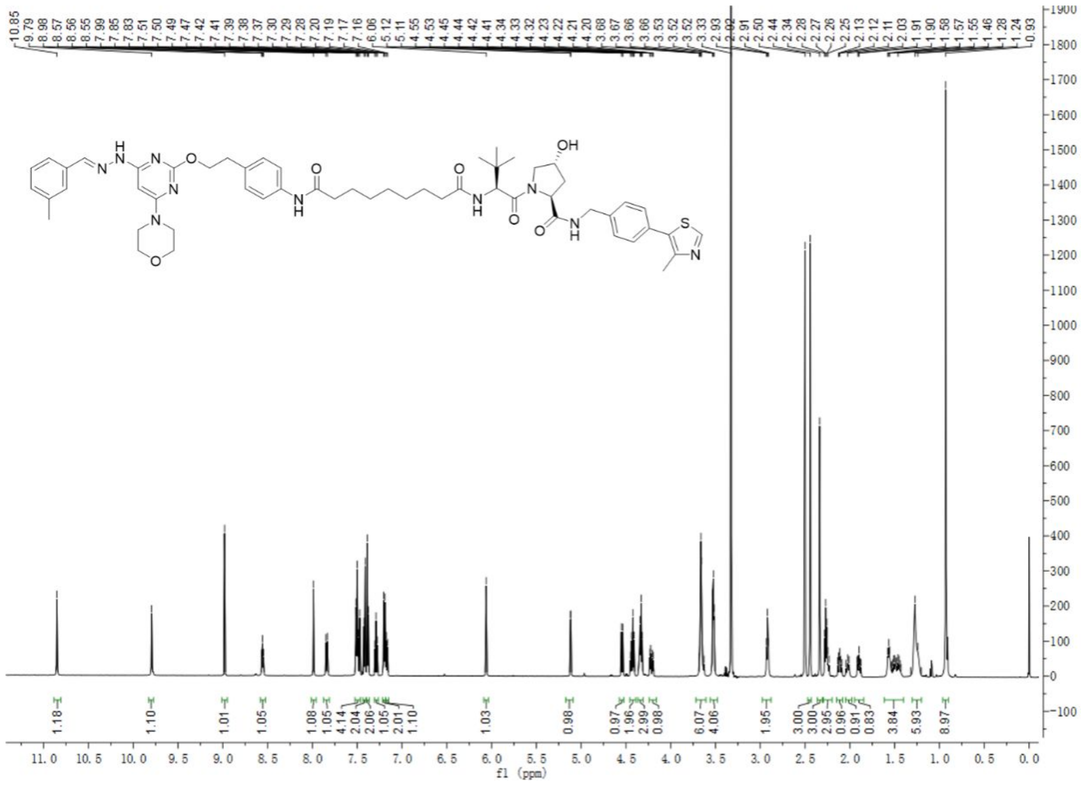

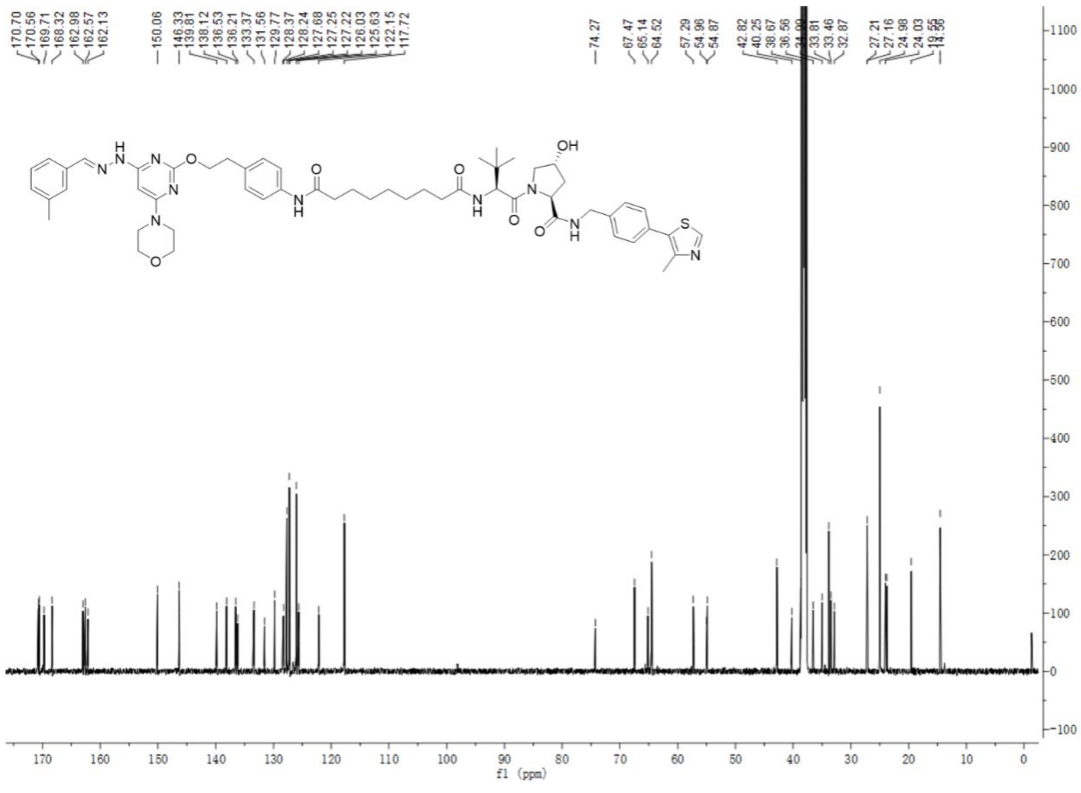

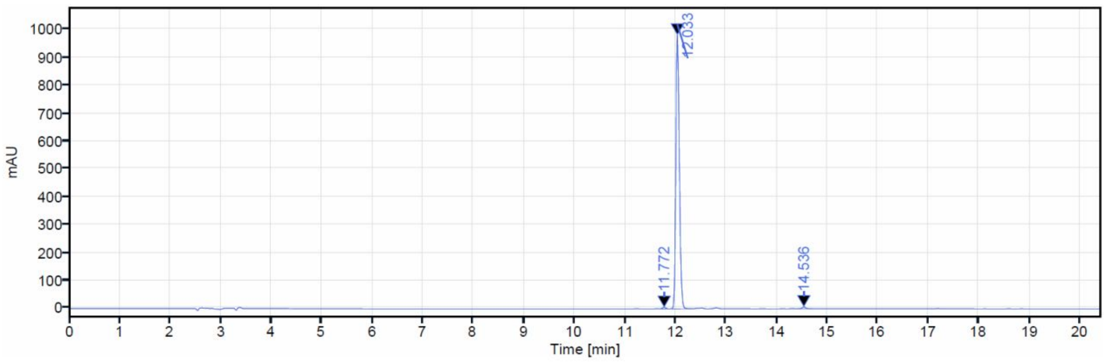

Signal: DAD1A,Sig=254,4 Ref=off

RT [min]	Type	Width [min]	Area	Height	Area%	Name
11.772	VB	0.19	23.32	5.25	0.48	
12.033	BB	0.46	4783.57	982.78	98.87	
14.536	VB	0.27	31.17	6.84	0.64	
		**Sum**	4838.07		100.00	

Compound **12h**

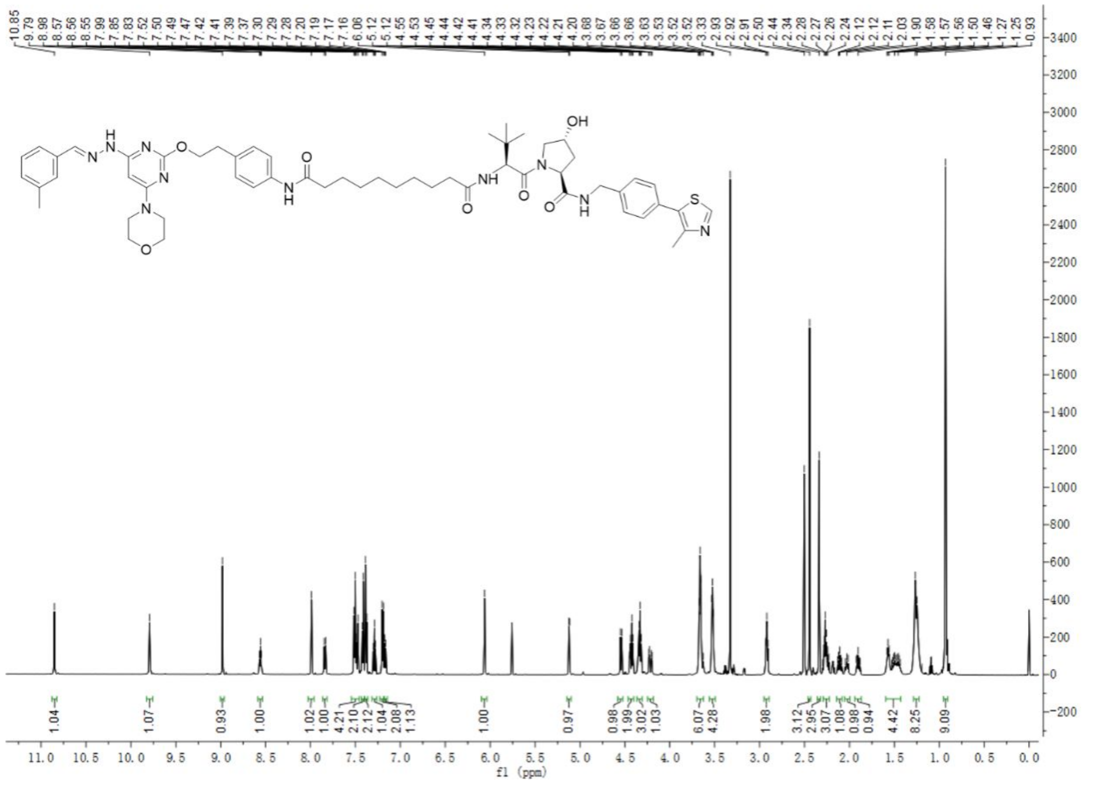

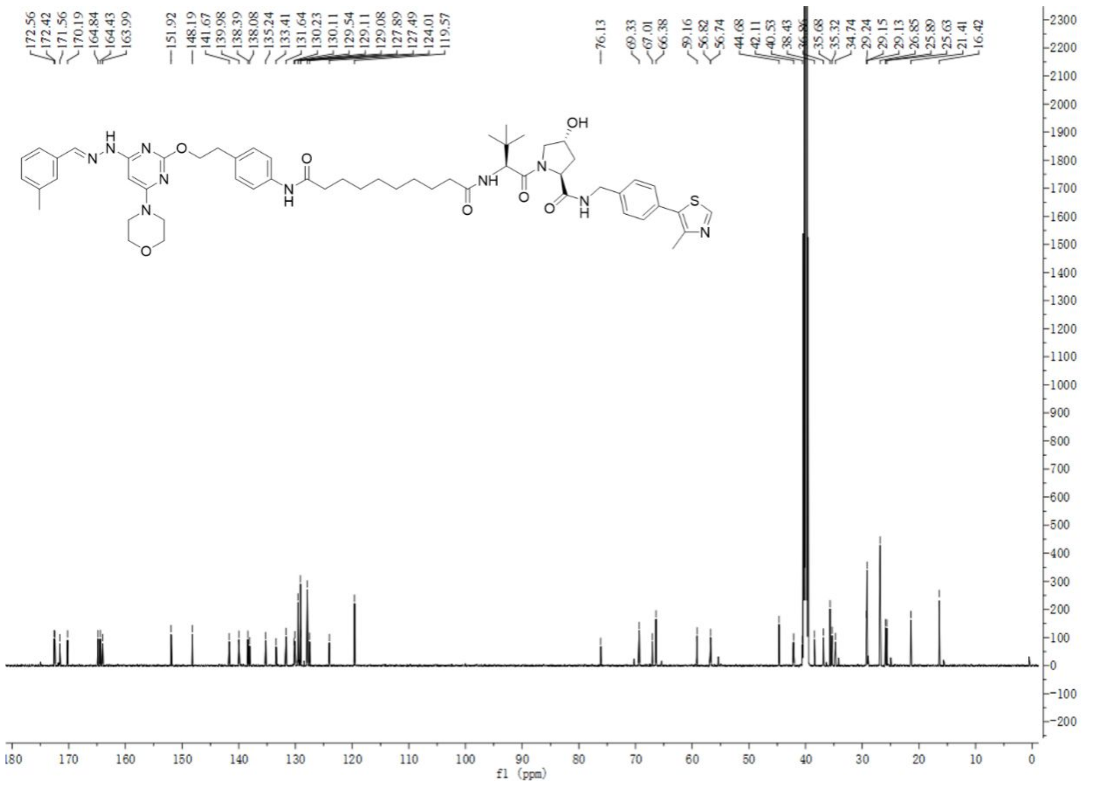

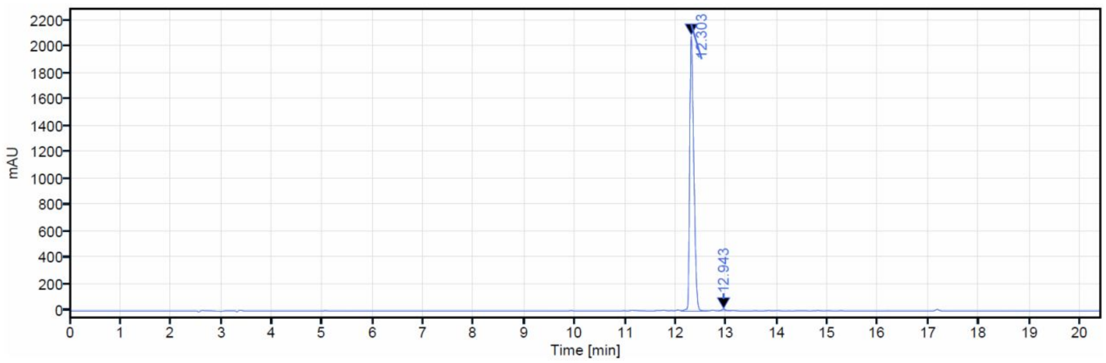

Signal: DAD1A,Sig=254,4 Ref=off

RT [min]	Type	Width [min]	Area	Height	Area%	Name
12.303	VB	0.51	11762.95	2084.50	99.35	
12.943	BV	0.24	77.33	14.17	0.65	
		**Sum**	11840.29		100.00	

Compound **12i**

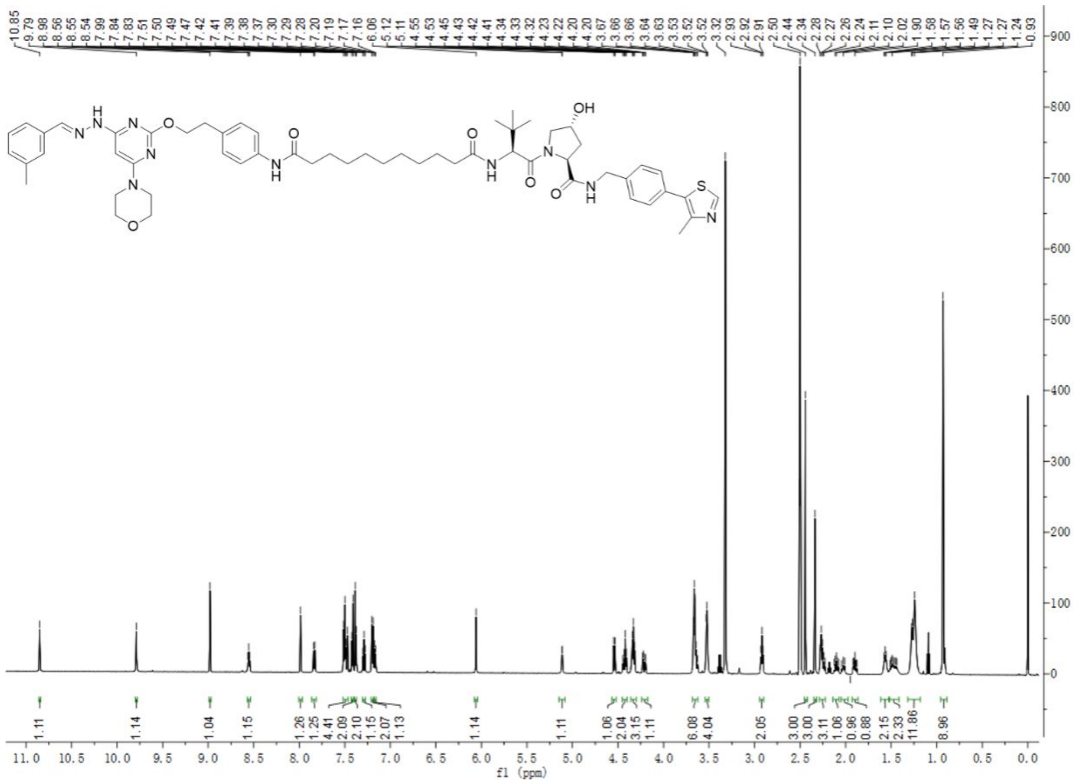

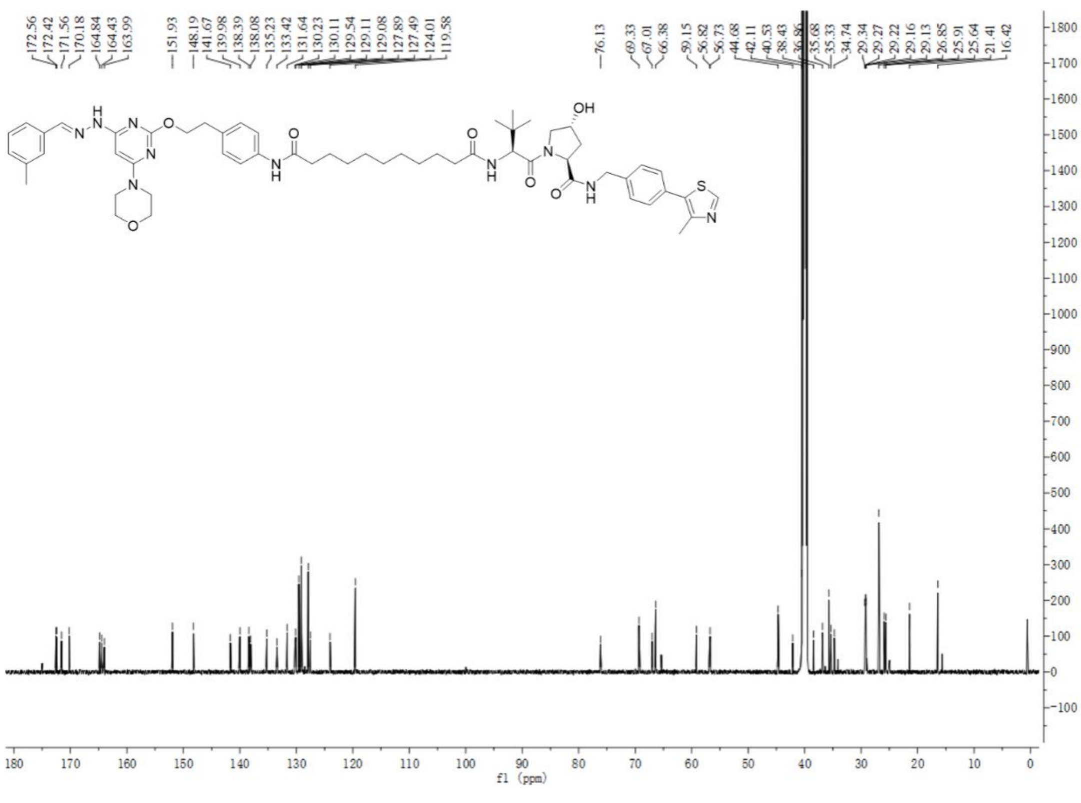

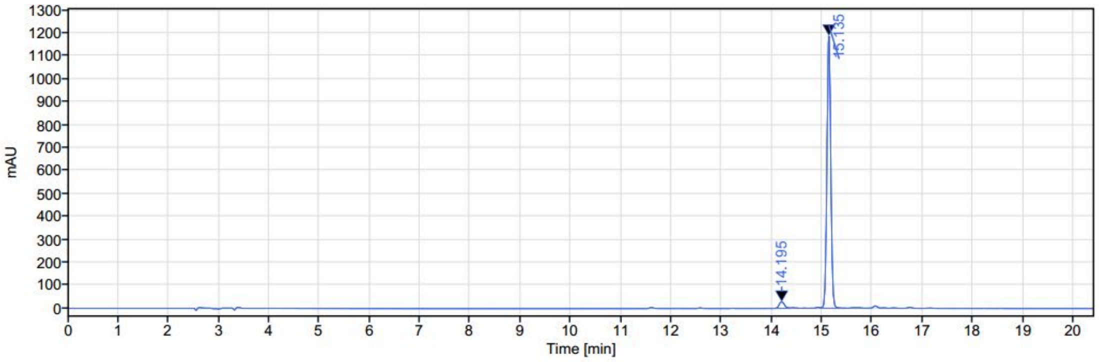

Signal: DAD1A,Sig=254,4 Ref=off

RT [min]	Type	Width [min]	Area	Height	Area%	Name
14.195	BV	0.37	209.99	27.74	3.19	
15.135	VB	0.55	6364.19	1187.27	96.81	
		**Sum**	6574.18		100.00	

Compound 12j

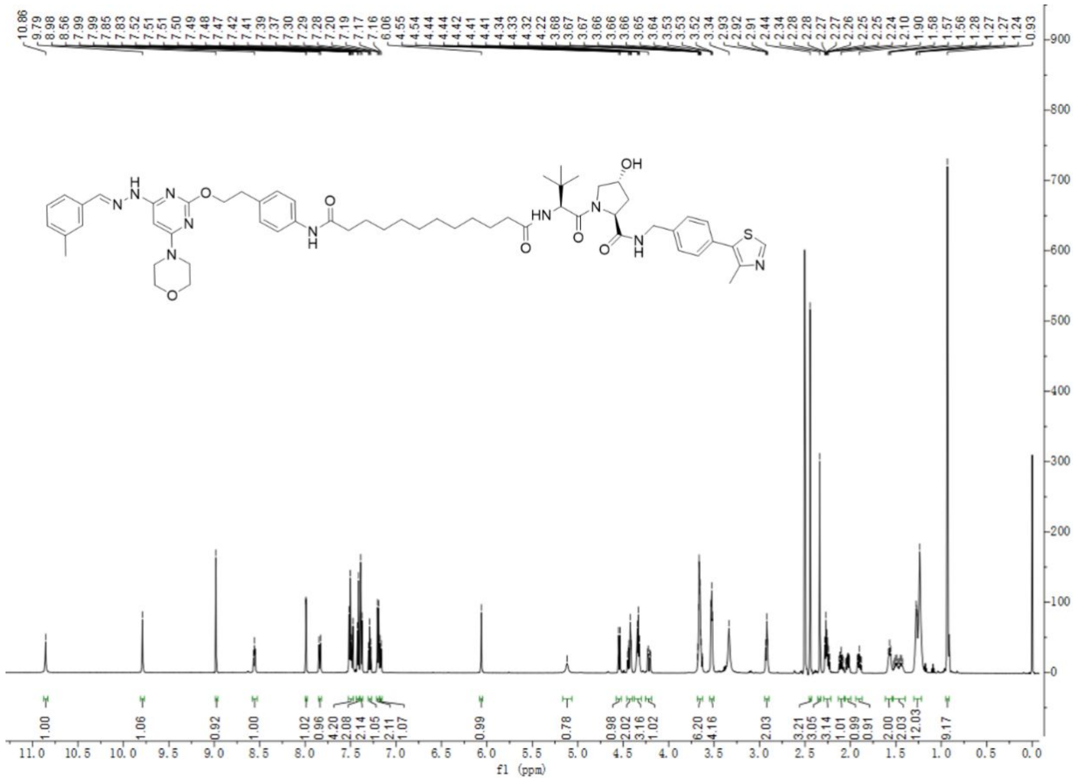

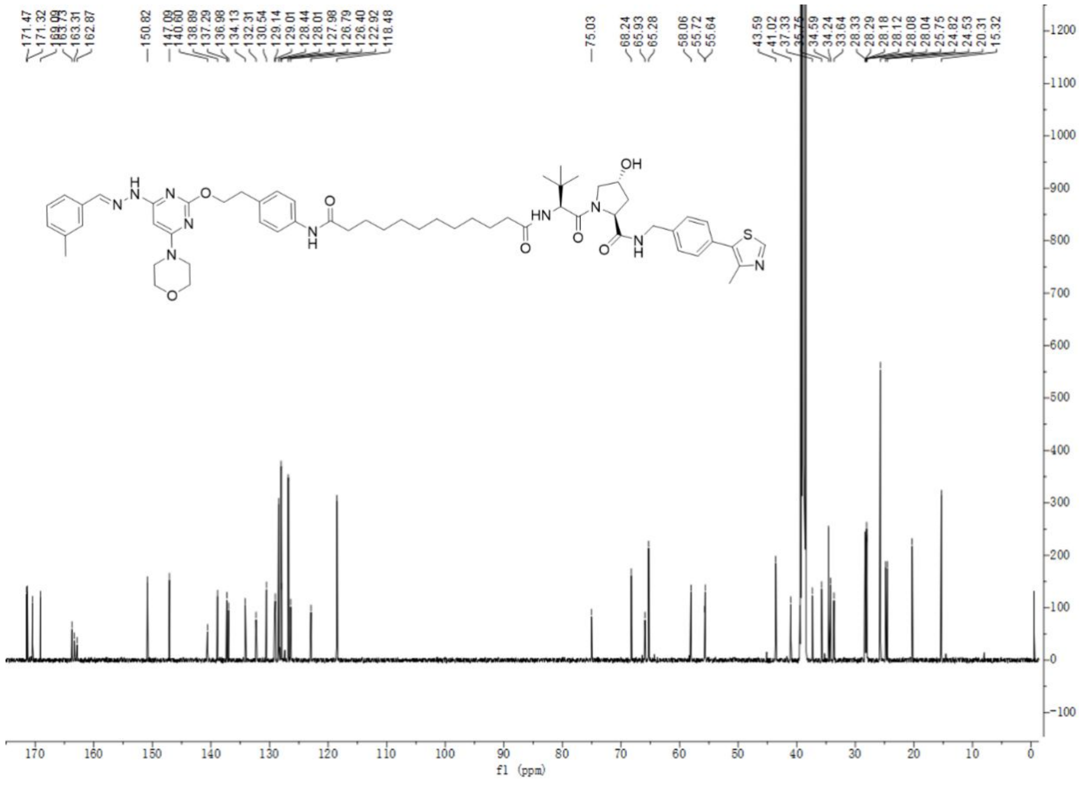

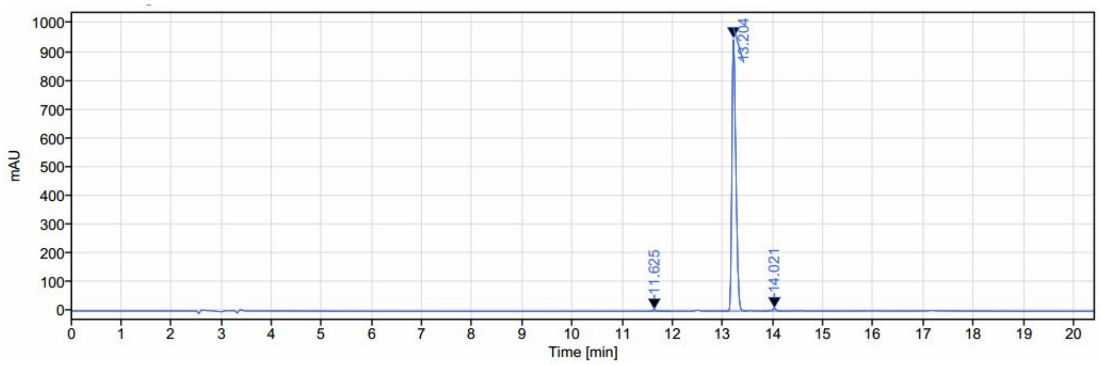

Signal: DAD1A,Sig=254,4 Ref=off

RT [min]	Type	Width [min]	Area	Height	Area%	Name
11.625	BB	0.46	26.19	4.53	0.50	
13.204	BV	0.53	5207.71	947.49	98.53	
14.021	BB	0.37	51.76	9.03	0.98	
		**Sum**	5285.66		100.00	

Compound **13a**

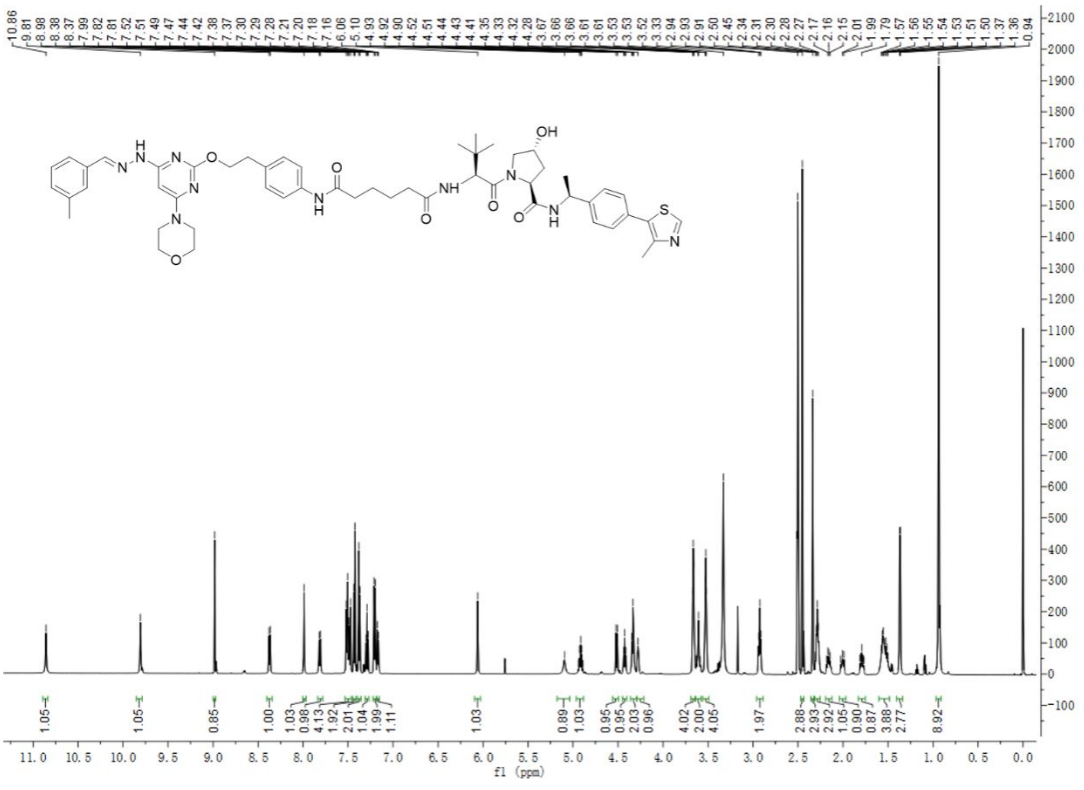

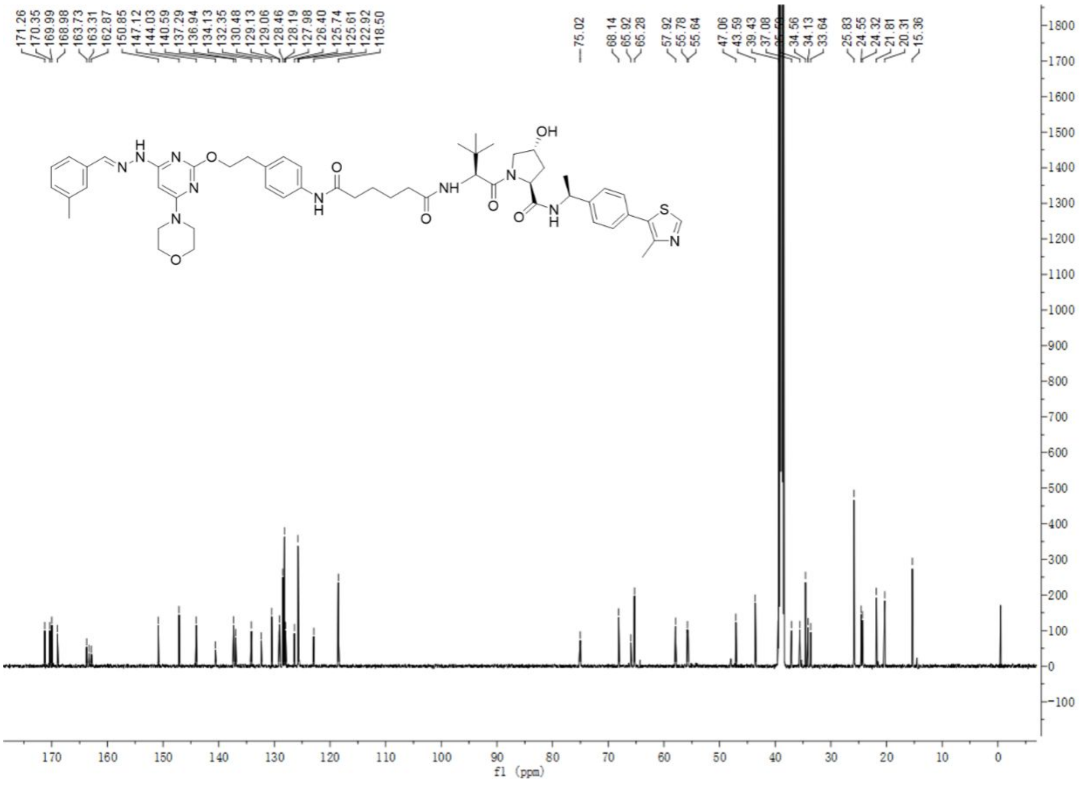

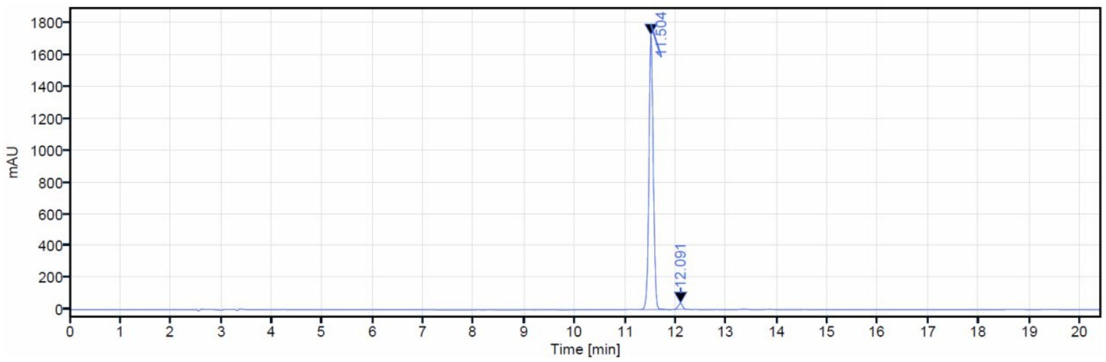

Signal: DAD1A,Sig=254,4 Ref=off

RT [min]	Type	Width [min]	Area	Height	Area%	Name
11.504	VM m	0.59	10338.98	1726.43	97.91	
12.091	MM m	0.32	220.46	37.12	2.09	
		**Sum**	10559.44		100.00	

Compound **12dN**

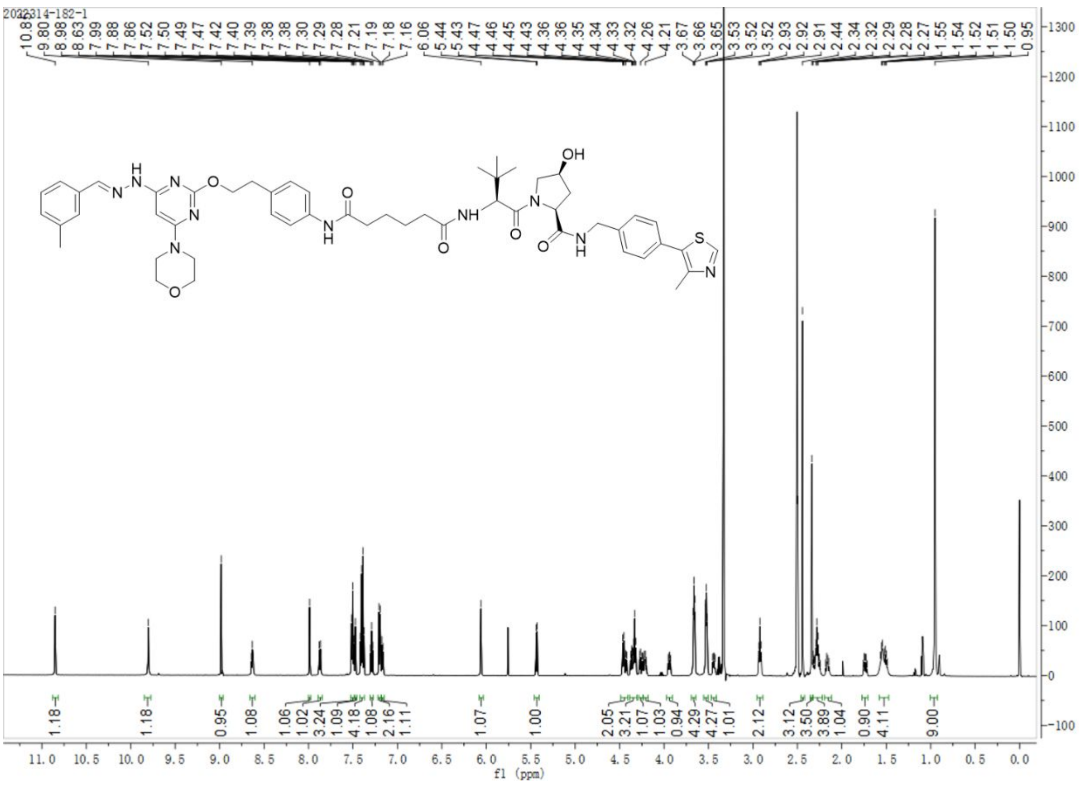

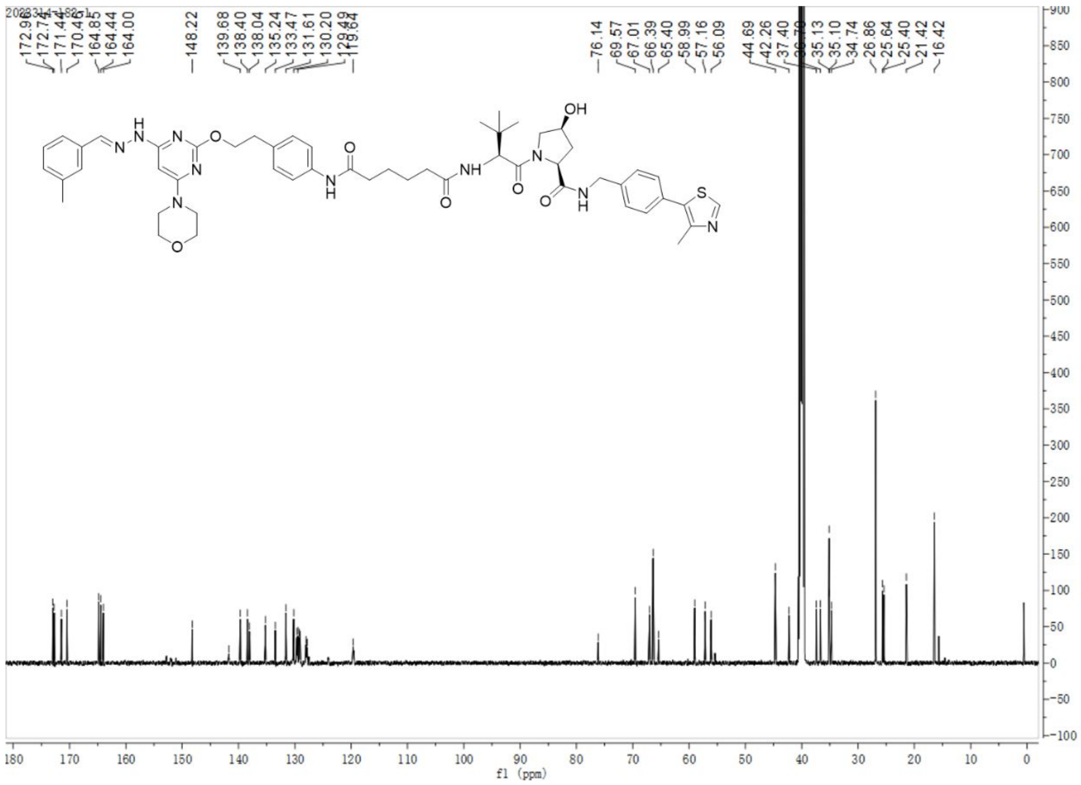

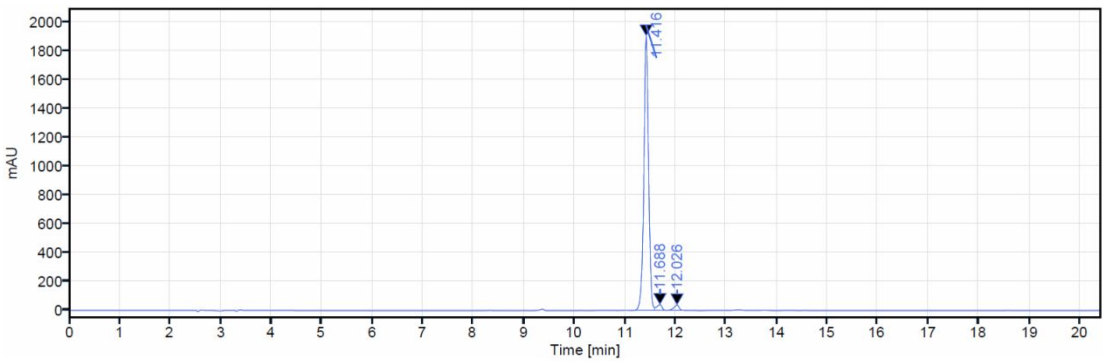

Signal: DAD1A,Sig=254,4 Ref=off

RT [min]	Type	Width [min]	Area	Height	Area%	Name
11.416	MV m	0.45	12348.51	1898.63	95.44	
11.688	VV	0.23	304.65	40.06	2.35	
12.026	VB	0.42	285.92	37.39	2.21	
		**Sum**	12939.08		100.00	
